# Improved Dye Survival in Expansion Microscopy through Stabilizer‐Conjugated Linkers

**DOI:** 10.1002/chem.202202404

**Published:** 2022-09-29

**Authors:** Gang Wen, Volker Leen, Yuqing Jia, Taoufik Rohand, Johan Hofkens

**Affiliations:** ^1^ Department of Chemistry KU Leuven Leuven 3001 Belgium; ^2^ Chrometra Kortenaken 3470 Belgium; ^3^ Department of Cell and Chemical Biology Leiden University Medical Center Einthovenweg 20 2333 ZC Leiden The Netherlands; ^4^ Laboratory of Analytical & Molecular Chemistry Faculty Polydisciplinaire of Safi Department of Chemistry University Cadi Ayyad 46000 Safi Morocco; ^5^ Max Planck Institute for Polymer Research 55128 Mainz Germany

**Keywords:** expansion microscopy, multifunctional molecules, organic dyes, radicals, stabilizers

## Abstract

Expansion microscopy (ExM) has been widely used to detect biomolecules in cultured cells and tissue samples due to its enablement of super resolution imaging with conventional microscopes, via physical expansion of samples. However, reaction conditions inherent to the process bring about strong fluorescent signal loss during polymerization and digestion and thus limit the brightness of the signal obtained post expansion. Here, we explore the impact of stabilizer‐containing organic fluorophores in ExM, as a mitigation strategy for this radical‐induced dye degradation. Through direct conjugation of 4‐nitrophenylalanine (NPA) to our previously developed trifunctional reagents, we validate and demonstrate that these multifunctional linkers enable visualization of different organelles with improved fluorescent intensity, owning to protection of the dyes to radical induced degradation as well as to photoprotection upon imaging. At this point, we cannot disentangle the relative contribution of both mechanisms. Furthermore, we report anchoring linkers that allow straightforward application of NPA or Trolox to commercially available fluorophore‐conjugated antibodies. We show that these anchoring linkers enable complete retention of biological targets while increasing fluorophore photostability. Our results provide guidance in exploring these stabilizer‐modified agents in ExM and methods for increased signal survival through the polymerization steps of the ExM protocols.

## Graphical abstract

**A series of** stabilizer‐containing multifunctional molecules has been developed and evaluated in expansion microscopy. These multifunctional molecules not only enable effective protection of dyes from radicals during polymerization, but also improve photostability while showing no negative impact on polymerization.

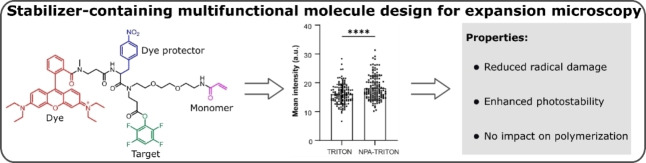

## Introduction

Expansion microscopy (ExM) is an alternative super‐resolution technique, making it possible to obtain super‐resolution imaging on standard fluorescent microscopes.^[1]^ By anchoring the sample into a swellable hydrogel and physically magnifying the sample by ∼4.5 times in a linear direction, ExM allows researchers to achieve ∼70 nm resolution with conventional diffraction‐limited microscopes. Due to its accessibility and performance, over the past six years, different ExM variants have been developed with widespread use in the imaging of biomolecules, for example, proteins,[^1b^, ^2^] RNA,[^1c^, ^3^] lipids,^[4]^ and glycans,^[5]^ in cell or tissue samples. For example, through increasing the achieved expansion factor, such as iterative expansion microscopy (iExM)^[6]^ and x10 expansion microscopy^[7]^ or combining ExM with traditional super‐resolution microscopy technologies, such as SIM^[8]^ and STED,^[9]^ lateral spatial resolutions of 25, 25, 30, and even <10 nm were obtained, respectively.

**Figure 1:**
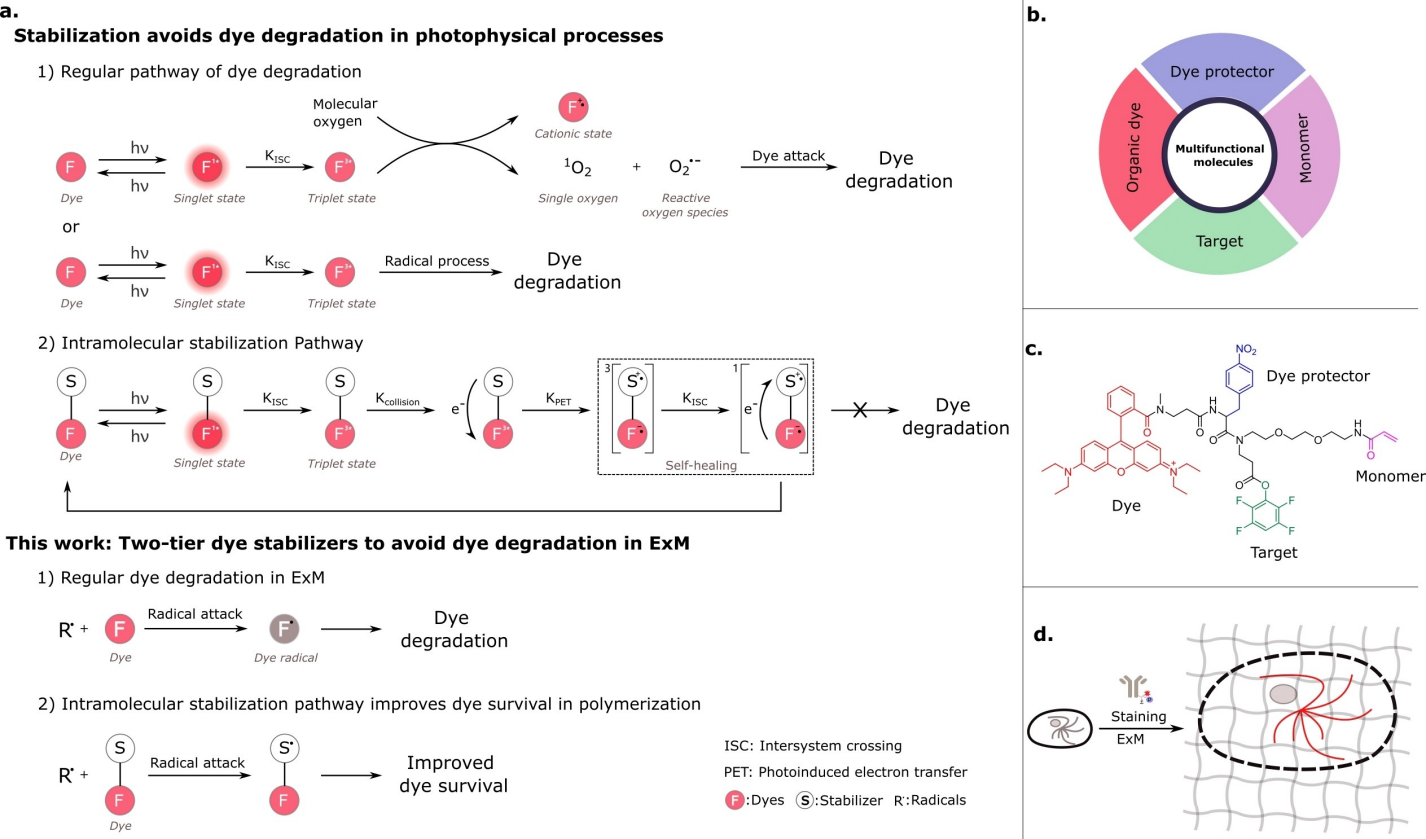
Schematic of stabilizer‐containing multifunctional molecules in ExM. (a) Design concept of protection of organic dyes from radicals via direct conjugation of stabilizers to dyes. Top, 1) Regular pathways of dye degradation upon photoexcitation. A fluorophore in the triplet state can react with molecular oxygen to produce reactive oxygen species and a non‐fluorescent cationic state of the fluorophore via electron transfer or via energy transfer for singlet oxygen. Dye degradation takes place when singlet oxygen or reactive oxygen species react with the fluorophore, thereby disrupting the conjugated system. In addition, dye degradation might also take place via a radical process. 2) Intramolecular dye repair process using stabilizers. The generated fluorophore triplet state is quenched by stabilizers via photoinduced electron transfer, followed by intersystem crossing and back electron transfer. By this process, organic fluorophores are recovered to the fluorophore ground state. Bottom, possible intramolecular stabilization pathway of stabilizer‐modified dyes against radicals produced in ExM. (b) Design of tetrafunctional molecules. (c) Example structure of tetrafunctional molecules. (d) Application of multifunctional molecules in ExM.

However, in most ExM variants, free radical‐induced dye destruction is inevitable during the polymerization step of forming the sodium polyacrylate/polyacrylamide backbone of the hydrogel.[^1^, ^2^, ^6a^, ^10^] The highly reactive nature of the radical intermediates leads to side reactions, where dye scaffolds are destroyed. All organic dyes suffer from this problem, though to a varying degree and the popular cyanine dyes (e. g., Cy3, Cy5) are almost completely destroyed during the polymerization step in ExM. This issue largely reduces the fluorescent signal intensity obtained after expansion and thus limits the imaging quality that can be achieved in ExM. Parallels can be drawn between the mechanisms of dye degradation in polymerization and the well‐studied radical mediated degradation of organic dyes in solution, through the formation of dark states (triplet‐ and/or radical‐state), leading to dye degradation. In the latter, the addition of radical scavengers, protecting groups and photostabilizers has been extensively explored to protect organic dyes over prolonged and high‐resolution imaging (Figure 1a). For example, nitrobenzyl alcohol (NBA) and Trolox enable protection of organic fluorophores from reactive oxygen species and radicals via direct conjugation of these groups to organic dyes.^[11]^ Here, we explore the impact of introducing such protecting groups in the vicinity of organic fluorophores as a protector against free radicals during the ExM polymerization step.

To investigate whether such protecting moieties are able to lead to increased dye survival, without hampering radical chain propagation and overall polymerization, and inspired by our recently developed trivalent linkers (TRITON), we design and synthesize a series of multifunctional molecules, including a fluorescent label, a monomer unit, a targeting moiety and a unit for intramolecular dye stabilization (Figure 1). Intramolecular photostabilization has been identified as an effective method to improve fluorophore photophysical properties, for example, increasing photostability, brightness, and reducing photoblinking.^[12]^ These molecules allow not only targeting, labeling and grafting biomolecules, but also effectively protecting fluorescent labels against the polymerization process and enhancing photostability. We then evaluate their performance in immunostaining experiments against microtubules. Furthermore, we also synthesize NPA‐ or Trolox‐modified anchoring linkers and explore their applications in ExM.

## Results and Discussion

**Figure 2:**
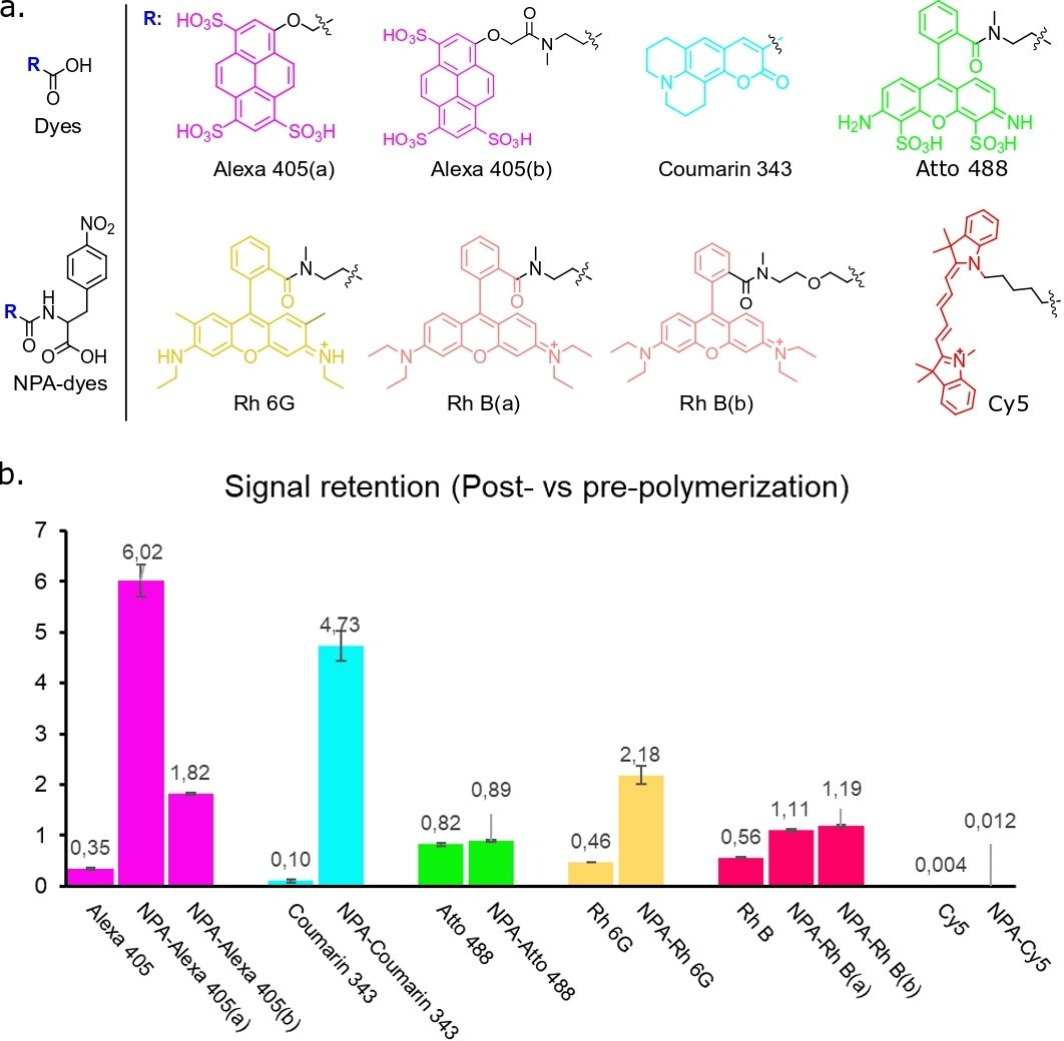
Comparison of signal retention of regular organic dyes and NPA‐modified dyes in the polymerization step. (a) Chemical structures of organic dyes with or without NPA modification. (b) Retention of fluorescence of synthetic dyes after polymerization. Bars represent the mean value and error bars represent the standard deviation. Signal retention is calculated via dividing fluorescent signal intensity post‐polymerization by signal intensity pre‐polymerization (mean±standard deviation, n=3 samples each).

Among various classes of such stabilizers, 4‐Nitrophenylalanine (NPA) is a convenient molecule, enabling the conjugation of biomolecules with stabilizer‐modified fluorophores in a simple step.^[13]^ Hence, we designed and synthesized a series of NPA‐modified organic fluorophores with different emission wavelengths (Figure 2a), ranging from 400 nm to 700 nm. The main measure of evaluating the performance of the constructs and the impact on different fluorophores is via determination of the absolute quantum yield. Additionally, considering that the distance between the fluorophore and stabilizers is a pivotal factor for the self‐healing properties through electron transfer,^[12c]^ we also conjugated Alexa 405 and rhodamine B with NPA using different linkers. We then assessed their performance against the free radical‐induced polymerization process by mixing them in the solutions typically used for hydrogel formation in ExM.

Through comparing the fluorescent signal intensity post‐polymerization vs. pre‐polymerization (n=3, Figure 2b and Table S1), we found that NPA provided clear protection of organic fluorophores from free radicals during the polymerization step. For example, the coupling of NPA to Alexa 405 substantially increased signal retention from 0.35 (Alexa 405, signal retention=signal intensity_post‐polymerization_/signal intensity_pre‐polymerization_) to 6.02 (17‐fold, NPA‐Alexa 405 (a), signal retention=signal intensity_post‐polymerization_/signal intensity_pre‐polymerization_). In line with literature observations, the protecting effect reduced with increasing distance between dye and NPA (as observed in NPA‐Alexa 405(b), Figure 2b). NPA‐coumarin 343 also showed the high signal retention of 4.73, comparing to 0.1 of coumarin 343. Although Atto 488 is generally considered a stable dye and commonly used in ExM, dye degradation is taking place to a minor extent, as we noticed that NPA could still slightly increase its signal retention to 0.89 during the polymerization process. As for rhodamine dyes (Rh 6G and Rh B), signal retention increased 5 and 2‐fold, respectively. Interestingly, we did not notice an apparent difference of signal retention when using a longer linker between Rh B and NPA (seen in NPA−Rh B(b)). In addition, although NPA−Cy5 showed a slight improvement of signal retention of Cy5 against free radicals, its signal retention was still insufficient for using this dye class in ExM. Furthermore, it is worth noting that we observed a blue shift of the excitation spectrum of coumarin 343 and its NPA‐analogue when transferring from water to polymer (Figure S1). As this impacts the overall excitation efficiency of the dye, evaluation of excitation maximum within its ExM matrix can contribute to optimal signal intensity as well.

**Figure 3:**
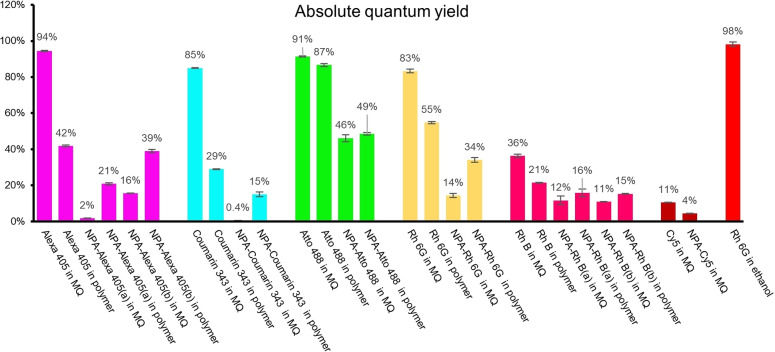
Quantified absolute quantum yield of organic dyes. All absolute quantum yields are measured in deionized water (abbreviated as MQ) or polymer. Control experiment: absolute quantum yield of Rh 6G in ethanol is measured and used as a reference. Bars represent the mean value and error bars represent the standard deviation. All data are from three independent experiments (mean±standard deviation).

To confirm a causal role for the NPA in these signal improvements, we ensured that dye aggregation in the aqueous solution could be ruled out as a competing factor. Through comparing the spectral deviations of absorption spectra between the parent solution and a diluted sample (6 times, Figure S2),^[14]^ we found that in most NPA‐modified fluorophore cases, there was no dye aggregation present in the aqueous solution, with Rhodamine 6G as notable exception. Therefore, we rationalized that this improved signal retention of NPA‐dyes was due to a quenching effect of NPA, resulting in a reduced quantum yield. In a less polar system, for example, the ExM polymer, we then observed a de‐quenching effect, resulting in a higher quantum yield. To substantiate this, we compared absolute quantum yield (Φ_f_) of all synthetic organic fluorophores in water and in polymer (Figure 3).

To investigate the efficiency of absolute Φ_f_ obtained in this method, we first performed a control experiment in which the absolute Φ_f_ of Rh 6G in ethanol was determined as 0.98 (n=3, Figure 3 and Table S2), in line with literature values (0.94–0.95).^[15]^ For the regular dyes without NPA modification, the quantum yield largely decreased from water to polymer, but for NPA‐modified dyes, we noticed an increase of the quantum yield in polymer, compared water (Figure 3 and Table S2). For example, the absolute quantum yield of Alexa 405 in water was evaluated as 0.94, and it dropped to 0.42 in polymer. In contrast, after binding NPA to Alexa 405, although the quantum yield sharply reduced to 0.02 (seen in NPA‐Alexa 405(a), Figure 3 and Table S2), this data showed a clear increase to 0.21 after polymerization. As expected, we observed that the signal retention of six different organic fluorophores showed a similar tendency as the absolute quantum yields obtained in the polymerization process. In addition, through comparing the quantum yields between NPA‐Alexa 405(a) and NPA‐Alexa 405(b), this quenching effect lowered when increasing the distance between the fluorophore and NPA.

**Figure 4:**
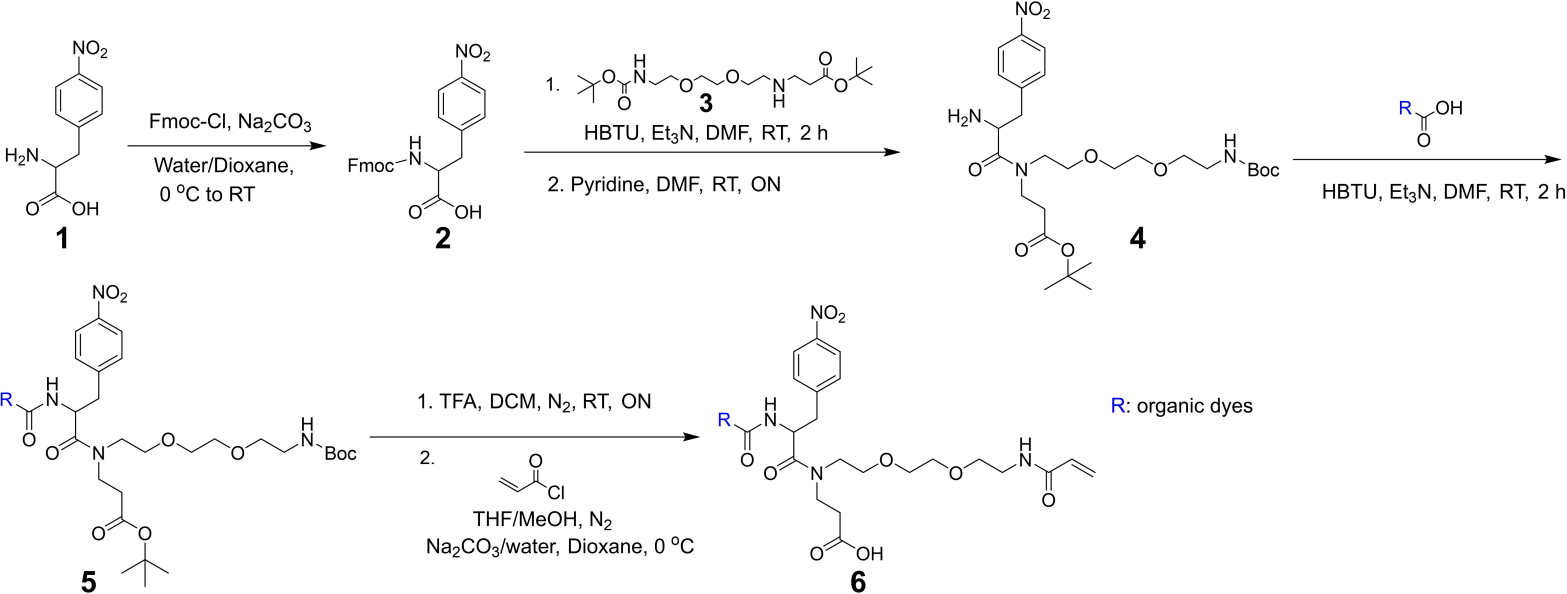
An optimized synthetic routine of tetrafunctional molecules.

We recently reported various trifunctional linkers (TRITON), which were successfully applied to detect small biomolecules such as actin and lipid in ExM. Considering the photoprotective properties of NPA, we developed a range of tetrafunctional molecules via introducing the NPA group to our previous TRITON linkers (Figure 1). These molecules not only enable targeting, labeling and grafting of biomolecules, but also simultaneously improve the fluorescent signal retention against free radicals, resulting from the polymerization step. To simplify the synthesis, we designed and optimized the synthetic routine (shown in Figure 4). The synthesis was started from the commercially available 4‐Nitro‐L‐phenylalanine (**1**), which was first converted into a Fmoc‐protected derivative (**2**). This derivative (**2**) was then reacted with the linker **3** under the coupling condition with HBTU, followed by deprotecting Fmoc to yield the key intermediate **4**. The primary amine of intermediate **4** was then used for coupling with the carboxyl group of different organic fluorophores, affording the intermediate **5**. Finally, the deprotection of the intermediate **5** was followed by selectively reacting with acryloyl chloride to obtain the desired product **6**.

**Figure 5:**
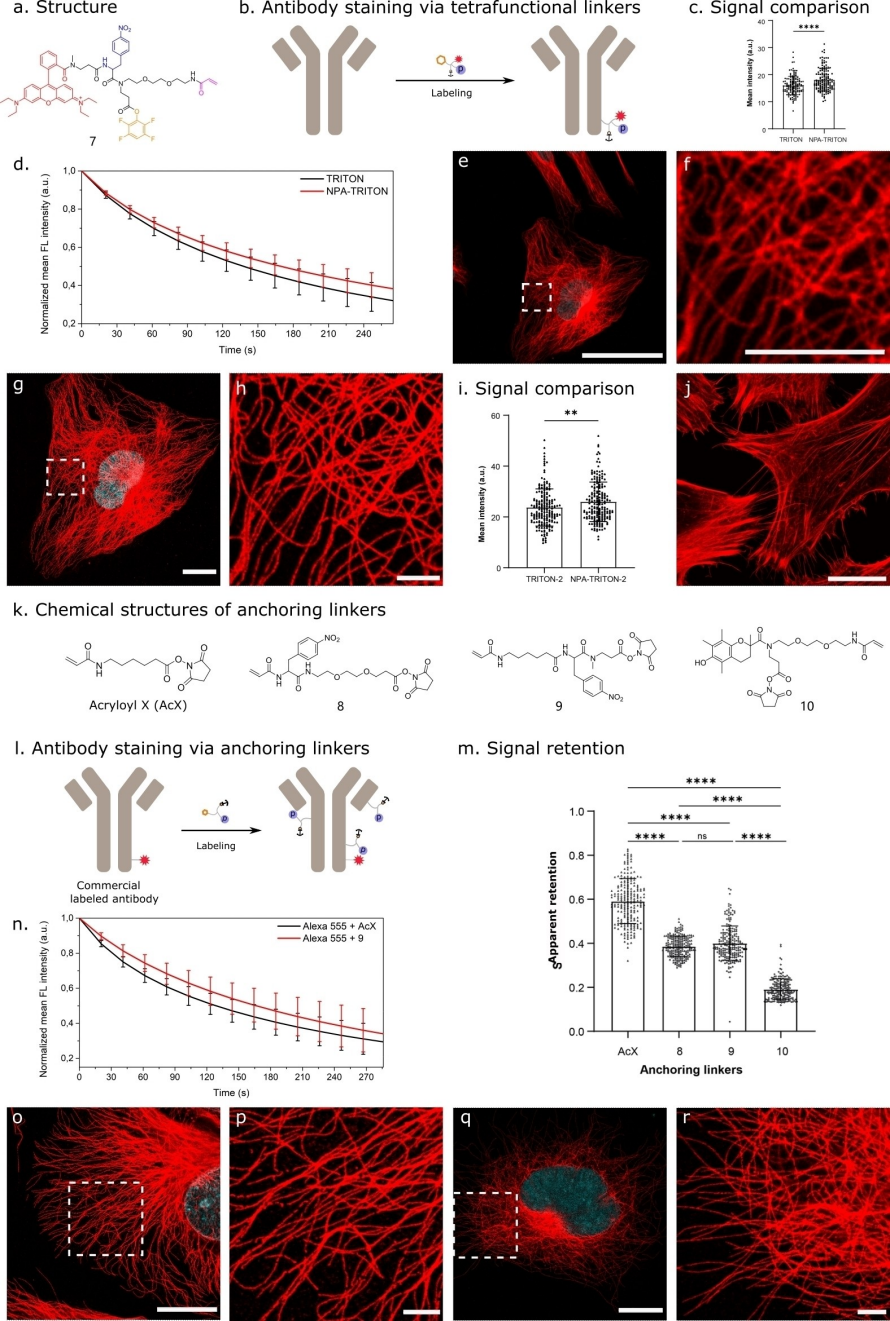
Immunostaining experiments using tetrafunctional molecules or NPA‐modified anchoring linkers. (a) Chemical structure of the representative tetrafunctional molecule (compound **7**, Rhodamine B, NPA, acryloyl and tetrafluorophenyl). (b) Schematic diagram of staining antibody using tetrafunctional molecules. (c) Comparison of mean fluorescence intensity of microtubules achieved post‐digestion using TRITON (Rh B, acryloyl and TFP) or NPA‐TRITON (compound **7**). Bars represent the mean value and error bars represent the standard deviation (n=140 from three independent samples.). Statistical significance is assessed by t‐test. **** p<0.0001. From left to right, mean values are 16.02±3.39 and 18.14±4.13, respectively. (d) Normalized mean fluorescence intensity of microtubules post‐expansion in TRITON and NPA‐TRION cases under continuous 567 nm laser illumination with a laser power of 48.6 μW (63×water immersion objective, illumination area: 79.93 μm×79.93 μm, n=18 from three independent samples each). (e–h) Images obtained in the immunostaining experiment with the represented compound **7**. (e) Pre‐expansion image of microtubules. (f) Zoom in on high‐lighted region in (e). (g) Post‐expansion image of the same cell. (h) Zoom in on highlighted region in (g). (i) Comparison of mean fluorescence intensity of actin filaments achieved post‐digestion using TRITON‐2 (Rh B, acryloyl and phalloidin) or NPA‐TRITON‐2 (Rh B, NPA, acryloyl, and phalloidin). Bars represent the mean value and error bars represent the standard deviation (n=195 from three independent samples.). Statistical significance is assessed by t‐test. ** p<0.01. From left to right, mean values are 23.73±7.31 and 25.94±7.77, respectively. (j) Post‐expansion image of actin filaments. (k) Chemical structures of three different anchoring linkers. (l) Schematic diagram of staining commercial fluorophore‐labeled antibodies using various anchoring linkers. (m) Comparison of apparent retention of Alexa 555 in the polymerization step using different anchoring linkers (apparent retention=signal intensity_post‐polymerization_/signal intensity_pre‐polymerization_). Bars represent the mean value and error bars represent the standard deviation. Statistical significance is assessed by one‐way ANOVA test. **** p<0.0001, ns (non‐significant)=0.1812. From left to right, mean values are 0.59±0.10 (mean ± standard deviation), 0.39±0.05, 0.40±0.08, 0.19±0.05, respectively. (n) Normalized mean fluorescence intensity of microtubules post‐expansion in AcX and compound **9** cases under continuous 553 nm laser illumination with a laser power of 54.5 μW (63×water immersion objective, illumination area: 79.93 μm×79.93 μm, n=17 from three independent samples each). (o) Post‐expansion image of microtubules with Alexa 555‐conjugated secondary antibodies and compound **8**. (p) Zoom in on high‐lighted region in (o). (q) Post‐expansion image of microtubules with Alexa 555‐conjugated secondary antibodies and compound **9**. (r) Zoom in on high‐lighted region in (q). Scale bars: 50 μm (e,g,j,o,q), 10 μm (f,h,p,r).

To evaluate the performance of our tetrafunctional molecules in a biological setting, we performed immunostaining experiments against microtubules (Figures 5a and b). In these experiments, secondary antibodies were labeled with a Rh B‐containing tetrafunctional molecule (compound **7**, Figure 5a) followed by gelation, digestion and expansion process in line with previously described protocols.^[3a]^ We compared the fluorescence intensity of microtubules stained with the original TRITON linker (no photoprotection of the pre‐coupled dye, Rh B, acryloyl and TFP, structure details in the literature^[4b]^) or the NPA‐TRITON linker (identical to TRITON linker but now with the protection group close to the dye, compound **7**) pre‐ and post‐polymerization. To achieve this, we labeled secondary antibodies with TRITON or NPA‐TRITON (compound **7**) at a similar density (labeling density per antibody: 2.69 vs. 2.53). A quenching effect of NPA in immunostaining experiments was observed, as introducing NPA spatially close to Rh B resulted in a lower fluorescent intensity (mean intensity: 30.65±7.49 vs. 37.01±9.53, n=189 from three independent samples, Figure S3). However, to our delight, we found that the mean fluorescence intensity of microtubules after digestion was higher than in the case of TRITON (18.14±4.13 vs. 16.02±3.39, n=140 from three independent examples each, Figure 5c), showing the effective protection of RhB on radical‐induced dye damage/photodegradation. In addition, the expansion process largely increases the sample volume to scan, photostability is also an important factor to consider when constructing 3D images in expanded samples, especially in tissues.^[16]^ To assess the influence of NPA on dye photostability, we tracked the decay of fluorescence intensity post‐expansion under continuous laser illumination on confocal microscopes. We demonstrated that NPA‐TRITON showed an increased photostability post‐expansion, compared to TRITON linkers (Figure 5d), in line with the previous observations.^[17]^ For instance, after continuous 567 nm laser illumination for 2 minutes, retained fluorescent intensity ratio of microtubules in the NPA‐TRITON case was higher than its in TRITON case (0.58±0.04 vs. 0.53±0.06, n=18 from three independent samples each, Figure S4). When compared to the largely improved photophysical parameters evaluated with TIRF,^[12]^ we reasoned that this lower photostability effect in cellular studies might due to the different excitation intensity along the z‐axis between TIRF and confocal setups, additional interactions of fluorophores with redox‐active molecules in the cellular system and the impact of oxygen in system.^[17]^ As a result, we observed a clear and continuous signal of microtubule fibers in the expanded specimens (Figures 5e–h). After recalculating the full width at half‐maximum (FWHM) with the expansion factor of 3.47 (Figure S5), we obtained an effective resolution of 107±9 nm (mean ± standard deviation, n=135, Figure S6), which was consistent with the theoretical resolution of 106 nm, calculated following the Rayleigh criterion. In addition, we also explored this strategy in the visualization of actin filaments post‐expansion. Through staining actin filaments with the same concentration of TRITON‐2 (Rh B, acryloyl, and phalloidin, detailed structure in Scheme S1) or NPA‐TRITON‐2 (Rh B, NPA, acryloyl, and phalloidin), we found that the NPA‐modified phalloidin TRITON linker also resulted in improved signal intensity after digestion, compared to the common TRITON linker (25.94±7.77 vs. 23.73±7.31, n=195 from three independent examples each, Figure 5i). This is in line with the improved fluorescence intensity obtained in immunofluorescence of microtubules. After expansion, we can visualize actin filaments at nanoscale resolution (Figure 5j).

We set out to evaluate the performance of NPA on conventional fluorophore‐labeled antibodies. To readily introduce NPA to these fluorescent antibodies in ExM, we synthesized two NPA‐modified anchoring linkers (compound **8** and **9**) via binding NPA with succinimidyl ester of 6‐((Acryloyl)amino)hexanoic acid (Acryloyl‐X, SE, Life Technologies, also abbreviated AcX), commonly used in ExM (Figure 5k). In addition, we also synthesized an anchoring linker with Trolox (compound **10**), a seminal type of stabilizers (Figure 5k). To assess the performance of these anchoring linkers in ExM, we immunostained microtubules with commercially available Alexa 555‐labeled secondary antibodies, followed by staining with AcX or NPA‐ or Trolox‐modified linkers. The apparent fluorescence retention was then compared post‐polymerization in different cases (Figures 5l and m).

Interestingly, we found that samples stained with NPA linkers showed a lower apparent retention post‐polymerization, compared to those stained with AcX (apparent retention: overall signal retention is caused by radical‐induced fluorophore damage and stabilizer‐related fluorophore quenching). Specifically, the apparent retention of compound **8**‐stained samples was 0.39±0.05 (mean±standard deviation, n=211 cells from three independent samples, Figure 5m); and the apparent retention of compound **9**‐stained samples was 0.40±0.08 (mean±standard deviation, n=211), compared to 0.59±0.10 of the AcX case (n=211). We did not notice a significant impact of linker length on the apparent retention from comparing the performance of compound **8** and compound **9**. Samples stained with Trolox linker (compound **10**) showed the poor apparent retention of 0.19±0.05 (mean±standard deviation, n=200 cells from three independent samples). Among these anchoring linkers, we then chose compound **9** as a candidate to evaluate its influence on photostability and observed an increased photostability of Alexa 555 post‐expansion when grafting microtubules with compound **9**, providing a novel approach to introduce stabilizers to organic fluorophores (Figure 5n and Figure S7). In addition, we also tested the performance of compound **9** when staining microtubules with commercial Alexa 647‐labeled secondary antibodies, the similar result was obtained, showing no improvement of apparent retention post‐polymerization, comparing to the AcX case (Figure S8). We reasoned that the low apparent retention observed in the cases using NPA‐ or Trolox‐modified anchoring linkers may be due to the quenching effect of NPA or Trolox against Alexa 555 and Alexa 647. This quenching effect of NPA or Trolox has been reported in literature,[^12a^, ^13^] and is consistent with our quantum yield determination mentioned above (Alexa 555: Atto 488 analogue; Alexa 647: Cy5 analogue). Finally, considering that nitro‐substituted aromatics have been reported as polymerization inhibitors,^[18]^ we showed the absence of any negative impact on polymerization. To this end, we confirmed that no impact on expansion factor was noticed in the presence of and in the absence of NPA (3.25±0.06 (mean±standard deviation), or 3.29±0.07 vs. 3.27±0.06, Figure S9) and stained microtubules were completely retained after expansion (Figures S10–S11) upon using NPA linkers. As a result, a clear and continuous fluorescence signal of microtubules was observed post‐expansion (Figures 5o–r) without any sign of influence of NPA on polymerization. And the nitrophenyl group is demonstrated to be compatible with polymerization, though some degradation pathways are mentioned.^[19]^

## Conclusion

In this work, we report that NPA‐conjugated fluorophores show an increased fluorescent signal retention during the free‐radical polymerization process. To take advantage of this finding, we develop direct and indirect strategies to introduce NPA to organic dyes, for use in polymerization and in ExM. A series of tetrafunctional molecules is synthesized via the coupling of stabilizing moieties to previously described TRITON linkers, allowing the protection of organic labels in a direct and intramolecular way. We observe an increased fluorescent signal of microtubules and actin filaments post‐expansion while showing an increased photostability of parent dyes. Furthermore, considering the required use of anchoring reagents in ExM, we also synthesize three stabilizer‐modified anchoring linkers. Through comparing to the standard anchoring reagent (AcX), we validate and demonstrate that these anchoring linkers enhance the photostability of fluorescently labeled antibodies, while showing complete retention of stained microtubules in ExM. The actual radical stabilization processes at play remain elusive. Single electron transfer processes are well established for photostabilizers,^[20]^ and the radicals formed therein can even participate in further conjugate additions,^[21]^ such as the radical polymerization in ExM, thus leading to propagation and not termination. Since the results are somewhat obscured by differential quenching rates over various organic dye classes, we have initiated a photophysical study to investigate dye structure dependent interaction with the stabilizers shown in this manuscript. This study will hopefully shed more light on the various underlying mechanisms and processes, but remains outside the scope of this paper, and will direct linker development and use within expansion microscopy. Given the clear effects on overall signal intensity after expansion, we are convinced that this research will guide and promote the further study and application of stabilizer‐modified agents in ExM.

## Experimental Section

**Labeling antibody with tetrafunctional molecules**: Tetrafunctional molecule‐labeled secondary antibodies were prepared following the protocol below. In brief, 75 μL of secondary antibodies (2 mg/mL) was washed with sodium borate buffer (1 M, pH 8.5, 400 μL per time) three times using Amicon Ultra 0.5 mL 50 kDa Centrifugal Filters (EMD Milli‐pore #UFC510096). To a solution of resulting antibody, a solution of tetrafunctional molecule (compound **7**, 1 μL, as a DMSO solution (85 mM)) and 20 μL sodium borate buffer were added and was incubated for 90 min at 37 °C on a shaker covered in aluminum foil. After incubation, antibodies were purified by a 0.5 ml 40 kDa Zeba desalting column (ThermoFisher #87766), using PBS as an eluent. The characteristic of functionalized antibody was performed through UV VIS analysis with the dye main absorption band as chromophore (Rhodamine B, λ_max_=570 nm, molar extinction coefficient 106000), Biodrop instrument. The labeling ratio of dye/antibody was evaluated as 2.53 and concentration of functionalized antibodies was determined as 18 μM. The antibodies were stored at 4 °C until use.

**Cell culture**: HeLa cells (ATCC) were cultured in Dulbecco's Modified Eagle Medium (DMEM; Life Technologies, high glucose (4.5 g/L), no glutamine, no phenol red) supplemented with 10 % (v/v) fetal bovine serum, 50 μg/mL gentamicin (Life technologies) and 1 % glutamax at 37 °C with 5 % CO_2_. When cells reached 70–80 % confluency, cells were washed three times with 1x DPBS (no calcium, no magnesium; Life Technologies), followed by detaching with 10×TrypLETM Enzyme (Life Technologies). Cells were then seeded on 22 mm×22 mm #1.5 coverslips at a density of 4×10^4^ cells/cm^2^ and cultured for 24 h.

**Microtubule and actin filament staining**: For microtubule imaging, cultured cells were then extracted twice for 30 seconds with pre‐heated MTSB (PEM Triton X, 80 mM PIPES pH 6.8, 5 mM EGTA, 1 mM MgCl_2_, 0.5 % Triton X‐100). Afterwards, the sample was fixed with a MTSB solution containing 0.5 % glutaraldehyde for 10 minutes and washed three times for 5 minutes with PBS before quenching with freshly prepared 0.2 % sodium borohydride in PBS at room temperature for 7 minutes. Cells were washed three times with PBS and the fixed Hela cells were blocked twice for 5 minutes with blocking buffer (PBS, 1 % BSA) followed by incubation with a solution of primary antibodies (mouse anti α‐tubulin, Abcam, ab7291) in staining buffer (PBS, 3 % BSA) at a concentration of 2 μg/mL for 1 h at room temperature and washed with PBS three times for 5 minutes each. Samples were incubated with tetrafunctional molecule‐labeled secondary antibodies (Goat Anti‐Mouse IgG, Abcam ab) for 1 h in staining buffer with a dilution of 1 : 50. After incubation, samples were rinsed twice with PBS and washed twice with blocking buffer for 5 minutes each. Finally, specimens were stained with 1 μg/mL DAPI and then washed three times with PBS prior to imaging. The same staining protocol was followed when staining with commercially available Alexa 555‐labeled secondary antibodies, except that the secondary antibodies was diluted to 100 times in staining buffer.

For actin imaging, cultured cells were fixed 10 min with 4 % paraformaldehyde (PFA) in PBS and washed three times for 5 minutes with PBS. The cells were then quenched with 100 mM NH_4_Cl in PBS for 20 min, followed by washing three times for 5 minutes with PBS. After permeabilization with 0.25 % Triton X‐100 in PBS for 15 min, they were washed three times for 5 minutes with PBS. The cells were then stained with 0.25 μM TRITON‐2 or NPA‐TRITON‐2 in PBS at room temperature for 1 h. After incubation, samples were rinsed twice with PBS and stained with 1 μg/mL DAPI and then washed three times with PBS prior to imaging. For grafting optimization of phalloidin TRITON molecules, the cells were incubated with AcX (0.1 mg/mL) in PBS overnight and then washed twice with PBS for 15 minutes each prior to the gelation step.

**ExM protocol using multifunctional molecules**: For samples stained with multifunctional molecules, gelation, digestion and expansion were performed as follows. Monomer solutions containing 2 M NaCl, 8.625 % (w/w) sodium acrylate, 2.5 % (w/w) acrylamide, 0.15 % (w/w) N,N’‐methylenebisacrylamide and 0.01 % 4‐hydroxy‐TEMPO in PBS were prepared and stored in an ice bath until further use. The gelation solution was prepared by diluting the concentrated stocks of tetramethylenediamine (TEMED, 10 % w/w) and ammonium persulfate (APS, 10 % w/w) in monomer solution to concentrations of 0.15 % (w/w) each. The samples were first washed with the freshly prepared gelation solution (80 μL) and the gelation process was performed on the gelation chamber with another 80 μL gelation solution at 37 °C for 1.5 h. Next, gels were digested with a solution of proteinase K (New England Biolabs, 8 units/mL) in digestion buffer (50 mM Tris (pH 8.0), 1 mM EDTA, 0.5 % TritonX‐100, 0.8 M guanidine HCl) overnight at room temperature. Afterwards, proteinase K was removed by washing two times with PBS and samples were re‐stained with 1 μg/mL DAPI followed by washing three times with PBS. The digested gels were expanded with double‐deionized H_2_O for 10 minutes. This step was repeated 3–4 times with fresh ddH_2_O until gels were fully expanded. For samples stained with the commercial Alexa 555‐labeled secondary antibody, stained samples were incubated with a solution containing acryloyl X (AcX, 0.1 mg/mL in PBS) or NPA‐modified anchoring linkers (0.15 mg/mL in PBS) at room temperature overnight. Next, samples were washed twice with PBS for 15 minutes each prior to the gelation step.

**Re‐embedding of expanded hydrogels**: To reduce the hydrogel drift during imaging, we immobilized the expanded gels by re‐embedding them in an uncharged polyacrylamide gel following the previous procedure. In brief, the glass bottom of 6 well plate (Cellvis, Product # : P06‐1.5H‐N) was treated with 1 mL bind‐silane solution (4 mL of ethanol, 100 μL of acetic acid, 3 μL of bind‐silane, 900 μL of double‐deionized H_2_O) at room temperature for 1 h. The solution was removed and the well was further dried with compressed air. The digested gel was then transferred to the prepared well and expanded with double‐deionized H_2_O for 10 minutes. This step was repeated 3–4 times with fresh ddH_2_O until gels were fully expanded. Afterwards, the expanded gel was rinsed with 2 mL of freshly prepared re‐embedding solution (re‐embedding solution: 3 % acrylamide, 0.15 % N,N′‐Methylenebisacrylamide, 0.05 % APS and 0.05 % TEMED in 5 mM Tris (pH: 10.5)) and covered with another 2 mL of re‐embedding solution at room temperature for 20 min. Next, the solution was completely removed and a clean 22 mm×22 mm #1.5 coverslip was placed on the top of the gel before moving to a container degassed with nitrogen, followed by incubation at 37 °C for 1.5 h. After incubation, the sample was washed three times with double‐deionized H_2_O, 20 minutes each while detaching the top coverslip after the second washing step.

**Evaluation of signal retention of organic fluorophores**: To assess the signal retention of fluorophores in the polymerization step, we compared their emission intensity using the same setups except the incubation systems: water or polymer. In brief, a tiny volume of fluorophore stock solutions was diluted using 600 μL water or 600 μL gelation solutions in the same concentrations in the Eppendorf tubes and quickly transferred into 0.8 mL Crimp Neck Vials (30×8.2 mm, clear glass, 1st hydrolytic class). The final concentrations of fluorophores were list in Table S1 and the absorbance of different fluorophores ranged from 0.10 to 0.30. Next, The gelation process was performed in a container, degassed with nitrogen at 37 °C for 1.5 h. For the same fluorophore, we measured its emission intensity in water and polymer using the same setups. Next, we calculated the signal retention by dividing the emission intensity of polymer system by the relevant emission intensity of water system.

**Evaluation of signal retention of dyes with NPA‐modified anchoring linkers**: The immunostaining protocol used for commercially Alexa 555‐labeled secondary antibody was mentioned above. After immunostaining, samples were first imaged, followed by incubation with acryloyl X (AcX, 0.1 mg/mL in PBS) or NPA‐modified anchoring linkers (0.15 mg/mL in PBS) at room temperature overnight. Next, samples were washed twice with PBS for 15 minutes each. Afterwards, the gelation process was performed as aforementioned and gelled samples were imaged again using the same parameters as pre‐imaging to determine the fluorescent signal retention.

**Determination of absolute quantum yield**: To determine absolute quantum yields of organic fluorophores, we used the F‐3018 Quanta‐ϕ integrating sphere setup in the Fluorolog®‐3 spectrofluorometer. The samples were prepared in the 0.8 mL Crimp Neck Vials (30×8.2 mm, clear glass, 1st hydrolytic class) as mentioned in the previous section. The parameters used in the measurement were shown in Table S2.

**Evaluation of dye aggregation**: As a rule, dye aggregation commonly cause an increase of absorption at the short‐wavelength shoulder of the maximum wavelength band. Therefore, to evaluate whether there is dye aggregation formed in the aqueous solutions, we compared the absorption spectra of fluorophores in different concentrations. In brief, the fluorophore was dissolved in water at the concentration that we used in the experiments measuring signal retention. The absorption spectrum was recorded using a disposable cuvette (1 cm, Kartell) in UV/VIS/NIR spectrometer (Lambda 950). The parent solution was diluted by a factor of two and six and the absorption spectra were re‐measured using the same cuvette. To compare the spectral deviations of samples with different concentrations, we also normalized the absorption spectra.

**Microscopy and image analysis**: Confocal Imaging was acquired on an inverted Leica true confocal scanner SP8 X system (Wetzlar, Germany) using a HCPLAPO CS2 63×water immersion objective (NA 1.2) for pre‐expansion images or a Fluotar VISIR 25×water immersion objective (NA 0.95) for post‐expansion images. The nuclei were stained with DAPI and activated by a 405 nm pulsed diode laser. A supercontinuum white light laser (SuperK EXTREME/FIANIUM, NKT photonics, Birkerød, Denmark) was used for rhodamine B and Alexa 555 excitation. Image analysis was performed with ImageJ after acquisition. Expansion factor was quantified by comparing the same areas of nuclei pre‐ and post‐expansion. The theoretical resolution was calculated following the Rayleigh criterion (r=0,61×λ_emission_/NA). The experimental image resolution was determined using ImageJ FWHM_line plugin with the Gaussian fitting and rescaled by expansion factor.

## Supporting Information

Additional data and experimental procedures including synthesis, NMR spectra, and mass spectra are provided in Supporting Information.

## Conflict of interest

The authors declare no competing financial interest(s).

1

## Supporting information

As a service to our authors and readers, this journal provides supporting information supplied by the authors. Such materials are peer reviewed and may be re‐organized for online delivery, but are not copy‐edited or typeset. Technical support issues arising from supporting information (other than missing files) should be addressed to the authors.

Supporting Information

## Data Availability

The data that support the findings of this study are available from the corresponding author upon reasonable request.
