# Supplementary material for: Improved Dye Survival in Expansion Microscopy through Stabilizer‐Conjugated Linkers
Source: Chemistry. 2022 Sep 29;28(66):e202202404. doi: 10.1002/chem.202202404 (PMC9828348; doi:10.1002/chem.202202404)
Supplement: Supplementary file 1 — Supporting Information [file CHEM-28-0-s001.pdf]

Chemistry–A European Journal

Supporting Information

Improved Dye Survival in Expansion Microscopy through Stabilizer-Conjugated Linkers

Gang Wen, Volker Leen, Yuqing Jia, Taoufik Rohand, and Johan Hofkens*

Table of Contents

1. Multifunctional molecule synthesis 1
2. Figure S1. Excitation and emission properties of organic fluorophores in water and polymer 16
3. Table S1. Parameters used for signal retention evaluation 16
4. Figure S2. Characteristic of NPA-modified organic fluorophores in water 17
5. Table S2. Φ_f values and parameters used for determination at room temperature 17
6. Figure S3. Comparison of pre-polymerization fluorescence intensity in immunostaining 18
7. Figure S4. Comparison of retained fluorescent intensity of expanded microtubules in TRITON and NPA-TRION cases 18
8. Figure S5. Expansion factor calculated in ExM 19
9. Figure S6. Quantification of resolution achieved in post-expansion images 19
10. Figure S7. Comparison of retained fluorescent intensity of expanded microtubules in AcX and compound 9 cases 19
11. Figure S8. Comparison of signal retention of Alexa 647 in the polymerization step using different anchoring linkers 20
12. Figure S9. Comparison of expansion factor obtained with different anchoring linkers in ExM 20
13. Figure S10. Quantification of anchoring efficiency of compound 8 in the immunostaining experiments against microtubules 21
14. Figure S11. Quantification of anchoring efficiency of compound 9 in the immunostaining experiments against microtubules 21
15. Figure S12. Variable temperature ^1H NMR spectra (600 MHz, DMSO-d$_6$) of compound S8 22
16. Figure S13. Partial variable temperature ^1H NMR spectra (600 MHz, DMSO-d$_6$) of compound 4 22
17. Scheme S1. Chemical structures of TRITON-2 and NPA-TRITON-2 23
18. Copies of ^1H and ^{13}C NMR spectra 24
19. References 48

1. Multifunctional molecule synthesis

1.1 General

All chemicals were purchased from Sigma-Aldrich, Fluorochem, TCI or ACROS and were used as received unless otherwise stated. Dry solvents were purchased from commercial sources and used directly. All reactions were monitored using thin layer chromatography (TLC) with silica gel plates (Kieselgel 60 F254 plates, Merck) under UV light. Column chromatography was performed using silica 60 (70-230 mesh) from E. M. Merck. Reverse phase column chromatography was performed using C18-reversed phase silica, fully end capped, 230-400 mesh, 90 Å pore size. Mass spectra were achieved using a Shimadzu LC-MS 2020 Liquid Chromatograph Mass Spectrometer (Shim-pack GIST C18 2 μm, 2.1x100 mm). ^1H NMR and ^{13}C NMR spectra were recorded using a Bruker Avance 400 MHz or a Bruker Avance Ⅱ$^+$ 600 MHz spectrometer in DMSO-d_6, MeOD-d_4 or $CDCl_3$ solution. Variable temperature NMR was performed to rotamer characterization.

1.2 Synthetic procedures

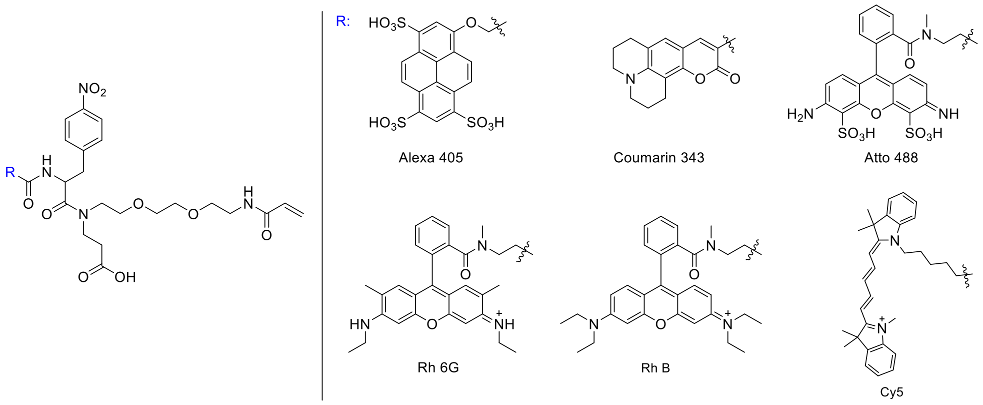

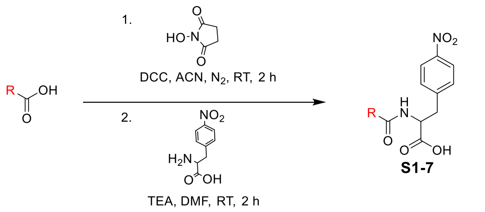

Synthesis of compound S1

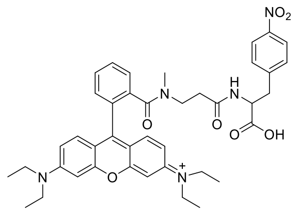

To a solution of rhodamine B (0.460 g, 0.817 mmol) in DMF (3 mL), DCC (0.370 g, 1.793 mmol) and N-Hydroxysuccinimide (0.103 g, 0.899 mmol) were added. The reaction was stirred at room temperature under the protection of N_2 for 2 h. After complete reaction, DCU was removed by filtration and the filtrate was evaporated under reduced pressure. The residue was further triturated with EtOAc (5 mL) in an ice bath. The mixture was filtered again to remove the precipitate (DCU). The filtrate was evaporated under reduced pressure and the residue was used for coupling without further purification.
To a solution of the intermediate in DMF (3 mL), 4-nitro-L-phenylalanine (0.171 g, 0.817 mmol) and triethylamine (0.227 mL, 1.634 mmol) were added. The reaction was stirred at room temperature under the protection of N_2 for 2 h. After complete reaction, all solvents were removed under reduced pressure and the residue was purified by column chromatography to yield the product as a red solid (395 mg, 63%). LC-MS (ESI$^+$): calculated for $C_{41}H_{46}N_5O_7$ [M]$^+$ m/z: 720.34; found: 720.15; mixture of rotamers; ^{1}H NMR (400 MHz, MeOD-*d*$_4$) δ 8.06 (d, *J* = 8.7 Hz, 2H), 7.80 – 7.71 (m, 2H), 7.67 – 7.56 (m, 1H), 7.54 – 7.46 (m, 1H), 7.43 – 7.38 (m, 2H), 7.29 – 7.26 (m, 2H), 7.12 – 7.01 (m, 2H), 6.97 – 6.95 (m, 2H), 4.59 – 4.45 (m, 1H), 3.75 – 3.64 (m, 8H), 3.43 – 3.33 (m, 3H), 3.26 (dd, *J* = 13.6, 6.1 Hz, 1H), 3.03 (dd, *J* = 13.5, 7.1 Hz, 1H), 2.82 (s, 2H), 2.63 (s, 1H), 1.99 (t, *J* = 7.0 Hz, 1H), 1.31 (t, *J* = 7.1 Hz, 12H); ^{13}C NMR (151 MHz, MeOD-*d*$_4$) δ 176.63, 172.11, 170.69, 159.45, 157.31, 157.04, 148.13, 137.68, 137.21, 133.41, 133.26, 131.94, 131.85, 131.46, 131.01, 129.02, 124.36, 124.23, 115.31, 114.84, 97.55, 56.88,47.03, 45.46, 39.35, 38.83, 35.67, 34.24, 32.58, 14.99.
Other analogues were synthesized and purified based on the same method as described above, unless noted otherwise.

Synthesis of compound S2

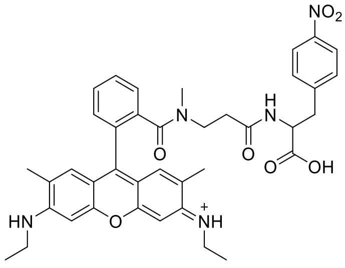

Orange solid; Yield: 55% (282 mg); LC-MS (ESI$^+$): calculated for $C_{39}H_{42}N_5O_7$ [M]$^+$ m/z: 692.31; found: 692.10; mixture of rotamers; ^{1}H NMR (400 MHz, MeOD-*d*$_4$) δ 8.06 (t, *J* = 8.6 Hz, 2H), 7.80 – 7.73 (m, 2H), 7.68 – 7.45 (m, 2H), 7.44 – 7.38 (m, 2H), 7.01 (brs, 2H), 6.92 (d, *J* = 3.0 Hz, 2H), 4.57 – 4.45 (m, 1H), 3.54 (p, *J* = 7.2 Hz, 4H), 3.26 (td, *J* = 10.6, 5.0 Hz, 1H), 3.05 – 2.96 (m, 1H), 2.85 (s, 2H), 2.60 (s, 1H), 2.18 (s, 5H), 2.11 (s, 1H), 1.97 – 1.87 (m, 4H), 1.37 (t, *J* = 7.1 Hz, 6H); ^{13}C NMR (151 MHz, MeOD-*d*$_4$) δ 172.16, 170.73, 159.10, 157.91, 148.19, 137.63, 132.35, 131.83, 131.33, 131.05, 130.88, 129.04, 126.72, 124.24, 115.00, 95.12, 57.62, 56.97, 45.43, 39.63, 38.88, 34.18, 30.91, 26.26, 23.85, 23.30, 17.66, 17.43, 14.21.

Synthesis of compound S3

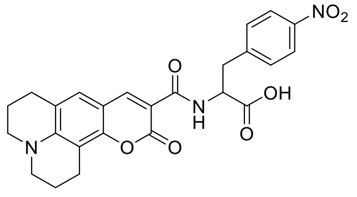

Pale green solid; Yield: 58% (235 mg); LC-MS (ESI$^+$): calculated for $C_{25}H_{24}N_3O_7$ [M+H]$^+$ m/z: 478.16; found: 477.95; ^{1}H NMR (400 MHz, DMSO-*d*$_6$) δ 9.03 (d, *J* = 6.2 Hz, 1H), 8.47 (s, 1H), 8.06 (d, *J* = 7.5 Hz, 2H), 7.42 (d, *J* = 7.5 Hz, 2H), 7.21 (s, 1H), 4.67 – 4.56 (m, 1H), 3.58 – 3.39 (m, 4H), 3.24 – 3.16 (m, 2H), 2.75 – 2.65 (m, 4H), 1.90 – 1.82 (m, 4H); ^{13}C NMR (101 MHz, DMSO-*d*$_6$) δ 161.68, 152.07, 147.99, 147.51, 146.07, 130.72, 127.12, 122.95, 119.37, 107.31, 104.46, 54.35, 49.52, 48.99, 37.28, 26.28, 22.10, 20.51, 19.56.

Synthesis of compound S4

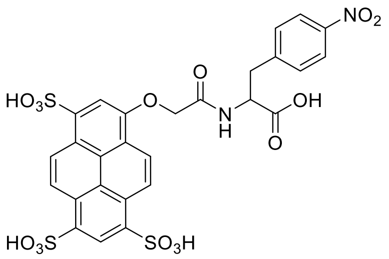

The crude product was purified by reverse-phase column chromatography. Pale yellow solid; Yield: 61% (53 mg); LC-MS (ESI$^+$): calculated for $C_{27}H_{18}N_2O_{15}S_3$ [M-2H]$^{2-}$ m/z: 353.00; found: 352.90; ^{1}H NMR (400 MHz, MeOD-*d*$_4$) δ 9.38 (s, 1H), 9.23 (dd, *J* = 14.4, 5.9 Hz, 2H), 9.14 (d, *J* = 9.8 Hz, 1H), 8.73 (dd, *J* = 9.6, 3.1 Hz, 1H), 8.33 (d, *J* = 5.1 Hz, 1H), 8.10 – 8.01 (m, 2H), 7.45 (dd, *J* = 8.4, 6.3 Hz, 2H), 4.97 (brs, 2H), 4.91 – 4.89 (m, 1H), 3.40 (td, *J* = 13.5, 5.3 Hz, 1H), 3.26 – 3.16 (m, 1H), including peaks from triethylamine hydrochloride; ^{13}C NMR (151 MHz, MeOD-*d*$_4$) δ 172.85, 170.75, 153.31, 148.63, 146.58, 131.79, 131.45, 131.01, 128.78, 127.36, 126.74, 125.50, 124.84, 124.02, 123.45, 123.01, 111.31, 69.45, 54.82, 38.27.

Synthesis of compound S5

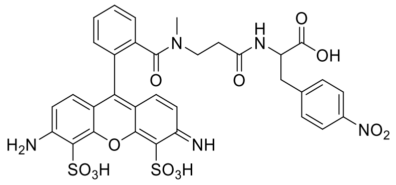

The crude product was purified by reverse-phase column chromatography. Yellow solid; Yield: 25% (30 mg); LC-MS (ESI$^+$): calculated for $C_{33}H_{28}N_5O_{13}S_2$ [M-H]$^-$ m/z: 766.11; found: 765.95; mixture of rotamers; ^{1}H NMR (400 MHz, D_2O) δ 8.22 – 8.07 (m, 2H), 7.82 – 7.69 (m, 2H), 7.58 – 7.38 (m, 4H), 7.27 (d, *J* = 9.2 Hz, 1H), 7.17 (d, *J* = 8.6 Hz, 1H), 7.09 – 6.86 (m, 2H), 3.50 – 3.29 (m, 2H), 3.28 – 3.15 (m, 2H), 3.14 – (m, 1H), 2.62 (s, 2H), 2.46 (s, 1H), 2.40 – 2.22 (m, 1H).

Synthesis of compound S6

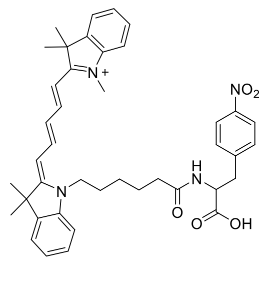

Blue solid; Yield: 54% (353 mg); LC-MS (ESI$^+$): calculated for $C_{41}H_{47}N_4O_5$ [M]$^+$ m/z: 675.35; found: 675.20; ^{1}H NMR (400 MHz, MeOD-*d*$_4$) δ 8.20 (t, *J* = 12.8 Hz, 2H), 8.13 (dd, *J* = 7.6, 6.5 Hz, 2H), 7.45 (d, *J* = 8.6 Hz, 5H), 7.38 (dd, *J* = 13.7, 7.4 Hz, 2H), 7.33 – 7.15 (m, 4H), 6.59 (t, *J* = 12.4 Hz, 2H), 6.22 (dd, *J* = 13.7, 8.6 Hz, 1H), 4.74 (dt, *J* = 9.1, 4.6 Hz, 1H), 4.01 (t, *J* = 7.5 Hz, 2H), 3.60 (s, 3H), 3.38 – 3.31 (m, 1H), 3.05 (dt, *J* = 13.9, 4.6 Hz, 1H), 2.21 – 2.11 (m, 2H), 1.69 (s, 12H), 1.61 – 1.54 (m, 2H), 1.41 – 1.25 (m, 4H); ^{13}C NMR (101 MHz, MeOD-*d*$_4$) δ 175.58, 174.35, 155.53, 148.42, 146.83, 144.33, 143.59, 142.65, 131.56, 129.83, 126.66, 126.32, 124.52, 123.42, 111.98, 104.29, 54.46, 50.57, 44.81, 38.34, 36.31, 31.58, 28.00, 27.23, 26.31, 22.37.

Synthesis of compound S7

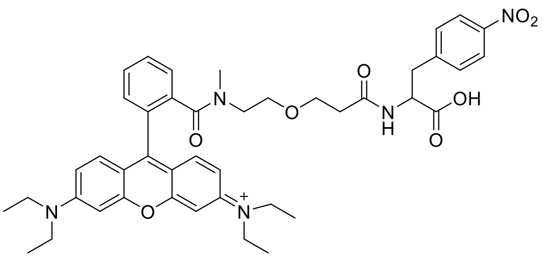

To a solution of the modified rhodamine B[1] (100 mg, 0.175 mmol) in DMF (1.5 mL), DIC (68 µL, 0.437 mmol), triethylamine (24 µL, 0.175 mmol) and N-Hydroxysuccinimide (24 mg, 0.210 mmol) were added. The reaction was stirred at room temperature under the protection of N_2 for 2 h. After complete reaction, the white precipitate was removed by filtration and the filtrate was evaporated under reduced pressure. The residue was further triturated with EtOAc (5 mL) in an ice bath. The mixture was filtered again to remove the precipitate. The filtrate was evaporated under reduced pressure and the residue was used for coupling without further purification.
To a solution of the intermediate in DMF (3 mL), 4-nitro-L-phenylalanine (73 mg, 0.349 mmol) and triethylamine (48 µL, 0.350 mmol) were added. The reaction was stirred at room temperature under the protection of N_2 for 2 h. After complete reaction, all solvents were removed under reduced pressure and the residue was purified by column chromatography to yield the product as a red solid (60 mg, 45%). LC-MS (ESI$^+$): calculated for $C_{43}H_{50}N_5O_8$ [M]$^+$ m/z: 764.37; found: 764.10; mixture of rotamers; ^{1}H NMR (400 MHz, MeOD-d_4) δ 8.05 (t, *J* = 7.6 Hz, 2H), 7.80 – 7.70 (m, 2H), 7.70 – 7.63 (m, 1H), 7.52 – 7.47 (m, 1H), 7.45 – 7.39 (m, 2H), 7.31 – 7.24 (m, 2H), 7.07 (dt, *J* = 9.5, 2.3 Hz, 2H), 7.00 – 6.92 (m, 2H), 4.58 – 4.48 (m, 1H), 3.77 – 3.64 (m, 8H), 3.36 -3.34 (m, 3H), 3.31 – 3.23 (m, 1H), 3.22 – 3.02 (m, 3H), 2.80 (s, 2H), 2.65 (s, 1H), 2.27 (t, J = 6.1 Hz, 1H), 1.31 (dd, *J* = 8.5, 4.8 Hz, 12H); ^{13}C NMR (101 MHz, MeOD-d_4) δ 176.68, 172.73, 170.69, 159.36, 157.30, 157.00, 148.08, 137.72, 133.45, 131.70, 131.64, 130.92, 128.91, 124.16, 115.33, 114.87, 97.43, 69.70, 68.05, 56.85, 52.16, 39.95, 39.17, 37.73, 32.81, 28.21, 17.44, 12.94.

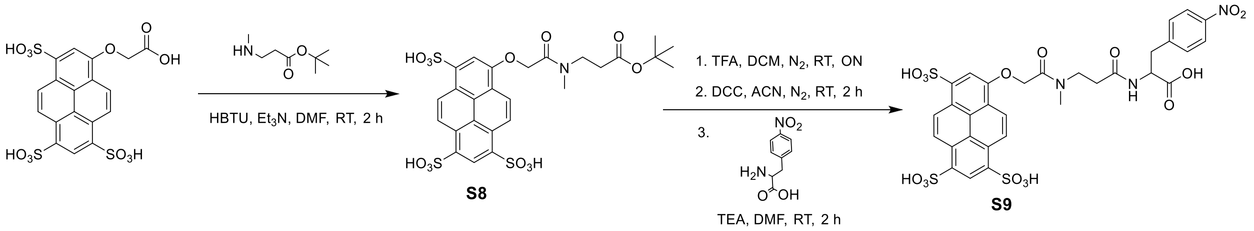

Synthesis of compound S8

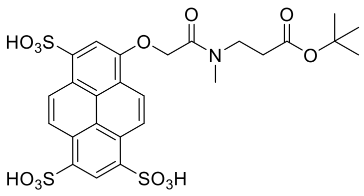

To a solution of Alexa 405 (30 mg, 51.5 µmol) in DMF (1 mL), triethylamine (25 µL, 180 µmol) and HBTU (23 mg, 60.7 µmol) were added. After 10 minutes, tert-butyl 3-(methylamino)propanoate (12 mg, 75.4 µmol) was added and the reaction mixture was stirred at room temperature for 2 h. After complete reaction, all solvents were removed under reduced pressure and the residue was purified by column chromatography to yield the product as a pale yellow solid (26 mg, 68%). LC-MS (ESI$^+$): calculated for $C_{26}H_{25}NO_{13}S_3$ [M-2H]$^{2-}$ m/z: 327.52; found: 327.40; mixture of rotamers; ^{1}H NMR (400 MHz, MeOD-d_4) δ 9.42 (s, 1H), 9.26 (t, *J* = 9.2 Hz, 2H), 9.16 (d, *J* = 9.9 Hz, 1H), 8.78 (dd, *J* = 9.7, 5.7 Hz, 1H), 8.36 (d, *J* = 11.3 Hz, 1H), 5.33 (d, *J* = 48.4 Hz, 2H), 3.83 (t, *J* = 6.8 Hz, 1H), 3.73 (t, *J* = 7.1 Hz, 1H), 3.26 (s, 2H), 3.03 (s, 1H), 2.72 (t, *J* = 6.8 Hz, 1H), 2.61 (t, *J* = 7.1 Hz, 1H), 1.45 (d, *J* = 11.9 Hz, 9H).

Synthesis of compound S9

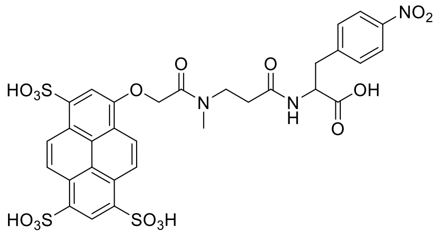

To a solution of the intermediate S8 in DCM (0.2 mL), TFA (0.2 mL) was added and the reaction mixture was stirred overnight at room temperature. After complete reaction, all solvents were evaporated to yield the intermediate as a red solid, which was used without further purification.

Compound **S9** was synthesized and purified based on the same method as described in compound **S1**.

Yellow solid; Yield: 41% (10 mg); LC-MS (ESI$^+$): calculated for $C_{31}H_{25}N_3O_{16}S_3$ [M-2H]$^{2-}$ m/z: 395.52; found: 395.45; mixture of rotamers; ^{1}H NMR (400 MHz, MeOD-*d$_4$*) δ 9.40 (s, 1H), 9.30 – 9.22 (m, 2H), 9.15 (d, *J* = 9.8 Hz, 1H), 8.77 (dd, *J* = 9.6, 2.8 Hz, 1H), 8.26 (d, *J* = 11.6 Hz, 1H), 8.12 (ddd, *J* = 8.7, 4.6, 2.3 Hz, 2H), 7.56 – 7.45 (m, 2H), 5.37 – 5.18 (m, 2H), 4.82 – 4.75 (m, 1H), 3.79 – 3.71 (m, 1H), 3.65 – 3.57 (m, 1H), 3.29 – 3.24 (m, 1H), 3.23 – 3.13 (m, 1H), 3.10 (s, 2H), 2.87 (s, 1H), 2.71 – 2.58 (m, 1H), 2.48 – 2.41 (m, 1H), including peaks from triethylamine hydrochloride; ^{13}C NMR (151 MHz, MeOD-*d$_4$*) δ 172.56, 171.68, 171.47, 168.43, 168.20, 152.28, 152.22, 146.94, 145.29, 145.07, 140.72, 137.06, 130.14, 129.80, 129.31, 127.07, 125.77, 125.70, 125.51, 124.99, 123.59, 123.15, 123.07, 122.48, 121.32, 121.20, 110.07, 109.51, 66.84, 66.38, 53.43, 53.37, 48.46, 45.10, 44.69, 36.52, 36.36, 34.44, 34.23, 33.72, 32.44.

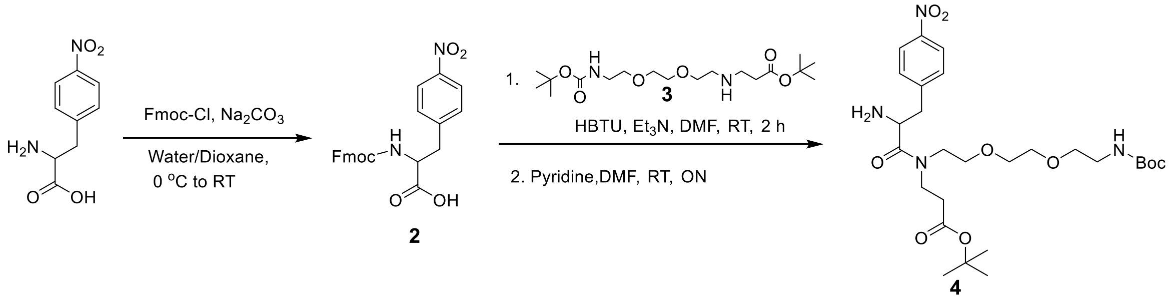

Synthesis of compound 2

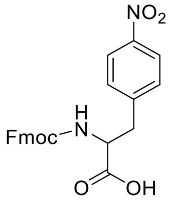

To a solution of 4-nitro-L-phenylalanine (1.50 g, 7.14 mmol) in dioxane (7.5 mL), 10% Na_2CO_3 aqueous solution (15 mL) was added, followed by a solution of Fmoc chloride (2.52 g, 9.74 mmol) in dioxane (7.5 mL) at 0 °C with an ice bath. The mixture was stirred at room temperature overnight. After complete reaction, all solvents were removed under reduced pressure and the residue was dissolved with water (15 mL). The pH was then adjusted to 2 by addition of conc. HCl and the precipitate was collected by filtration. Afterwards, the solid was dissolved in EtOAc (300 mL) under heating and the mixture was washed with water (300 mL, 2 x). The organic phase was dried over $MgSO_4$ and evaporated under reduced pressure. The residue was purified by washing with a minimal amount of dichloromethane and the solid was dried by high vacuum to yield the product as a yellow solid (2.47 g, 80%). LC-MS (ESI$^+$): calculated for $C_{24}H_{19}N_2O_6$ [M-H]$^-$ m/z: 431.12; found: 430.85; ^{1}H NMR (400 MHz, DMSO-*d$_6$*) δ 8.14 – 8.07 (m, 2H), 7.87 (d, *J* = 7.5 Hz, 2H), 7.65 – 7.56 (m, 2H), 7.50 (d, *J* = 8.6 Hz, 2H), 7.40 (t, *J* = 7.4 Hz, 2H), 7.33 – 7.25 (m, 2H), 4.26 – 4.19 (m, 2H), 4.15 (dd, *J* = 12.6, 5.8 Hz, 2H), 3.23 (dd, *J* = 13.6, 4.5 Hz, 1H), 3.02 (dd, *J* = 13.5, 9.7 Hz, 1H).

Synthesis of compound 4

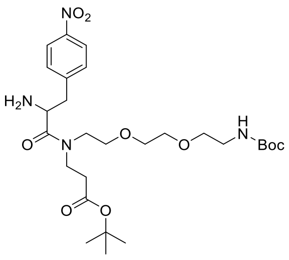

To a solution of the compound 2 (0.949 g, 2.196 mmol) in DMF (5 mL), triethylamine (20.916 mL, 6.590 mmol) and HBTU (0.916 g, 2.416 mmol) were added. After 10 minutes, the amine diPEG linker **3** (0.992 g, 2.635 mmol, synthesized according to literature[2]) was added and the reaction mixture was stirred at room temperature for 2 h. After complete reaction, pyridine (3 mL) was added to the reaction mixture followed by stirring at room temperature overnight to remove the Fmoc protection group. After complete reaction, as indicated by TLC, 200 mL water was added into the reaction flask, followed by extraction with ethyl acetate (150 mL, 3 x). The combined organic phases were further washed with brine (200 mL). And then it was dried over $MgSO_4$ and evaporated to dryness. The residue was purified by column chromatography to obtain the product as a yellow solid (0.686 g, 55%). LC-MS (ESI$^+$): calculated for $C_{27}H_{45}N_4O_9$ $[M+H]^+$ m/z: 569.32; found: 569.10; mixture of rotamers; ^{1}H NMR (400 MHz, MeOD-d_4) δ 8.21 – 8.18 (m, 2H), 7.52 (dd, J = 8.6, 6.6 Hz, 2H), 4.22 (ddd, J = 21.0, 8.0, 6.2 Hz, 1H), 3.77 – 3.66 (m, 1H), 3.65 – 3.50 (m, 8H), 3.47 (t, J = 6.2 Hz, 2H), 3.32 – 3.29 (m, 1H), 3.27 – 3.19 (m, 2H), 3.16 – 3.06 (m, 1H), 3.02 (dt, J = 13.0, 6.4 Hz, 1H), 2.57 – 2.26 (m, 2H), 1.47 – 1.41 (m, 18H); ^{13}C NMR (101 MHz, MeOD-d_4) δ 176.66, 175.92, 172.81, 172.38, 148.45, 146.89, 146.74, 131.82, 124.66, 82.33, 82.06, 80.23, 71.56, 71.49, 71.36, 71.24, 71.08, 70.11, 69.86, 53.12, 52.68, 47.46, 45.57, 44.07 42.62, 41.73, 41.30, 35.53, 34.71, 28.88, 28.42.

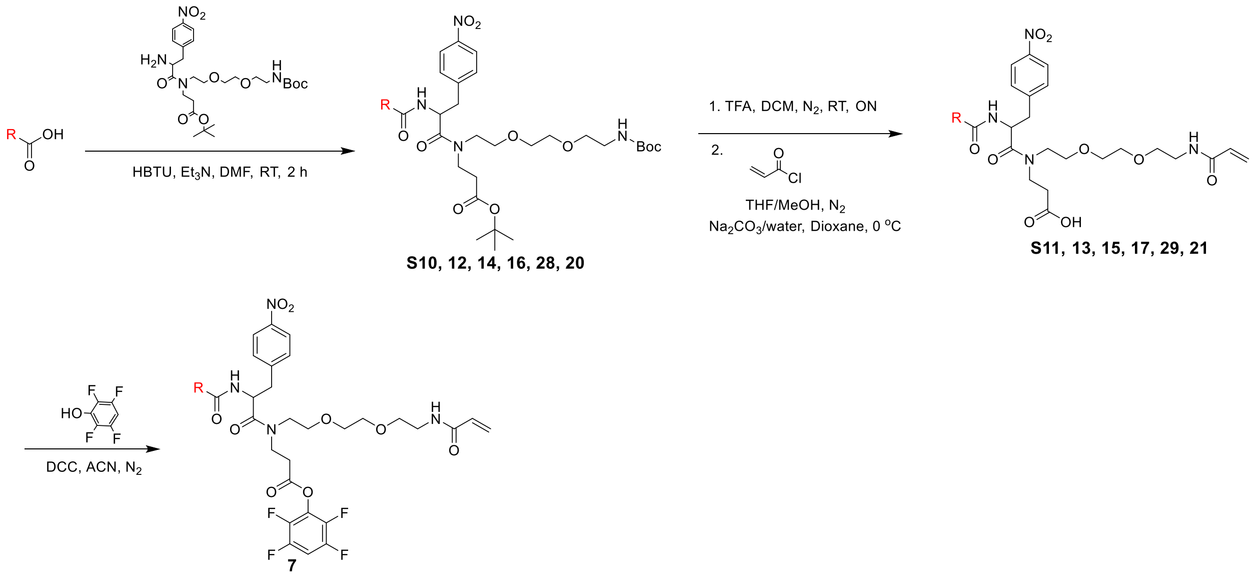

Synthesis of compound S10

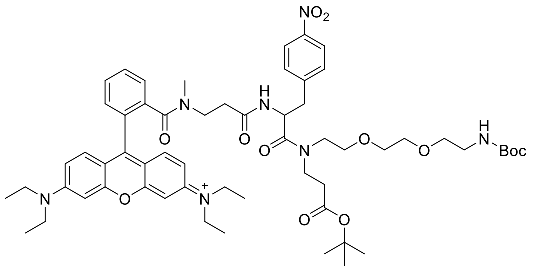

To a solution of Rh B (84 mg, 149 μmol) in DMF (3 mL), triethylamine (62 μL, 446 μmol) and HBTU (62 mg, 164 μmol) were added. After 10 minutes, the linker 4 (93 mg, 164 μmol) was added and the reaction mixture was stirred at room temperature for 2 h. After complete reaction, 30 mL ethyl acetate was added into the reaction flask, followed by washing with water (30 mL, 2 x) and brine (30 mL). The organic layer was dried over $MgSO_4$ and then evaporated under reduced pressure. The residue was purified by column chromatography to yield the intermediate as a red solid (117 mg, 68%). LC-MS (ESI$^+$): calculated for $C_{59}H_{80}N_7O_{12}$ $[M]^+$ m/z: 1078.59;

found: 1078.40; mixture of rotamers; ^{1}H NMR (600 MHz, MeOD-d_4) δ 8.14 (dd, *J* = 10.6, 8.7 Hz, 2H), 7.80 – 7.76 (m, 2H), 7.73 – 7.62 (m, 1H), 7.56 – 7.45 (m, 3H), 7.35 – 7.24 (m, 2H), 7.16 – 7.05 (m, 2H), 6.99 (brs, 2H), 5.25 – 5.07 (m, 1H), 3.76 – 3.68 (m, 11H), 3.66 – 3.53 (m, 8H), 3.51 (dd, *J* = 11.5, 5.8 Hz, 1H), 3.47 – 3.39 (m, 2H), 3.28 (t, *J* = 7.2 Hz, 1H), 3.23 (dd, *J* = 11.1, 5.5 Hz, 1H), 3.17 (t, *J* = 5.8 Hz, 1H), 3.03 – 2.95 (m, 1H), 2.88 (s, 2H), 2.61 (s, 1H), 2.56 – 2.43 (m, 2H), 2.18 – 2.05 (m, 2H), 1.49 – 1.41 (m, 18H), 1.34 (t, *J* = 7.1 Hz, 12H).
Other analogues were synthesized and purified based on the same method as described above, unless stated otherwise.

Synthesis of compound S11

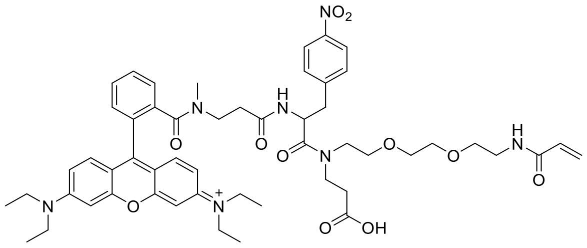

To a solution of the intermediate S10 in DCM (0.2 mL), TFA (0.2 mL) was added and the reaction mixture was stirred overnight at room temperature. After complete reaction, all solvents were evaporated to yield the intermediate as a red solid, which was used without further purification.
To a solution of the intermediate (110 mg, 115 μmol) in MeOH: THF (*v*:*v* = 0.5 mL: 0.5 mL), a solution of Na_2CO_3 (24.4 mg, 230 μmol) in water (0.5 mL) was added under the protection of N_2, followed by cooling the reaction flask to 0 °C with an ice bath. A solution of acryloyl chloride (12.1 μL, 150 μmol) in dry dioxane (0.3 mL) was added dropwise to the reaction flask, and the reaction mixture was then allowed to come to room temperature over 20 min. After complete reaction, all solvents were evaporated and the residue was purified by column chromatography to yield the product as a red solid (59 mg, 57%). LC-MS (ESI$^+$): calculated for $C_{53}H_{66}N_7O_{11}$ $[M]^+$ m/z: 976.48; found: 976.25; mixture of rotamers; ^{1}H NMR (400 MHz, MeOD-d_4) δ 8.12 (d, *J* = 8.5 Hz, 2H), 7.81 – 7.71 (m, 2H), 7.65 (dd, *J* = 5.9, 2.8 Hz, 1H), 7.57 – 7.46 (m, 3H), 7.30 – 7.24 (m, 2H), 7.08 (dt, *J* = 10.2, 5.1 Hz, 2H), 6.97 (t, *J* = 3.8 Hz, 2H), 6.36 – 6.16 (m, 2H), 5.63 (ddd, *J* = 9.7, 5.3, 2.2 Hz, 1H), 5.34 – 5.15 (m, 1H), 3.77 – 3.67 (m, 8H), 3.67 – 3.50 (m, 10H), 3.50 – 3.41 (m, 3H), 3.41 – 3.35 (m, 2H), 3.29 – 3.18 (m, 2H), 2.98 (dt, *J* = 14.1, 8.7 Hz, 1H), 2.85 (s, 2H), 2.60 (d, *J* = 6.9 Hz, 1H), 2.49 – 2.38 (m, 2H), 2.12 – 1.97 (m, 2H), 1.32 (t, *J* = 7.1 Hz, 12H); ^{13}C NMR (101 MHz, MeOD-d_4) δ 187.76, 180.01, 172.76, 170.64, 168.28, 159.37, 157.28, 148.31, 147.08, 137.40, 133.25, 132.18, 131.84, 131.74, 131.34, 131.07, 129.05, 126.73, 124.51, 115.32, 97.42, 71,60, 70.58, 69.93, 45.56, 40.60, 39.97, 36.89, 34.04, 30.84, 23.83, 14.60, 12.95.
Other analogues were synthesized and purified based on the same method as described above, unless noted otherwise.

Synthesis of compound 7

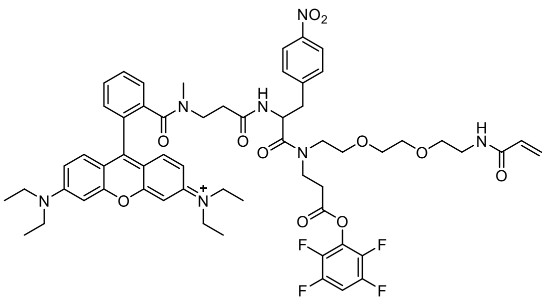

To a solution of the intermediate S11 (30 mg, 30 μmol) in DMF (1 mL), DIC (9.3 μL, 59 μmol) and 2,3,5,6-Tetrafluorophenol (6.8 μL, 59 μmol) were added. The reaction mixture was stirred at room temperature for 2 h. After complete reaction, the mixture was filtered to remove the white precipitate and the filtrate was evaporated under reduced pressure. The residue was further triturated with acetonitrile (2 mL) in an ice bath. The mixture was filtered again to remove the precipitate. The filtrate was evaporated under reduced pressure and the residue was used for coupling without further purification. LC-MS (ESI$^+$): calculated for $C_{59}H_{66}F_4N_7O_{11}$ $[M]^+$ m/z: 1124.48; found: 1124.30.

Synthesis of compound S12

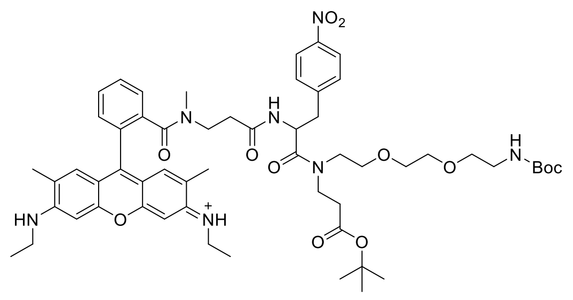

Orange solid; Yield: 61% (70 mg); LC-MS (ESI$^+$): calculated for $C_{57}H_{76}N_7O_{12}$ $[M]^+$ m/z: 1050.55; found: 1050.30; mixture of rotamers; ^{1}H NMR (400 MHz, MeOD-d_4) δ 8.15 – 8.09 (m, 2H), 7.77 (dd, *J* = 5.6, 3.2 Hz, 2H), 7.68 – 7.63 (m, 1H), 7.55 – 7.44 (m, 3H), 7.10 – 6.95 (m, 2H), 6.94 – 6.84 (m, 2H), 5.30 – 5.12 (m, 1H), 3.76 – 3.67 (m, 3H), 3.66 – 3.51 (m, 13H), 3.45 – 3.39 (m, 2H), 3.30 – 3.22 (m, 3H), 3.21 – 3.12 (m, 2H), 3.10 – 2.93 (m, 1H), 2.87 (d, *J* = 13.3 Hz, 2H), 2.57 (d, *J* = 11.9 Hz, 1H), 2.55 – 2.39 (m, 2H), 2.19 (s, 5H), 2.12 (s, 1H), 1.45 (dd, *J* = 7.9, 3.6 Hz, 18H), 1.38 (t, *J* = 7.2 Hz, 6H).

Synthesis of compound S13

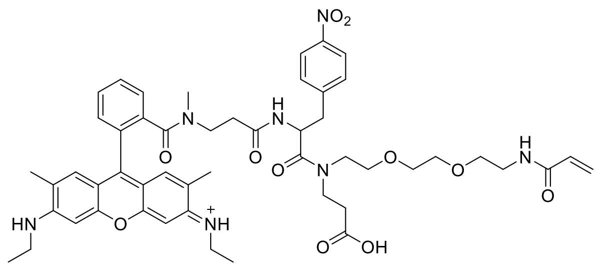

Orange solid; Yield: 50% (42 mg); LC-MS (ESI$^+$): calculated for $C_{51}H_{62}N_7O_{11}$ $[M]^+$ m/z: 948.45; found: 948.20; mixture of rotamers; ^{1}H NMR (400 MHz, MeOD-d_4) δ 8.11 – 8.08 (m, 2H), 7.80 – 7.69 (m, 2H), 7.65 – 7.41 (m, 4H), 7.02 (d, *J* = 2.6 Hz, 2H), 6.88 (d, *J* = 5.1 Hz, 2H), 6.27 – 6.14 (m, 2H), 5.62 (ddd, *J* = 8.7, 5.0, 2.4 Hz, 1H), 5.32 – 5.07 (m, 1H), 3.65 – 3.48 (m, 16H), 3.46 – 3.41 (m, 2H), 3.39 – 3.33 (m, 3H), 3.29 – 3.22 (m, 2H), 3.02 – 2.92 (m, 1H), 2.86 (s, 2H), 2.62 – 2.49 (m, 3H), 2.16 (s, 5H), 2.09 (s, 1H), 1.37 – 1.27 (m, 9H, including peaks from triethylamine hydrochloride); ^{13}C NMR (101 MHz, MeOD-d_4) δ 173.88, 171.77, 169.08, 166.78, 157.47, 156.35, 155.05, 146.85, 145.34, 135.85, 130.94, 130.61, 130.32, 129.69, 127.51, 125.27, 123.04, 113.42, 93.50, 70.10, 69.07, 68.63, 50.93, 44.00, 43.08, 39.01, 38.07, 37.34, 32.79, 32.38, 31.66, 31.03, 29.32, 28.35, 26.10, 16.13, 12.66.

Synthesis of compound S14

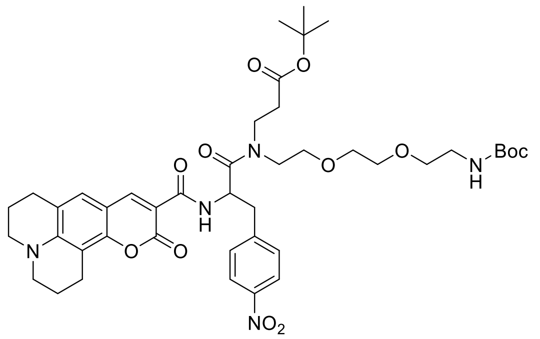

Pale green solid; Yield: 45% (83 mg); LC-MS (ESI$^+$): calculated for $C_{43}H_{58}N_5O_{12}$ $[M+H]^+$ m/z: 836.41; found: 836.20; mixture of rotamers; ^{1}H NMR (400 MHz, MeOD-d_4) δ 8.39 (d, *J* = 7.5 Hz, 1H), 8.16 (dd, *J* = 8.6, 6.4 Hz, 2H), 7.55 (dd, *J* = 14.2, 8.7 Hz, 2H), 7.05 (d, *J* = 4.0 Hz, 1H), 5.44 (dt, *J* = 17.3, 7.1 Hz, 1H), 3.79 – 3.65 (m, 2H), 3.64 – 3.52 (m, 7H), 3.50 – 3.44 (m, 1H), 3.44 – 3.36 (m, 6H), 3.32 – 3.29 (m, 1H), 3.20 (t, *J* = 5.6 Hz, 1H), 3.15 (t, *J* = 5.7 Hz, 2H), 2.81 (t, *J* = 6.3 Hz, 2H), 2.75 (t, *J* = 5.9 Hz, 2H), 2.66 – 2.43 (m, 2H), 2.01 – 1.90 (m, 4H), 1.43 (dd, *J* = 9.0, 4.0 Hz, 18H).

Synthesis of compound S15

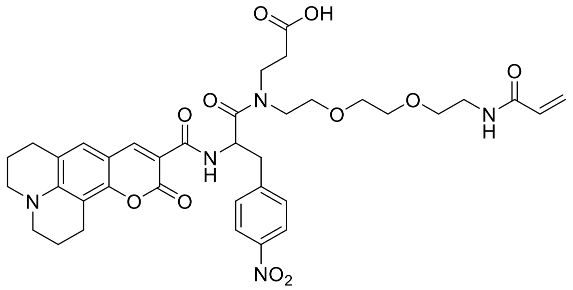

Pale green solid; Yield: 31% (33 mg); LC-MS (ESI+): calculated for $C_{37}H_{44}N_5O_{11}$ $[M+H]^+$ m/z: 734.30; found: 734.10; mixture of rotamers; ^{1}H NMR (400 MHz, MeOD-*d*$_4$) δ 8.33 (d, *J* = 5.6 Hz, 1H), 8.14 (ddd, *J* = 9.0, 4.6, 1.9 Hz, 2H), 7.52 (dt, *J* = 8.3, 7.3 Hz, 2H), 6.99 (t, *J* = 3.1 Hz, 1H), 6.35 – 6.06 (m, 2H), 5.63 (ddd, *J* = 9.7, 2.5, 1.1 Hz, 1H), 5.54 – 5.36 (m, 1H), 3.82 – 3.68 (m, 2H), 3.68 – 3.50 (m, 8H), 3.50 – 3.39 (m, 3H), 3.36 (dd, *J* = 6.3, 5.1 Hz, 5H), 3.30 – 3.27 (m, 1H), 3.23 – 3.11 (m, 1H), 2.77 – 2.69 (m, 4H), 2.63 (ddd, *J* = 19.0, 12.4, 5.6 Hz, 2H), 1.98 – 1.90 (m, 4H); ^{13}C NMR (151 MHz, MeOD-*d*$_4$) δ 175.43, 174.77, 173.31, 172.94, 168.25, 164.87, 164.24, 154.11, 150.37, 149.35, 148.52, 146.26, 131.98, 131.83, 128.60, 126.80, 124.60, 121.81, 109.39, 107.75, 106.55, 71.77, 71.52, 71.44, 71.34, 70.58, 69.96, 51.91, 51.37, 50.87, 50.03, 47.77, 46.08, 44.61, 40.52, 39.82, 34.31, 33.25, 28.46, 22.18, 21.20, 20.03.

Synthesis of compound S16

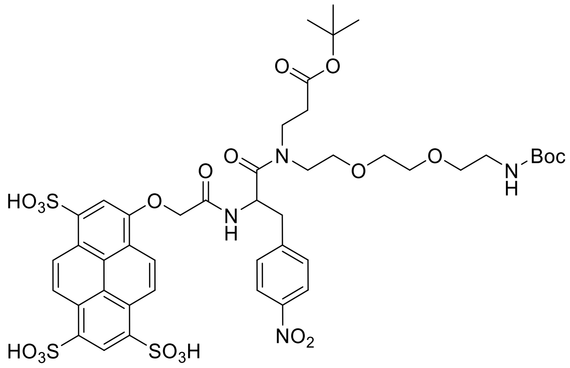

Pale yellow solid; Yield: 55% (37 mg); LC-MS (ESI+): calculated for $C_{45}H_{55}N_4O_{20}S_3$ $[M+H]^+$ m/z: 1067.26; found: 1067.20; ^{1}H NMR (400 MHz, MeOD-*d*$_4$) δ 9.44 (s, 1H), 9.31 (dd, *J* = 9.8, 1.9 Hz, 2H), 9.20 (d, *J* = 9.9 Hz, 1H), 8.78 (dd, *J* = 7.9, 5.2 Hz, 1H), 8.37 (t, *J* = 8.8 Hz, 1H), 8.13 (dd, *J* = 8.4, 7.7 Hz, 2H), 7.55 (dd, *J* = 12.4, 8.7 Hz, 2H), 5.48 – 5.31 (m, 1H), 5.20 – 4.95 (m, 2H), 3.86 – 3.79 (m, 1H), 3.67 – 3.51 (m, 6H), 3.50 – 3.36 (m, 5H), 3.32 – 3.30 (m, 1H), 3.26 – 3.15 (m, 3H), 2.65 – 2.43 (m, 2H), 1.47 – 1.38 (m, 18H), including peaks from triethylamine hydrochloride.

Synthesis of compound S17

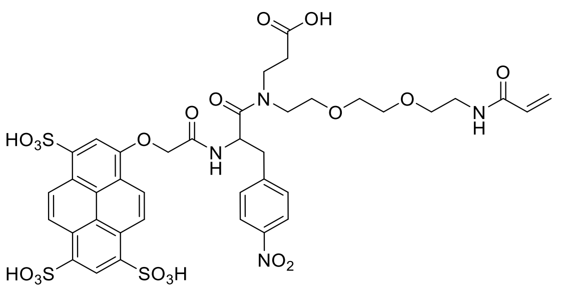

Pale yellow solid; Yield: 27% (15 mg); LC-MS (ESI+): calculated for $C_{39}H_{41}N_4O_{19}S_3$ $[M+H]^+$ m/z: 965.15; found: 964.90; mixture of rotamers; ^{1}H NMR (400 MHz, MeOD-*d*$_4$) δ 9.41 (s, 1H), 9.27 (dd, *J* = 9.7, 2.4 Hz, 2H), 9.16 (d, *J* = 9.9 Hz, 1H), 8.74 (dd, *J* = 9.6, 6.8 Hz, 1H), 8.34 (d, *J* = 4.3 Hz, 1H), 8.10 (t, *J* = 8.9 Hz, 2H), 7.52 (dd, *J* = 13.4, 8.7 Hz, 2H), 6.31 – 6.05 (m, 2H), 5.59 (ddd, *J* = 12.0, 9.9, 2.1 Hz, 1H), 5.46 – 5.33 (m, 1H), 4.98 (t, *J* = 3.6 Hz, 2H), 3.75 – 3.67 (m, 1H), 3.58 – 3.53 (m, 2H), 3.52 – 3.45 (m, 4H), 3.42 (dd, *J* = 11.2, 6.3 Hz, 3H), 3.38 – 3.33 (m, 3H), 3.29 – 3.24 (m, 1H), 3.18 – 3.10 (m, 2H), 2.71 – 2.55 (m, 1H), 2.49 – 2.39 (m, 1H), including peaks from triethylamine hydrochloride; ^{13}C NMR (101 MHz, MeOD-*d*$_4$) δ 173.99, 173.54, 172.79, 172.47, 169.87, 168.34, 152.91,

148.41, 146.53, 146.29, 131.91, 131.21, 130.79, 128.53, 127.30, 127.10, 126.57, 125.34, 124.57, 123.66, 123.26, 122.71, 110.93, 71.63, 71.38, 71.29, 70.38, 69.90, 69.54, 69.13, 54.14, 52.90, 51.59, 51.38, 47.40, 45.53, 44.26, 40.55, 39.12, 33.97, 32.87, 27.50.

Synthesis of compound S18

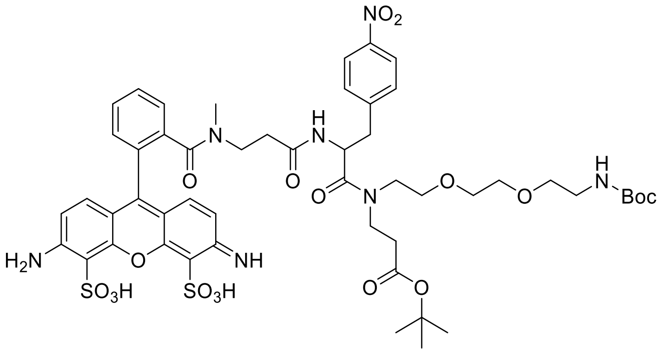

Yellow solid; Yield: 50%(17 mg); LC-MS (ESI$^+$): calculated for $C_{51}H_{64}N_7O_{18}S_2$ $[M+H]^+$ m/z: 1126.37; found: 1126.15; mixture of rotamers; ^{1}H NMR (600 MHz, MeOD-d_4) δ 8.20 – 8.08 (m, 2H), 7.81 – 7.71 (m, 2H), 7.66 – 7.44 (m, 4H), 7.26 (d, J = 9.3 Hz, 1H), 7.16 (dd, J = 9.2, 5.0 Hz, 1H), 6.97 (dd, J = 9.1, 5.2 Hz, 2H), 5.26 – 5.10 (m, 1H), 3.98 – 3.69 (m, 1H), 3.68 – 3.52 (m, 9H), 3.52 – 3.37 (m, 4H), 3.30 – 3.19 (m, 3H), 3.19 – 3.14 (m, 3H), 2.77 – 2.47 (m, 5H), 1.44 (dd, J = 7.9, 3.6 Hz, 18H).

Synthesis of compound S19

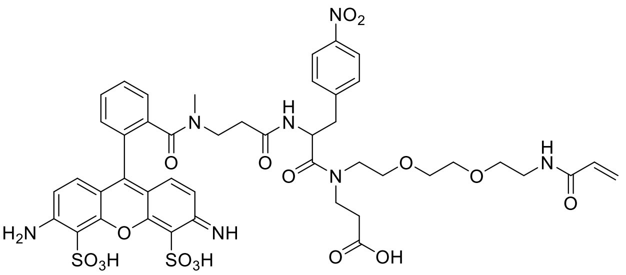

Yellow solid; Yield: 22% (3 mg); LC-MS (ESI$^+$): calculated for $C_{45}H_{50}N_7O_{17}S_2$ $[M+H]^+$ m/z: 1024.27; found: 1024.10; mixture of rotamers; ^{1}H NMR (600 MHz, D_2O) δ 8.35 – 8.21 (m, 2H), 8.03 – 7.75 (m, 2H), 7.71 – 7.45 (m, 4H), 7.45 – 7.23 (m, 2H), 7.23 – 6.96 (m, 2H), 6.37 – 6.19 (m, 2H), 5.88 – 5.75 (m, 1H), 5.32 – 5.13 (m, 1H), 3.81 – 3.65 (m, 10H), 3.60 – 3.52 (m, 2H), 3.51 – 3.40 (m, 2H), 3.38 – 3.23 (m, 2H), 3.22 – 3.07 (m, 1H), 3.07 – 2.87 (m, 3H), 2.79 (s, 1H), 2.71 (s, 1H), 2.57 (d, J = 12.5 Hz, 1H), 2.54 – 2.40 (m, 2H).

Synthesis of compound S20

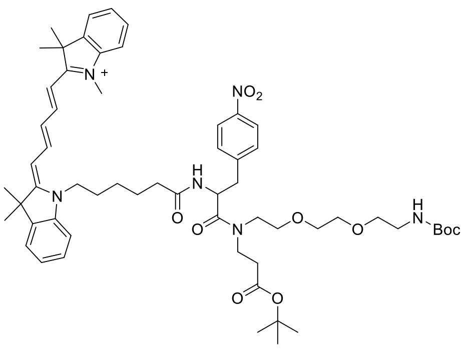

Blue solid; Yield: 80% (65 mg); LC-MS (ESI$^+$): calculated for $C_{59}H_{81}N_6O_{10}$ $[M]^+$ m/z: 1033.60; found: 1033.80; mixture of rotamers; ^{1}H NMR (400 MHz, $CDCl_3$) δ 8.13 (td, J = 8.5, 2.9 Hz, 2H), 7.88 – 7.80 (m, 2H), 7.51 – 7.31 (m, 5H), 7.22 (td, J = 7.5, 2.4 Hz, 2H), 7.16 – 7.01 (m, 2H), 6.75 (t, J = 12.5 Hz, 1H), 6.31 – 5.97 (m, 2H), 5.28 – 5.15 (m, 1H), 5.14 – 4.98 (m, 1H), 4.04 – 3.78 (m, 2H), 3.70 – 3.58

(m, 6H), 3.58 – 3.50 (m, 7H), 3.46 – 3.42 (m, 2H), 3.33 – 3.24 (m, 2H), 3.23 – 3.12 (m, 1H), 3.10 – 2.98 (m, 1H), 2.55 – 2.44 (m, 2H), 2.28 – 2.11 (m, 2H), 1.89 – 1.47 (m, 18H), 1.43 – 1.37 (m, 18H).

Synthesis of compound S21

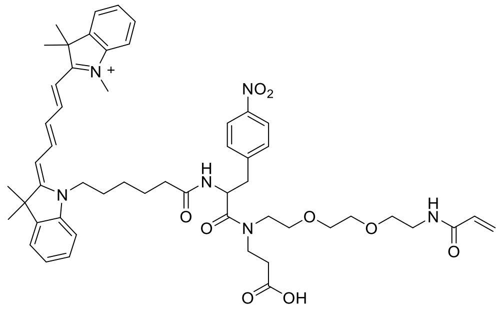

Yellow solid; Yield: 52% (40 mg); LC-MS (ESI+): calculated for $C_{53}H_{67}N_6O_9$ $[M]^+$ m/z: 931.50; found: 931.25; mixture of rotamers; ^{1}H NMR (400 MHz, MeOD-*d*$_4$) δ 8.25 (t, *J* = 13.0 Hz, 2H), 8.16 (dd, *J* = 8.4, 3.7 Hz, 2H), 7.58 – 7.46 (m, 4H), 7.45 – 7.39 (m, 2H), 7.34 – 7.24 (m, 4H), 6.64 (t, *J* = 12.4 Hz, 1H), 6.25 (ddd, *J* = 10.8, 9.8, 3.2 Hz, 4H), 5.68 – 5.60 (m, 1H), 5.27 – 5.22 (m, 1H), 4.05 (brs, 2H), 3.66 – 3.62 (m, 6H), 3.60 – 3.55 (m, 6H), 3.50 – 3.37 (m, 5H), 3.27 – 3.19 (m, 1H), 3.02 (t, *J* = 9.9 Hz, 1H), 2.65 – 2.54 (m, 2H), 2.23 – 2.16 (m, 2H), 1.73 (s, 12H), 1.61 – 1.54 (m, 2H), 1.44 – 1.23 (m, 4H); ^{13}C NMR (101 MHz, MeOD-*d*$_4$) δ 175.49, 174.65, 173.03, 168.23, 163.38, 163.01, 155.65, 148.37, 147.01, 144.31, 143.59, 142.71, 142.62, 132.12, 131.96, 129.82, 126.82, 126.35, 124.55, 123.49, 123.35, , 119.79, 116.88, 112.06, 111.94, 104.54, 104.31, 71.73, 71.50, 71.10, 71.32, 70.66, 70.54, 70.18, 51.30, 50.60, 44.81, 39.21, 38.95, 36.32, 33.59, 31.65, 28.173, 28.06, 27.28, 26.49.

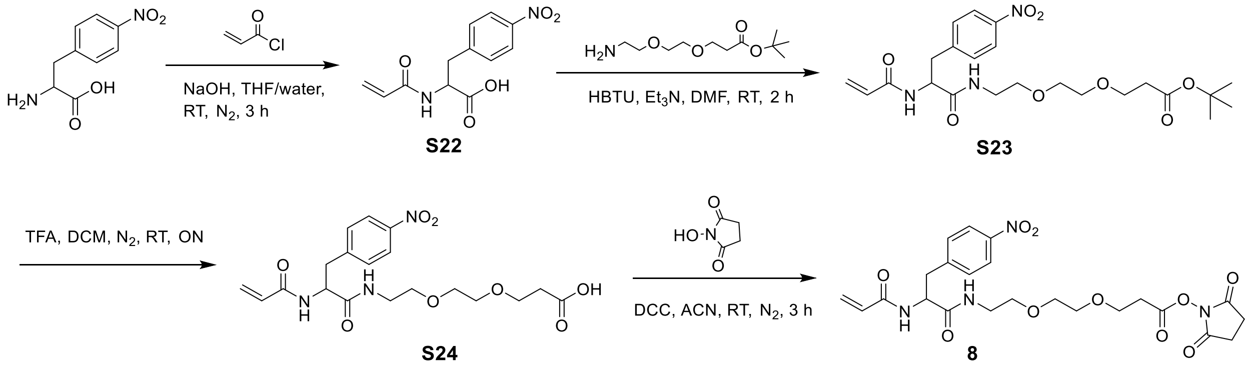

Synthesis of compound S22

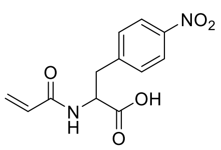

A solution of acryloyl chloride (0.846 mL, 10.47 mmol) in THF (10 mL) was added dropwise to a solution of 4-nitro-L-phenylalanine (2 g, 9.52 mmol) and sodium hydroxide (0.761 g, 19.03 mmol) in water (60 mL). The mixture was stirred at room temperature for 3 h. After complete reaction, the pH was adjusted to 3 by addition of HCl (6 M), followed by extraction with EtOAc (120 mL). The organic phase was then washed with water (60mL) and brine (60 mL). Afterwards, the organic phase was dried over $MgSO_4$ and evaporated under reduced to yield the product as a yellow solid (1.89 g, 75%). LC-MS (ESI+): calculated for $C_{12}H_{11}N_2O_5$ $[M-H]^-$ m/z: 263.07; found: 262.80; ^{1}H NMR (400 MHz, DMSO-*d*$_6$) δ 12.87 (brs, 1H), 8.50 (d, *J* = 8.3 Hz, 1H), 8.19 – 8.09 (m, 2H), 7.57 – 7.45 (m, 2H), 6.23 (dd, *J* = 17.1, 10.2 Hz, 1H), 6.04 (dd, *J* = 17.1, 2.1 Hz, 1H), 5.58 (dd, *J* = 10.2, 2.1 Hz, 1H), 4.61 (ddd, *J* = 9.6, 8.4, 4.9 Hz, 1H), 3.25 (dd, *J* = 13.8, 4.9 Hz, 1H), 3.04 (dd, *J* = 13.8, 9.7 Hz, 1H).

Synthesis of compound S23

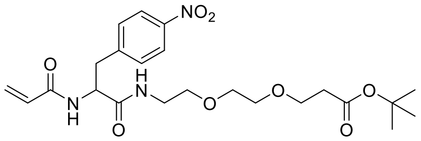

To a solution of the intermediate S22 (0.604 g, 2.29 mmol) in DMF (3 mL), HBTU (0.96 g, 2.53 mmol) was added. After 10 minutes, the mixture was cooled to 0 °C with an ice bath and stirred for 5 minutes. A solution of tert-butyl 3-(2-(2-aminoethoxy)ethoxy)propanoate (0.64 g, 2.74 mmol) and triethylamine (0.96 mL, 6.91 mmol) in DMF (2 mL) was then added dropwise. The reaction mixture was allowed to come to room temperature over 1.5 h. After complete reaction, 100 mL ethyl acetate was added into the reaction flask, followed by washing with water (100 mL, 2 x) and brine (100 mL). The organic layer was dried over $MgSO_4$ and then evaporated under reduced pressure. The residue was purified by column chromatography to yield the intermediate as a yellow solid (0.592 g, 54%). LC-MS (ESI$^+$): calculated for $C_{23}H_{34}N_3O_8$ $[M+H]^+$ m/z: 480.23; found: 479.95; ^1H NMR (400 MHz, $CDCl_3$) δ 8.18 – 8.03 (m, 2H), 7.48 – 7.34 (m, 2H), 6.74 (brs, 1H), 6.60 (d, *J* = 7.8 Hz, 1H), 6.28 (dd, *J* = 17.0, 1.4 Hz, 1H), 6.10 (dd, *J* = 17.0, 10.3 Hz, 1H), 5.67 (dd, *J* = 10.2, 1.4 Hz, 1H), 4.84 (dd, *J* = 14.4, 6.7 Hz, 1H), 3.70 (t, *J* = 6.2 Hz, 2H), 3.58 – 3.48 (m, 5H), 3.47 – 3.36 (m, 3H), 3.29 (dd, *J* = 13.7, 6.9 Hz, 1H), 3.17 (dd, *J* = 13.6, 6.3 Hz, 1H), 2.49 (td, *J* = 6.2, 1.3 Hz, 2H), 1.44 (s, 9H).

Synthesis of compound S24

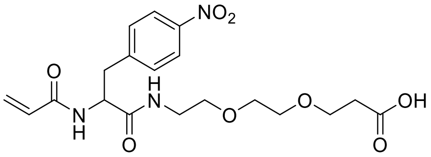

To a solution of the intermediate S23 (1 g, 2.09 mmol) in DCM (2 mL), TFA (2 mL) was added and the reaction mixture was stirred overnight at room temperature. After complete reaction, all solvents were evaporated and the residue was purified by column chromatography to yield the product as a yellow oil (0.706 g, 80%). LC-MS (ESI$^+$): calculated for $C_{19}H_{24}N_3O_8$ $[M-H]^-$ m/z: 422.16; found: 421.85; ^1H NMR (400 MHz, MeOD-d_4) δ 8.18 – 8.09 (m, 2H), 7.48 (t, *J* = 5.5 Hz, 2H), 6.28 – 6.12 (m, 2H), 5.63 (dd, *J* = 9.9, 2.0 Hz, 1H), 4.76 (dd, *J* = 8.5, 6.4 Hz, 1H), 3.72 – 3.67 (m, 2H), 3.59 – 3.50 (m, 4H), 3.50 – 3.30 (m, 4H), 3.27 – 3.20 (m, 1H), 3.05 (dd, *J* = 13.7, 8.5 Hz, 1H), 2.52 (dd, *J* = 8.4, 4.0 Hz, 2H); ^{13}C NMR (101 MHz, MeOD-d_4) δ 175.34, 173.88, 172.69, 167.67, 148.37, 146.49, 131.52, 127.50, 124.44, 71.27, 70.39, 67.71, 55.53, 49.00, 40.43, 38.99, 35.69.

Synthesis of compound 8

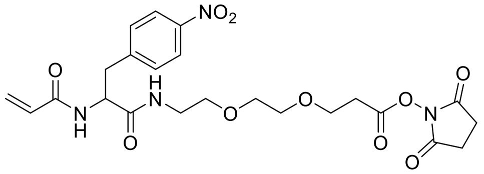

To a solution of the intermediate S24 (25 mg, 59.1 μmol) in THF (2.5 mL), DIC (18.5 μL, 118.2 μmol), N-Hydroxysuccinimide (8.15 mg, 70.9 μmol) and triethylamine (9.0 μL, 65.0 μmol) were added at 0 °C with an ice bath. The reaction mixture was further stirred at room temperature for 2 h. After complete reaction, the mixture was filtered to remove the white precipitate and the filtrate was evaporated under reduced pressure. The residue was further triturated with EtOAc (1 mL) in an ice bath. The mixture was filtered again to remove the precipitate. The filtrate was evaporated under reduced pressure and the residue was used for coupling without further purification. LC-MS (ESI$^+$): calculated for $C_{23}H_{29}N_4O_{10}$ $[M+H]^+$ m/z: 521.19; found: 520.95.

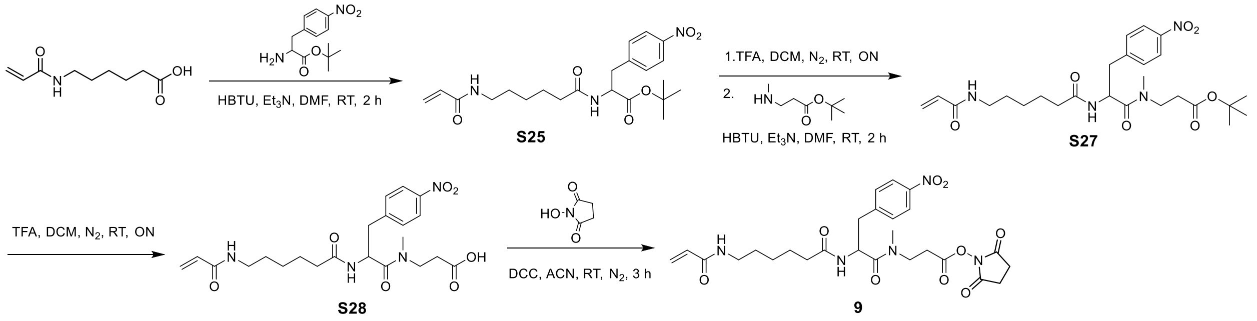

Synthesis of compound S25

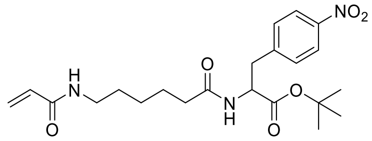

To a solution of 6-acrylamidohexanoic acid[3] (183.4 mg, 0.991 mmol) in DMF (3 mL), triethylamine (0.412 mL, 2.97 mmol) and HBTU (413 mg, 1.09 mmol) were added. After 10 minutes, tert-Butyl 4-nitro-L-phenylalaninate (290 mg, 1.09 mmol) was added and the reaction mixture was stirred at room temperature for 2 h. After complete reaction, 50 mL ethyl acetate was added into the reaction flask, followed by washing with water (50 mL, 2 x) and brine (50 mL). The organic layer was dried over $MgSO_4$ and then evaporated under reduced pressure. The residue was purified by column chromatography to yield the intermediate as a yellow oil (249 mg, 58%). LC-MS (ESI^+): calculated for $C_{22}H_{32}N_3O_6$ $[M+H]^+$ m/z: 434.23; found: 433.90; ^{1}H NMR (400 MHz, $CDCl_3$) δ 8.16 (d, *J* = 8.6 Hz, 2H), 7.34 (d, *J* = 8.6 Hz, 2H), 6.28 (dd, *J* = 17.0, 1.2 Hz, 1H), 6.15 – 6.06 (m, 1H), 6.06 – 5.98 (m, 1H), 5.73 (brs, 1H), 5.63 (dd, *J* = 10.2, 1.2 Hz, 1H), 4.78 (dd, *J* = 13.1, 6.4 Hz, 1H), 3.34 (dd, *J* = 13.0, 6.6 Hz, 2H), 3.20 (ddd, *J* = 19.6, 13.8, 6.1 Hz, 2H), 2.20 (t, *J* = 7.2 Hz, 2H), 1.67 – 1.61 (m, 4H), 1.42 (s, 9H), 1.38 – 1.31 (m, 2H).

Synthesis of compound S26

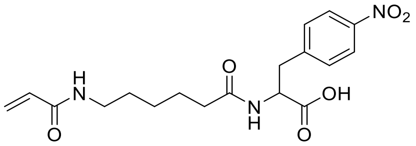

To a solution of the intermediate S25 (1 g, 2.31 mmol) in DCM (2 mL), TFA (2 mL) was added and the reaction mixture was stirred overnight at room temperature. After complete reaction, all solvents were evaporated and the residue was purified by column chromatography to yield the product as a yellow oil (0.74 g, 85%). LC-MS (ESI^+): calculated for $C_{18}H_{24}N_3O_6$ $[M+H]^+$ m/z: 378.17; found: 377.90; ^{1}H NMR (400 MHz, MeOD-*d*$_4$) δ 8.19 – 8.11 (m, 2H), 7.51 – 7.42 (m, 2H), 6.26 – 6.15 (m, 2H), 5.61 (dd, *J* = 8.0, 4.1 Hz, 1H), 4.74 (dd, *J* = 9.6, 5.0 Hz, 1H), 3.34 (dd, *J* = 14.0, 5.0 Hz, 1H), 3.18 (t, *J* = 7.4 Hz, 2H), 3.06 (dd, *J* = 14.0, 9.6 Hz, 1H), 2.18 – 2.11 (m, 2H), 1.55 – 1.43 (m, 4H), 1.28 – 1.17 (m, 2H); ^{13}C NMR (101 MHz, MeOD-*d*$_4$) δ 176.04, 174.18, 173.10, 168.17, 148.45, 146.84, 146.54, 132.17, 131.55 , 126.56, 124.54, 54.89, 54.47, 54.31, 40.26, 38.29, 38.15, 36.61, 36.54, 30.04, 27.42, 26.49, 20.35.

Synthesis of compound S27

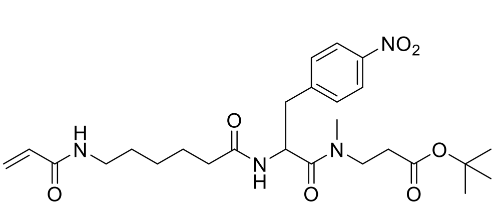

To a solution of the intermediate S26 (64 mg, 0.170 mmol) in DMF (1 mL), triethylamine (71 μL, 0.509 mmol) and HATU (77 mg, 0.204 mmol) were added. After 10 minutes, tert-butyl 3-(methylamino)propanoate (35 mg, 0.221 mmol) was added and the reaction mixture was stirred at room temperature for 2 h. After complete reaction, 30 mL ethyl acetate was added into the reaction flask, followed by

washing with water (30 mL, 2 x) and brine (30 mL). The organic layer was dried over $MgSO_4$ and then evaporated under reduced pressure. The residue was purified by column chromatography to yield the intermediate as a yellow oil (47.5 mg, 54%). LC-MS (ESI$^+$): calculated for $C_{26}H_{39}N_4O_7$ $[M+H]^+$ m/z: 519.28; found: 519.00; mixture of rotamers; ^{1}H NMR (400 MHz, $CDCl_3$) δ 8.21 – 8.07 (m, 2H), 7.35 (dd, *J* = 16.4, 8.7 Hz, 2H), 6.50 – 6.34 (m, 1H), 6.27 (dd, *J* = 17.0, 1.5 Hz, 1H), 6.19 – 6.00 (m, 1H), 5.85 (brs, 1H), 5.61 (dt, *J* = 10.2, 2.6 Hz, 1H), 5.28 – 5.12 (m, 1H), 3.67 – 3.55 (m, 1H), 3.54 – 3.43 (m, 1H), 3.38 – 3.26 (m, 2H), 3.18 – 3.13 (m, 1H), 3.06 – 2.97 (m, 1H), 2.93 (s, 2H), 2.89 (s, 1H), 2.49 – 2.34 (m, 2H), 2.16 (q, *J* = 7.7 Hz, 2H), 1.60 (dt, *J* = 15.0, 7.4 Hz, 2H), 1.52 (dd, *J* = 14.6, 7.2 Hz, 2H), 1.42 (d, *J* = 8.5 Hz, 9H), 1.37 – 1.22 (m, 2H).

Synthesis of compound S28

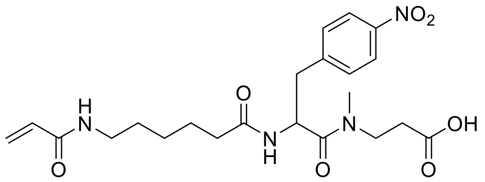

To a solution of the intermediate S27 (45 mg, 87 μmol) in DCM (0.1 mL), TFA (0.1 mL) was added and the reaction mixture was stirred overnight at room temperature. After complete reaction, all solvents were evaporated and the residue was purified by column chromatography to yield the product as a yellow oil (34 mg, 84%). LC-MS (ESI$^+$): calculated for $C_{22}H_{29}N_4O_7$ $[M-H]^-$ m/z: 461.20; found: 460.90; mixture of rotamers; ^{1}H NMR (400 MHz, MeOD-*d*$_4$) δ 8.16 (dd, *J* = 8.8, 2.6 Hz, 2H), 7.51 (dd, *J* = 12.2, 8.7 Hz, 2H), 6.30 – 6.10 (m, 2H), 5.63 (dd, *J* = 8.3, 3.7 Hz, 1H), 5.30 – 5.09 (m, 1H), 3.86 – 3.58 (m, 1H), 3.57 – 3.46 (m, 1H), 3.29 – 3.14 (m, 3H), 3.13 – 2.94 (m, 3H), 2.90 (s, 1H), 2.56 (ddd, *J* = 9.5, 6.8, 2.8 Hz, 1H), 2.49 (t, *J* = 7.1 Hz, 1H), 2.23 – 2.09 (m, 2H), 1.63 – 1.41 (m, 4H), 1.36 – 1.14 (m, 2H). ^{13}C NMR (101 MHz, MeOD-*d*$_4$) δ 175.58, 175.51, 175.22, 174.59, 173.05, 172.79, 168.17, 148.47, 146.75, 146.41, 132.20, 131.84, 131.78, 126.57, 124.54, 51.51, 51.28, 46.64, 46.13, 40.26, 39.05, 38.69, 36.73, 36.49, 34.08, 33.72, 32.88, 30.06, 27.49, 26.49.

Synthesis of compound 9

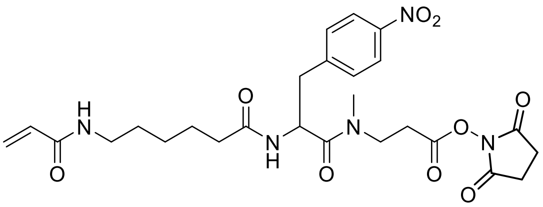

To a solution of the intermediate S28 (40 mg, 87 μmol) in THF (3 mL), DIC (27 μL, 173 μmol), N-Hydroxysuccinimide (13 mg, 113 μmol) and triethylamine (13 μL, 95 μmol) were added at 0 °C with an ice bath. The reaction mixture was further stirred at room temperature for 2 h. After complete reaction, the mixture was filtered to remove the white precipitate and the filtrate was evaporated under reduced pressure. The residue was further triturated with EtOAc (1 mL) in an ice bath. The mixture was filtered again to remove the precipitate. The filtrate was evaporated under reduced pressure and the residue was used for coupling without further purification. LC-MS (ESI$^+$): calculated for $C_{26}H_{34}N_5O_9$ $[M+H]^+$ m/z: 560.24; found: 560.00.

Synthesis of compound S29

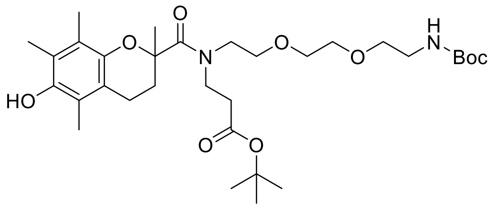

To a solution of the Trolox (0.23 g, 0.920 mmol) in DMF (1.5 mL), triethylamine (0.383 mL, 2.757 mmol) and HBTU (0.383 g, 1.011 mmol) were added. After 10 minutes, the amine diPEG linker 3 (0.415 g, 1.103 mmol, synthesized according to literature[2]) was added and the reaction mixture was stirred at room temperature for 2 h. After complete reaction, as indicated by TLC, 100 mL water was added into the reaction flask, followed by extraction with ethyl acetate (100 mL). The organic phase was further washed with brine (100

mL, 2 x). And then it was dried over $MgSO_4$ and evaporated to dryness. The residue was purified by column chromatography to obtain the product as a light yellow oil (0.531 g, 95%). LC-MS (ESI$^+$): calculated for $C_{32}H_{53}N_2O_9$ $[M+H]^+$ m/z: 609.37; found: 609.10; ^{1}H NMR (400 MHz, $CDCl_3$) δ 4.12 – 3.90 (m, 2H), 3.63 – 3.34 (m, 9H), 3.34 – 3.19 (m, 3H), 2.76 – 2.60 (m, 2H), 2.58 – 2.30 (m, 3H), 2.20 – 2.11 (m, 6H), 2.08 (d, *J* = 2.7 Hz, 3H), 1.44 (s, 16H), 1.39 (brs, 4H).

Synthesis of compound S30

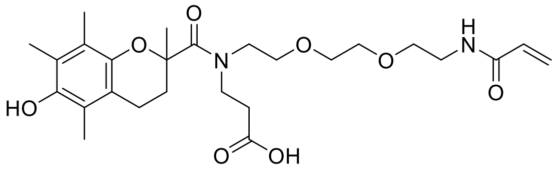

To a solution of the intermediate S29 in DCM (1 mL), TFA (1 mL) was added and the reaction mixture was stirred overnight at room temperature. After complete reaction, all solvents were evaporated to yield the intermediate as a yellow oil, which was used without further purification.

To a solution of the intermediate (100 mg, 177 μmol) in MeOH: THF (*v*:*v* = 0.4 mL: 0.4 mL), a solution of Na_2CO_3 (43.1 mg, 406 μmol) in water (0.4 mL) was added under the protection of N_2, followed by cooling the reaction flask to 0 °C with an ice bath. A solution of acryloyl chloride (14.4 μL, 230 μmol) in dry dioxane (0.3 mL) was added dropwise to the reaction flask, and the reaction mixture was then allowed to come to room temperature over 20 min. After complete reaction, all solvents were evaporated and the residue was purified by column chromatography to yield the product as a light yellow solid (38.4 mg, 43%). LC-MS (ESI$^+$): calculated for $C_{26}H_{39}N_2O_8$ $[M+H]^+$ m/z: 507.27; found: 506.95; ^{1}H NMR (600 MHz, MeOD-*d*$_4$) δ 6.36 – 6.20 (m, 2H), 5.67 (dt, *J* = 9.9, 2.1 Hz, 1H), 4.23 – 3.97 (m, 2H), 3.66 – 3.51 (m, 6H), 3.50 – 3.38 (m, 5H), 3.31 – 3.24 (m, 1H), 2.70 – 2.60 (m, 2H), 2.60 – 2.37 (m, 3H), 2.15 (dd, *J* = 8.4, 6.7 Hz, 6H), 2.08 (d, *J* = 4.4 Hz, 3H), 1.69 – 1.61 (m, 4H); ^{13}C NMR (151 MHz, MeOD-*d*$_4$) δ 175.92, 175.75, 175.33, 168.37, 147.31, 147.26, 146.19, 146.02, 132.17, 126.87, 124.85, 122.80, 122.65, 122.50, 122.35, 119.05, 119.01, 81.04, 80.98, 71.77, 71.71, 71.46, 71.11, 70.67, 70.00, 57.62, 46.35, 45.96, 40.60, 34.46, 33.57, 33.37, 32.99, 26.45, 22.45, 22.29, 17.43, 12.96, 12.59, 11.95.

Synthesis of compound 10

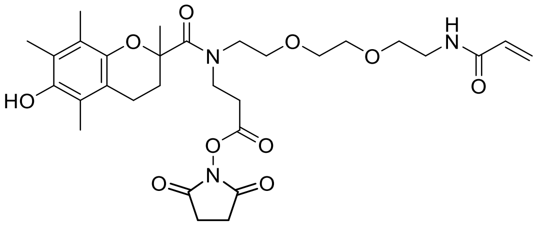

To a solution of the intermediate S30 (50 mg, 99 μmol) in THF (3 mL), DIC (62 μL, 395 μmol), N-Hydroxysuccinimide (14.8 mg, 128 μmol) and triethylamine (13.7 μL, 99 μmol) were added at 0 °C with an ice bath. The reaction mixture was further stirred at room temperature for 2 h. After complete reaction, the mixture was filtered to remove the white precipitate and the filtrate was evaporated under reduced pressure. The residue was further triturated with EtOAc (1 mL) in an ice bath. The mixture was filtered again to remove the precipitate. The filtrate was evaporated under reduced pressure and the residue was used for coupling without further purification. LC-MS (ESI$^+$): calculated for $C_{30}H_{42}N_3O_{10}$ $[M+H]^+$ m/z: 604.29; found: 604.00.

1.3 Rotamer characterization

To demonstrate that these compounds display rotameric behavior, we chose two types of molecules that we synthesized, followed by variable temperature ^{1}H NMR experiments, using 600 MHz at different temperature. The results were shown in **Figure S12** and **S13**.

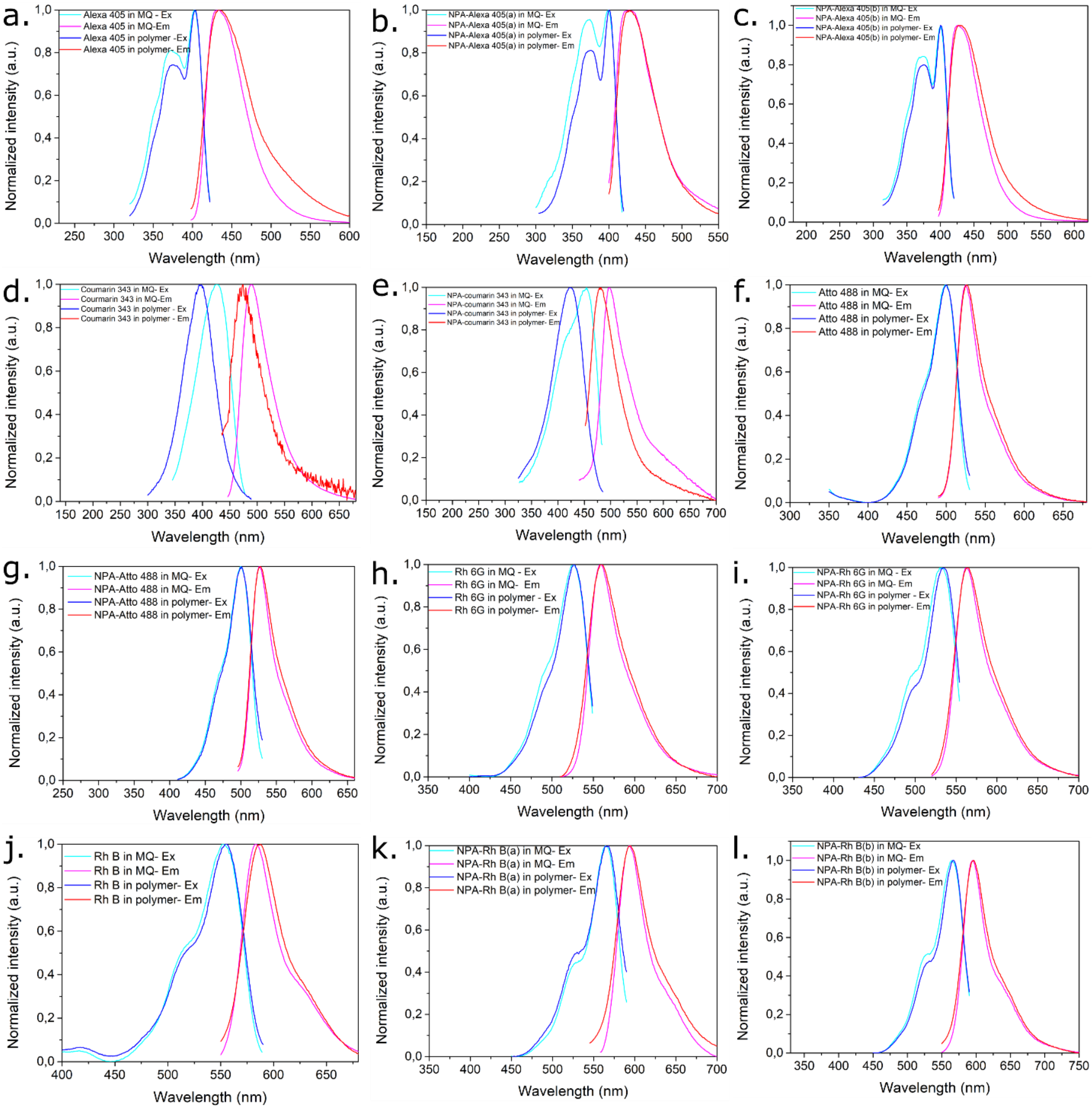

Figure S1. Excitation and emission properties of organic fluorophores in water and polymer. (a) Normalized excitation and emission spectra of Alexa 405 in water and polymer. (b) Normalized excitation and emission spectra of NPA-Alexa 405(a) in water and polymer. (c) Normalized excitation and emission spectra of NPA-Alexa 405(b) in water and polymer. (d) Normalized excitation and emission spectra of Coumarin 343 in water and polymer. (e) Normalized excitation and emission spectra of NPA-Coumarin 343 in water and polymer. (f) Normalized excitation and emission spectra of Atto 488 in water and polymer. (g) Normalized excitation and emission spectra of NPA-Atto 488 in water and polymer. (h) Normalized excitation and emission spectra of Rh 6G in water and polymer. (i) Normalized excitation and emission spectra of NPA-Rh 6G in water and polymer. (j) Normalized excitation and emission spectra of Rh B in water and polymer. (k) Normalized excitation and emission spectra of NPA-Rh B(a) in water and polymer. (l) Normalized excitation and emission spectra of NPA-Rh B(b) in water and polymer.

Table S1. Parameters used for signal retention evaluation

Dye	Concentration (μM)	Excitation wavelength (nm)	Emission range (nm)	Signal retention (Mean ± SD)[a]
Alexa 405	17	380	390-700	0.345 ± 0.015
NPA-Alexa 405(a)	17	370	390-700	6.021 ± 0.319
NPA-Alexa 405(b)	23	370	390-700	1.816 ± 0.027
Coumarin 343	10	420	440-700	0.104 ± 0.035
NPA-Coumarin 343	10	395	420-700	4.733 ± 0.300
Atto 488	9	480	490-700	0.821 ± 0.033
NPA-Atto 488	9	480	490-700	0.888 ± 0.027
Rh 6G	5	490	510-700	0.463 ± 0.0004
NPA-Rh 6G	5	490	510-700	2.183 ± 0.184
Rh B	7	520	535-700	0.563 ± 0.011

NPA-Rh B(a)	7	520	535-700	1.107 ± 0.017
NPA-Rh B(b)	7	520	535-700	1.186 ± 0.019
Cy5	2	600	620-800	0.0044 ± 0.00009
NPA-Cy5	2	600	620-800	0.0119 ± 0.00008

[a] Signal retention= Integrated emission intensity in polymer/Integrated emission intensity in water, n= 3 samples each.

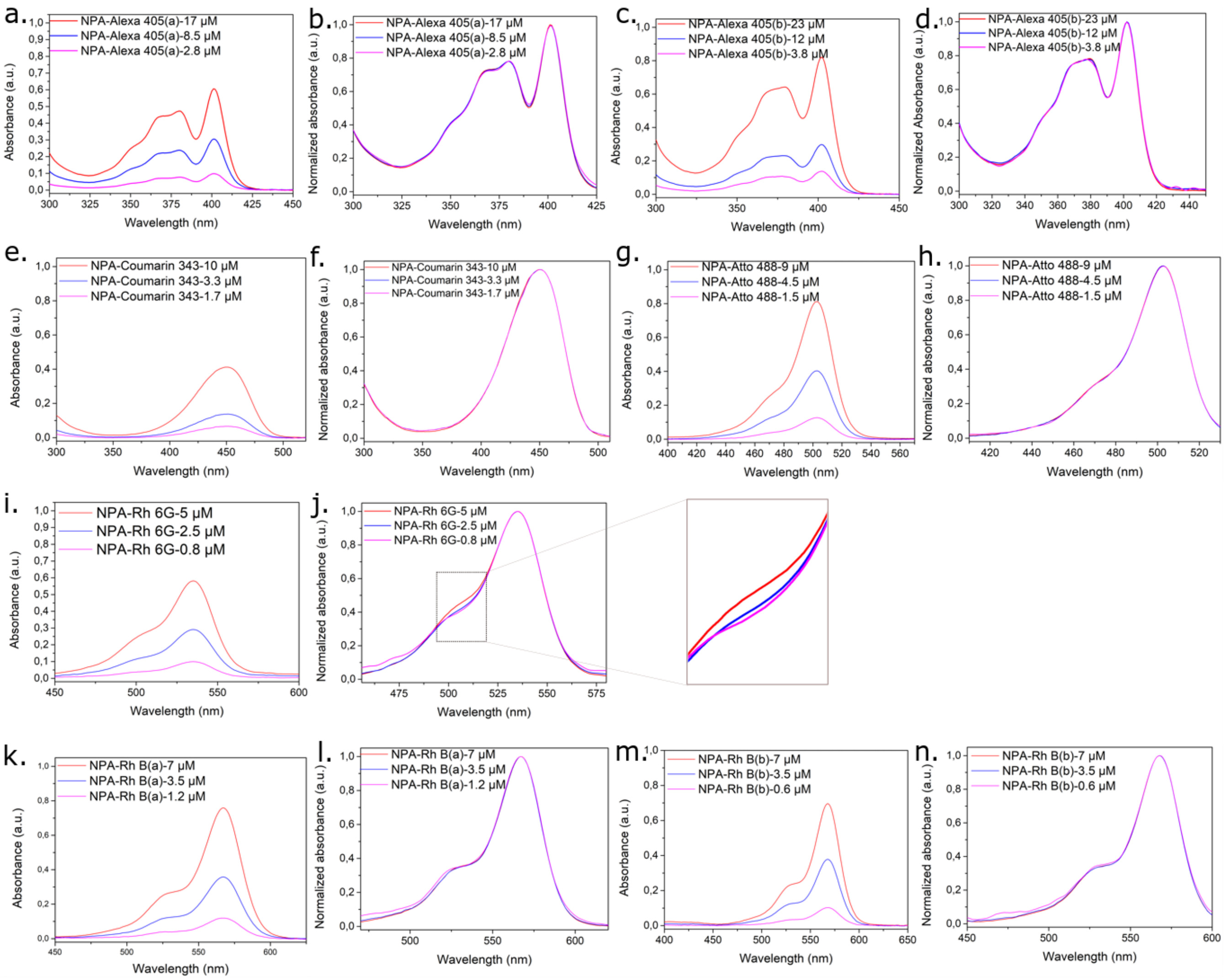

Figure S2. Characteristic of NPA-modified organic fluorophores dissolved in water. (a) Absorption spectra of NPA-Alexa(a) in different concentrations. (b) Normalized absorption spectra of (a). (c) Absorption spectra of NPA-Alexa(b) in different concentrations. (d) Normalized absorption spectra of (c). (e) Absorption spectra of NPA-Coumarin 343 in different concentrations. (f) Normalized absorption spectra of (e). (g) Absorption spectra of NPA-Atto 488 in different concentrations. (h) Normalized absorption spectra of (g). (i) Absorption spectra of NPA-Rh 6G in different concentrations. (j) Normalized absorption spectra of (i). (k) Absorption spectra of NPA-Rh B(a) in different concentrations. (l) Normalized absorption spectra of (k). (m) Absorption spectra of NPA-Rh B(b) in different concentrations. (n) Normalized absorption spectra of (m).

Table S2. Φ_f values and parameters used for determination at room temperature.

Dye	Concentration (μM)	Solvent	Excitation range (nm)	Emission range (nm)	Φf (Mean ± SD)[a]
Alexa 405	17	water	384-396	400-700	0.944 ± 0.003
Alexa 405	17	polymer	384-396	400-700	0.418 ± 0.005
NPA-Alexa 405(a)	17	water	361-379	385-700	0.017 ± 0.0006
NPA-Alexa 405(a)	17	polymer	361-379	385-700	0.208 ± 0.006
NPA-Alexa 405(b)	23	water	361-379	385-700	0.156 ± 0.002
NPA-Alexa 405(b)	23	polymer	361-379	385-700	0.390 ± 0.009
Coumarin 343	10	water	418-432	440-750	0.850 ± 0.001
Coumarin 343	10	polymer	418-432	440-750	0.289 ± 0.002
NPA-Coumarin 343	10	water	422-438	443-750	0.004 ± 0.0006
NPA-Coumarin 343	10	polymer	422-438	443-750	0.150 ± 0.014
Atto 488	9	water	479-491	495-700	0.914 ± 0.003
Atto 488	9	polymer	479-491	495-700	0.866 ± 0.009

NPA-Atto 488	9	water	479-491	495-700	0.461 ± 0.02
NPA-Atto 488	9	polymer	479-491	495-700	0.485 ± 0.007
Rh 6G	5	water	482-498	503-700	0.833 ± 0.010
Rh 6G	5	polymer	482-498	503-700	0.548 ± 0.006
NPA-Rh 6G	5	water	482-498	503-750	0.144 ± 0.011
NPA-Rh 6G	5	polymer	482-498	503-750	0.341 ± 0.014
Rh B	7	water	512-528	528-700	0.363 ±0.008
Rh B	7	polymer	512-528	528-700	0.214 ± 0.002
NPA-Rh B(a)	7	water	512-528	532-700	0.116 ± 0.024
NPA-Rh B(a)	7	polymer	512-528	532-700	0.158 ± 0.022
NPA-Rh B(b)	7	water	512-528	532-750	0.109 ± 0.001
NPA-Rh B(b)	7	polymer	511-529	529-750	0.153 ± 0.002
Cy5	2	water	591-609	615-800	0.105 ± 0.001
NPA-Cy5	2	water	591-609	615-800	0.044 ± 0.001
Rhodamine 6G[b]	5	ethanol	483-497	497-700	0.982 ± 0.013

[a] n= 3 samples each. [b] Control experiment: absolute quantum yield of Rh 6G in ethanol was measured and used as a reference.

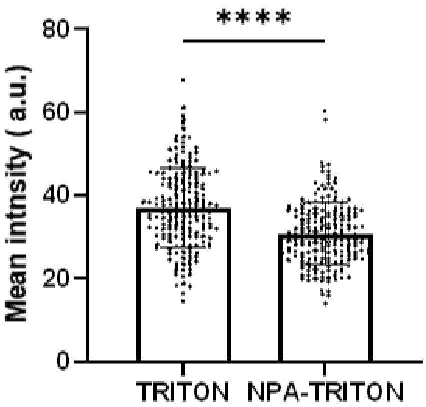

Figure S3. Comparison of pre-polymerization fluorescence intensity in immunostaining. Mean fluorescent intensity are determined and compared after immunostaining with TRITON (Rh B, acrylate and TFP) or NPA-TRITON (Compound **7**)-labeled secondary antibodies with the same parameters. Bars represent the mean value and error bars represent the standard deviation (n= 189 from three independent samples.). Statistical significance is assessed by t-test. **** p < 0.0001. From left to right, mean values are 37.01 ± 9.53 and 30.65 ± 7.49, respectively.

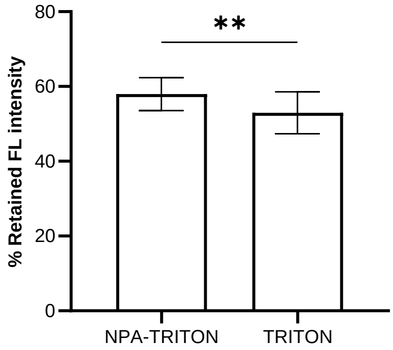

Figure S4. Comparison of retained fluorescent intensity of expanded microtubules in TRITON and NPA-TRION cases. Samples were illuminated under continuous 567 nm laser illumination for 123 seconds with a laser power of 48.6 μW (illumination area: 79.93 μm × 79.93 μm). Bars represent the mean value and error bars represent the standard deviation. Statistical significance is assessed by t-test. ** p < 0.01. From left to right, mean values are 0.58 ± 0.04 (mean ± standard deviation, n= 18 from three independent samples each), and 0.53 ± 0.06, respectively.

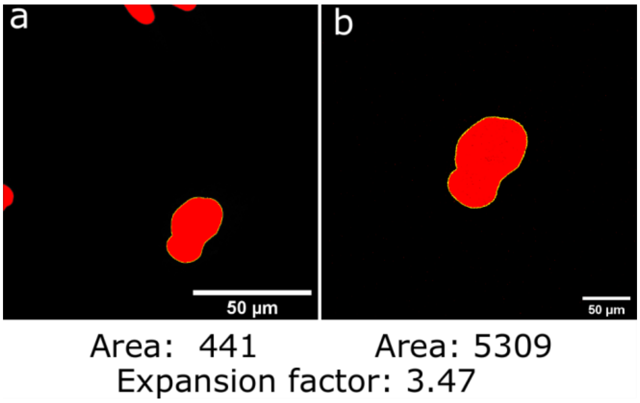

Figure S5. Expansion factor calculated in ExM. (a-b) The DAPI channel in pre- and post-image of the immunostaining protocol in ExM, in which the hydrogel was stabilized by a re-embedding protocol. It should be noted that nuclei calculated for expansion factor were from the same cells as the representative microtubules in the manuscript (**Figure 5e**-**5h**). Scale bars: 50 μm.

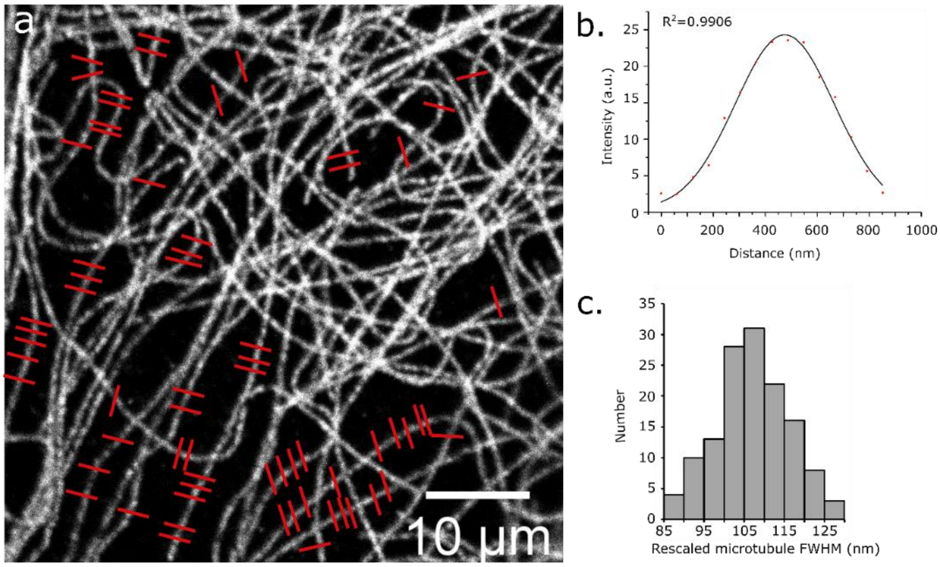

Figure S6. Quantification of resolution achieved in post-expansion images. (a) Representative FWHM line profiles of microtubules used for resolution determination were shown in red. (b) A representative cross-sectional intensity profile of microtubules (dots) with Guassian fitting (solid line). (c) Distribution of Gaussian-fitted full width at half maximum (FWHM) of microtubule intensity profiles along the red lines in (a), yielding a resolution of 107 ± 9 nm (mean ± s.d., n= 135).

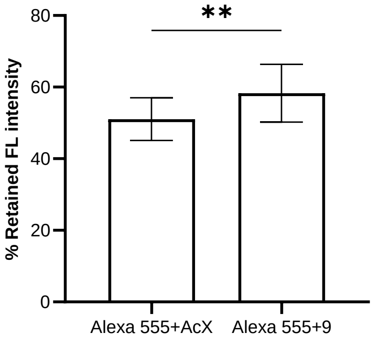

Figure S7. Comparison of retained fluorescent intensity of expanded microtubules in AcX and compound 9 cases. Samples were illuminated under continuous 553 nm laser illumination for 123 seconds with a laser power of 54.5 μW (illumination area: 79.93 μm × 79.93 μm). Bars represent the mean value and

error bars represent the standard deviation. Statistical significance is assessed by t-test. ** p < 0.01. From left to right, mean values are 0.51 ± 0.06 (mean ± standard deviation, n= 17 from three independent samples each), and 0.58 ± 0.08, respectively.

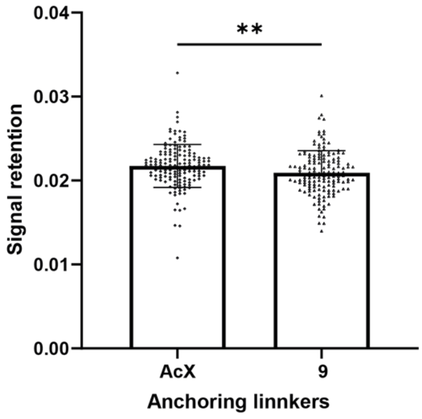

Figure S8. Comparison of signal retention of Alexa 647 in the polymerization step using different anchoring linkers. Bars represented the mean value and error bars represented the standard deviation. Statistical significance was assessed by one-way ANOVA test. **p < 0.01. From left to right, mean values were 0.022 ± 0.003 and 0.021 ± 0.003, respectively.

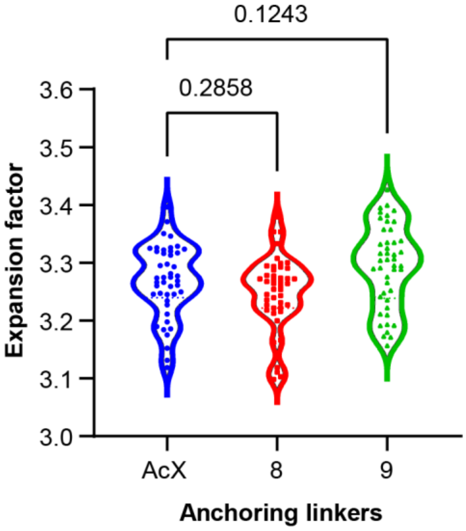

Figure S9. Comparison of expansion factor obtained with different anchoring linkers in ExM. Expanded gels were stabilized by a re-embedding protocol and expansion factor was calculated by comparing the same nuclei pre- and post-expansion. Bars represented the mean value and error bars represented the standard deviation (n= 45 from three independent samples). Statistical significance was assessed by one-way ANOVA test. From left to right, mean values are 3.27 ± 0.06 (mean ± standard deviation), 3.25 ± 0.06, and 3.29 ± 0.07, respectively.

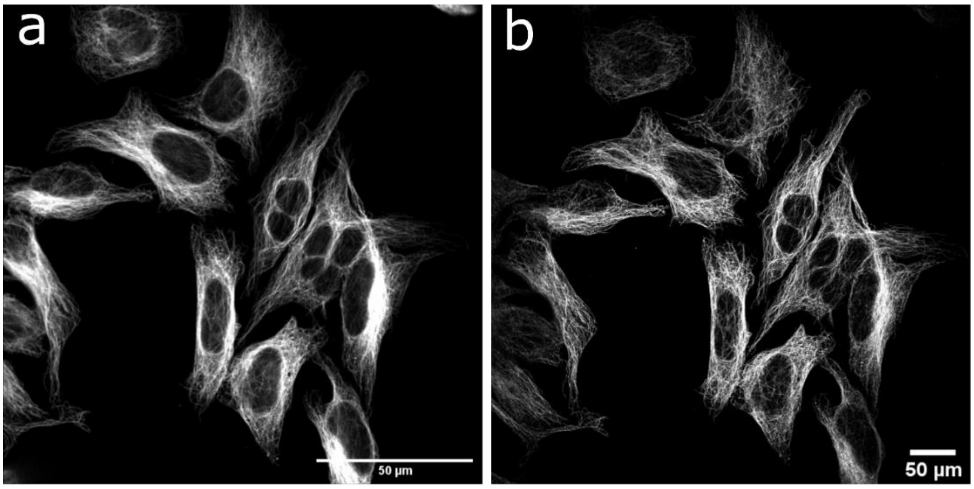

c

Reagents	Samples	Pre-gelation (microtubules)	Post-expansion (microtubules)	Retention
Compound 8	Sample 1	25	25	100%
	Sample 2	31	31	100%
	Sample 3	73	73	100%
	Sample 4	80	80	100%
	In total	209	209	100%

Figure S10. Quantification of anchoring efficiency of compound 8 in the immunostaining experiments against microtubules. (a) The representative image obtained in pre-expansion. (b) The representative image of the same cells, obtained in post-expansion. (c) Analysis of anchoring efficiency in four independent samples.

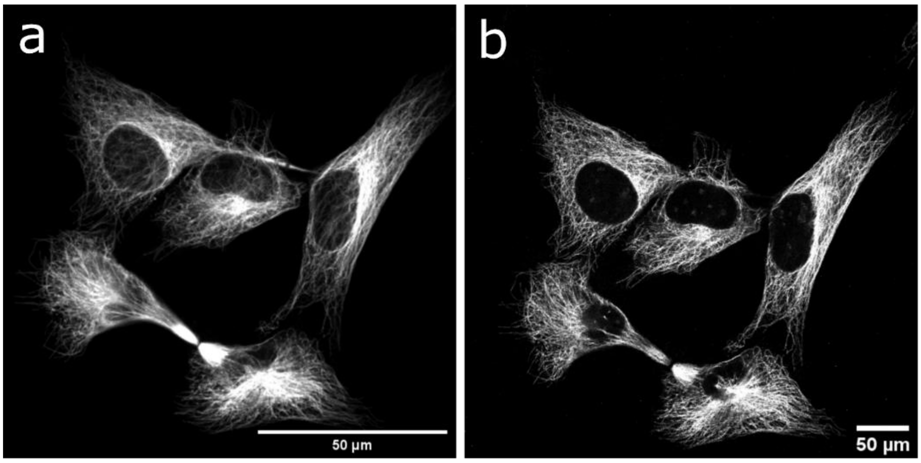

c

Reagents	Samples	Pre-gelation (microtubules)	Post-expansion (microtubules)	Retention
Compound 9	Sample 1	28	28	100%
	Sample 2	32	32	100%
	Sample 3	47	47	100%
	Sample 4	72	72	100%
	In total	179	179	100%

Figure S11. Quantification of anchoring efficiency of compound 9 in the immunostaining experiments against microtubules. (a) The representative image obtained in pre-expansion. (b) The representative image of the same cells, obtained in post-expansion. (c) Analysis of anchoring efficiency in four independent samples.

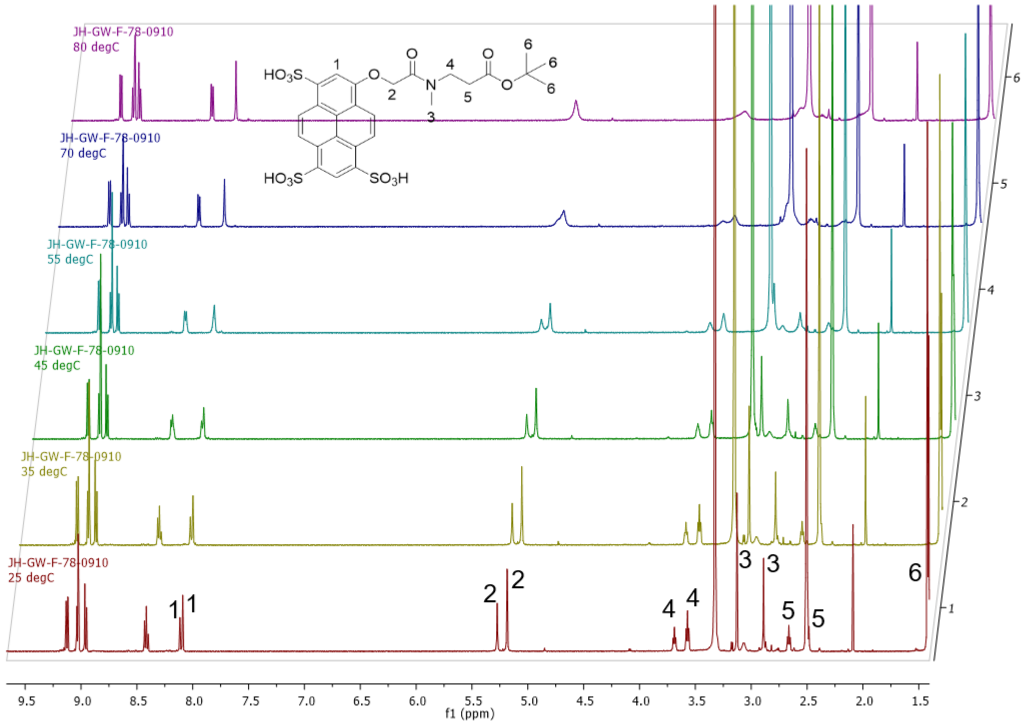

Figure S12. Variable temperature ¹H NMR spectra (600 MHz, DMSO-*d*$_6$) of compound S8. NMR spectra were performed at 25, 35, 45, 55, 70, 80 °C.

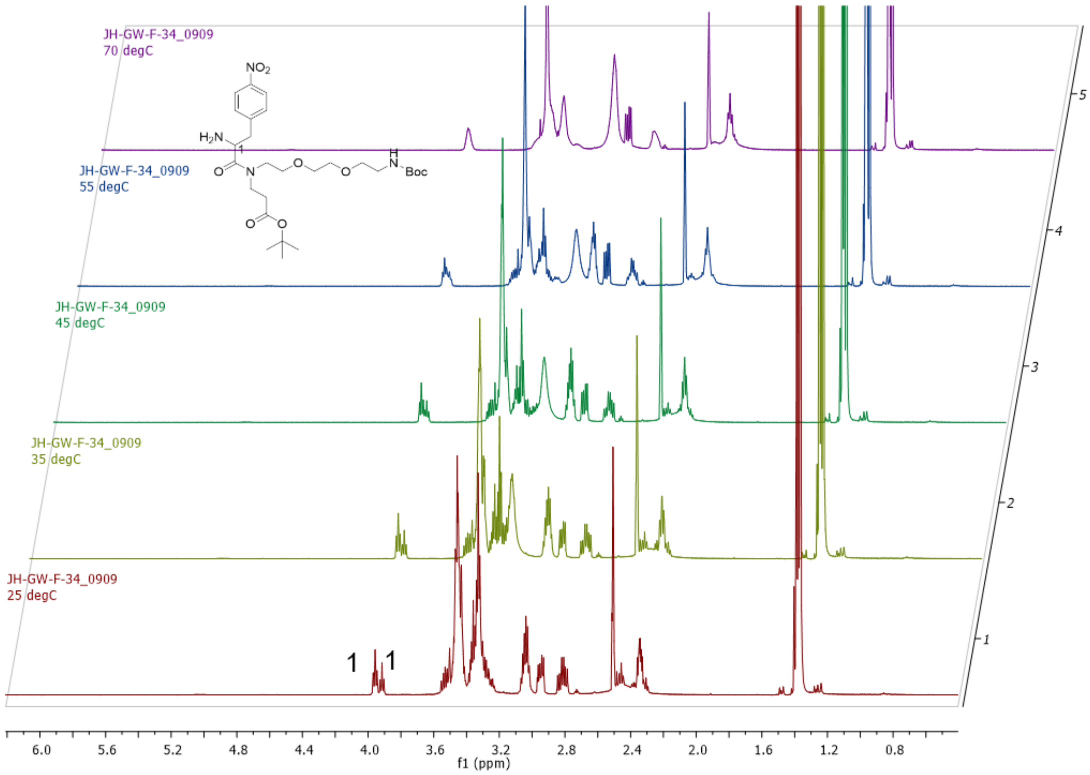

Figure S13. Partial variable temperature ¹H NMR spectra (600 MHz, DMSO-*d*$_6$) of compound 4. NMR spectra were performed at 25, 35, 45, 55, 70 °C.

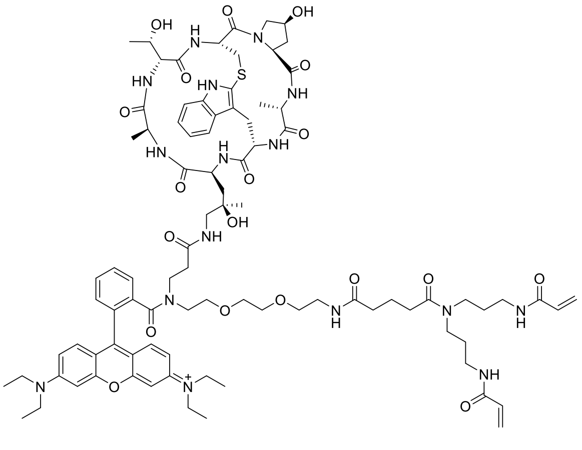

TRITON-2

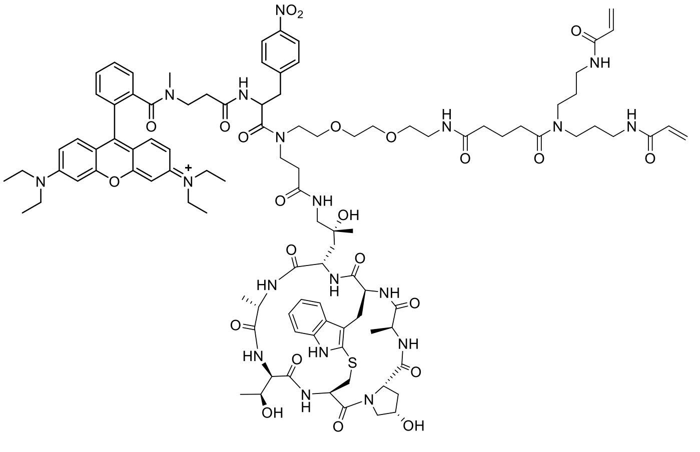

NPA-TRITON-2

Scheme S1. Chemical structures of TRITON-2 and NPA-TRITON-2.

Copies of ^{1}H and ^{13}C NMR spectra

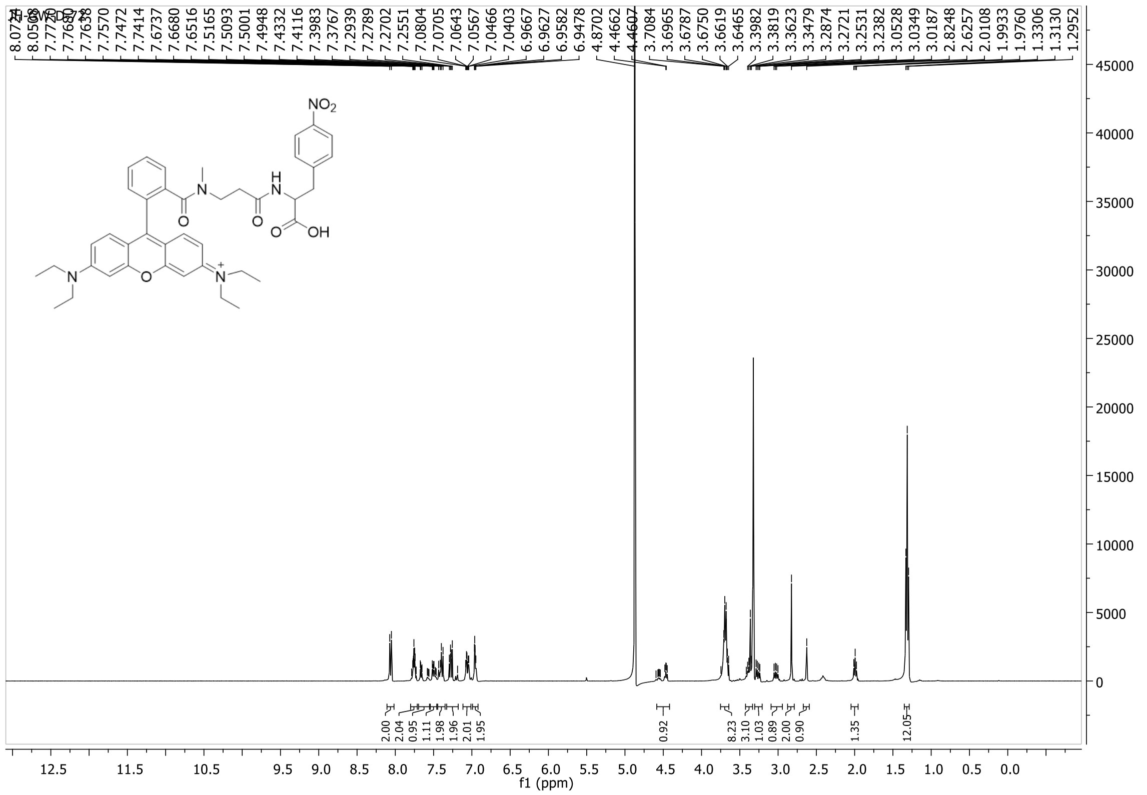

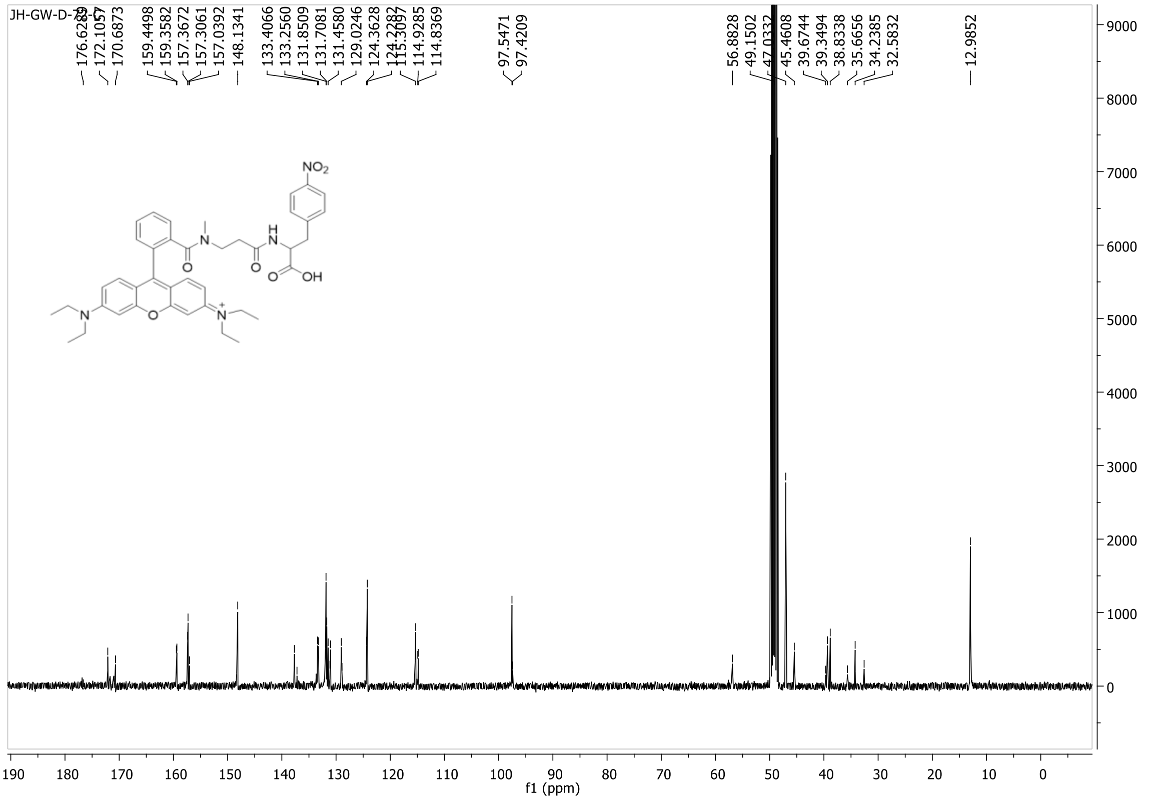

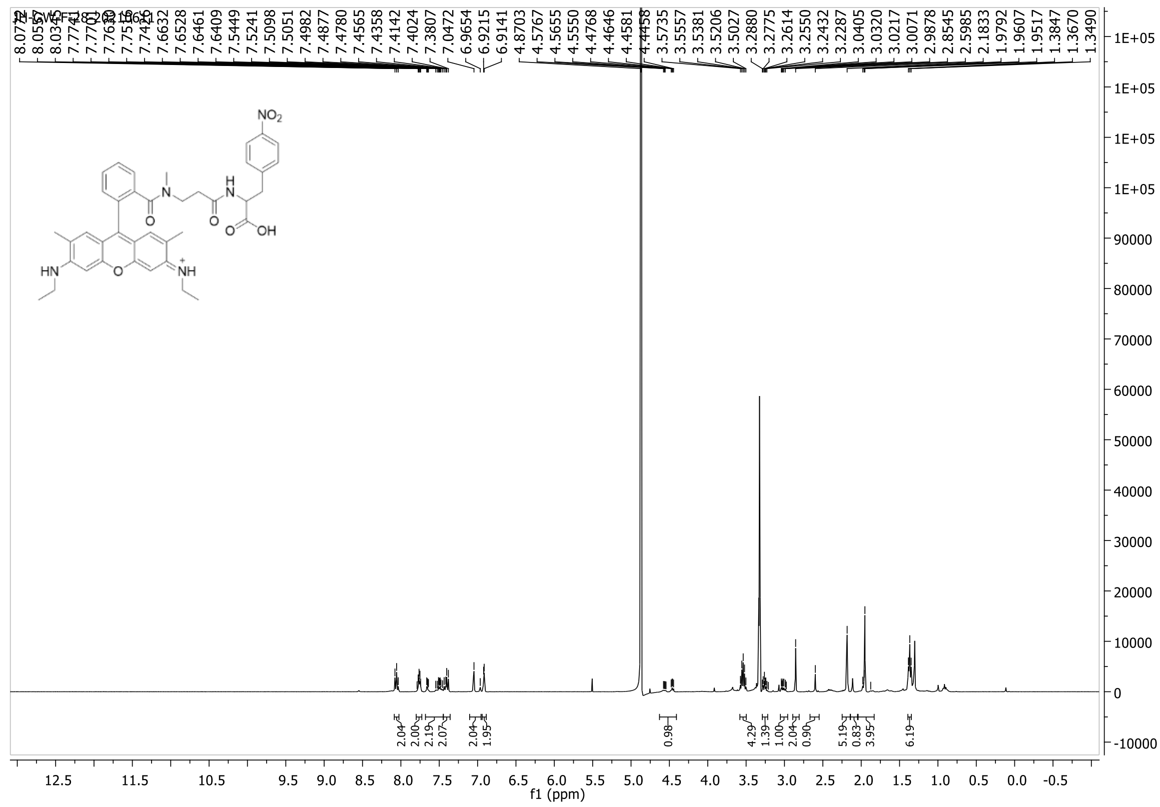

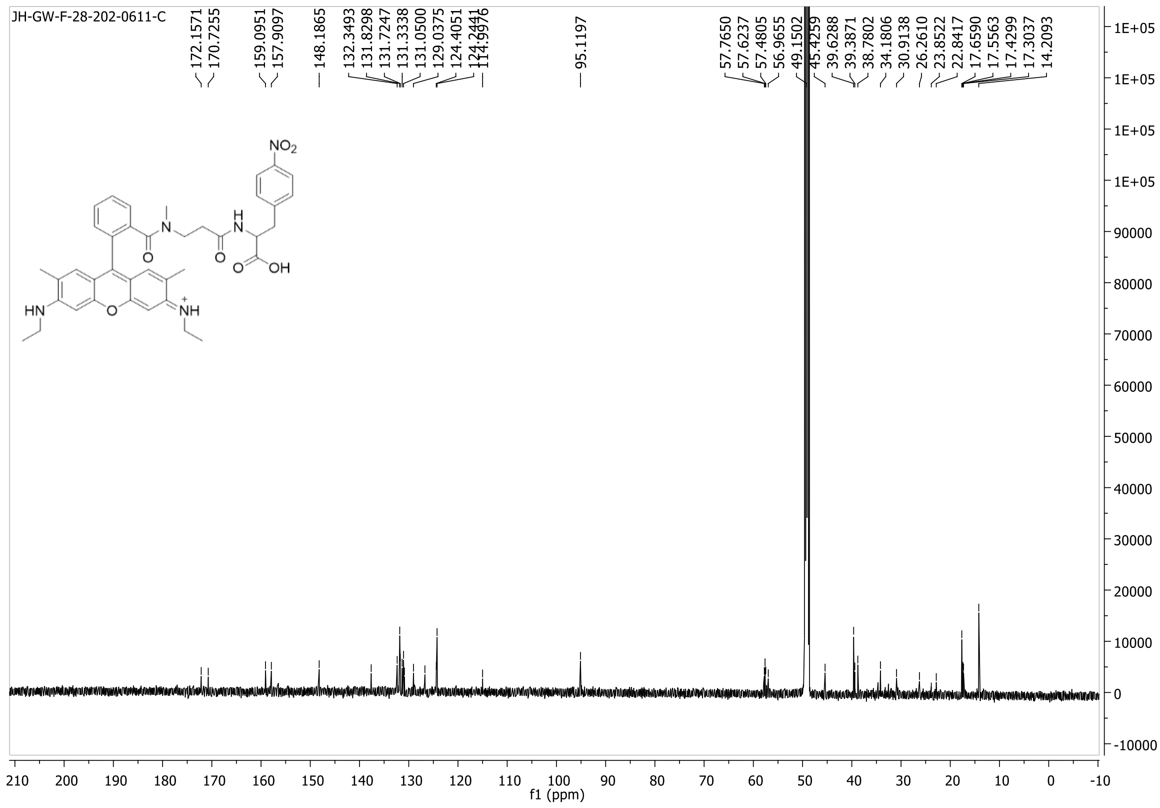

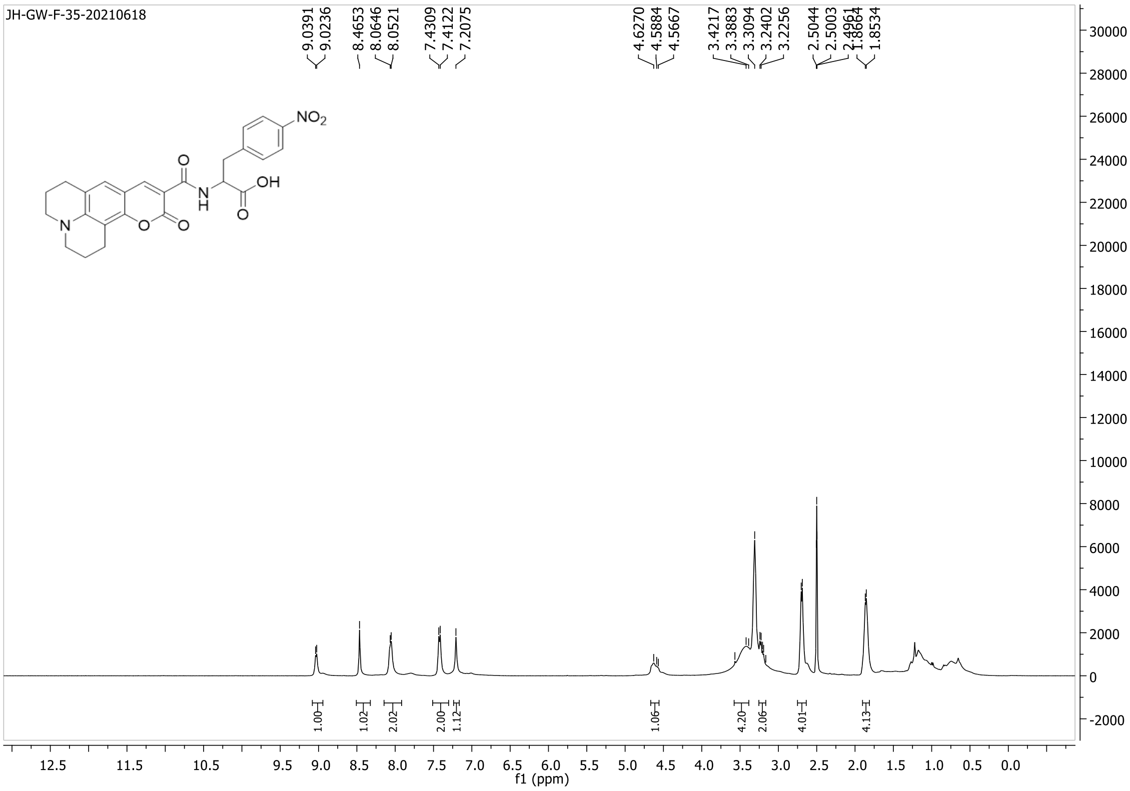
JH-GW-F-35-20210618
9.0391
9.0236
8.4653
8.0646
8.0521
7.4309
7.4122
7.2075
4.6270
4.5884
4.5667
3.4217
3.3883
3.3094
3.2402
3.2256
2.5044
2.5003
1.8534
NO2
O
N
H
OH
O
N
O
O
1.00
1.02
2.02
2.00
1.12
1.06
4.20
2.06
4.01
4.13
f1 (ppm)

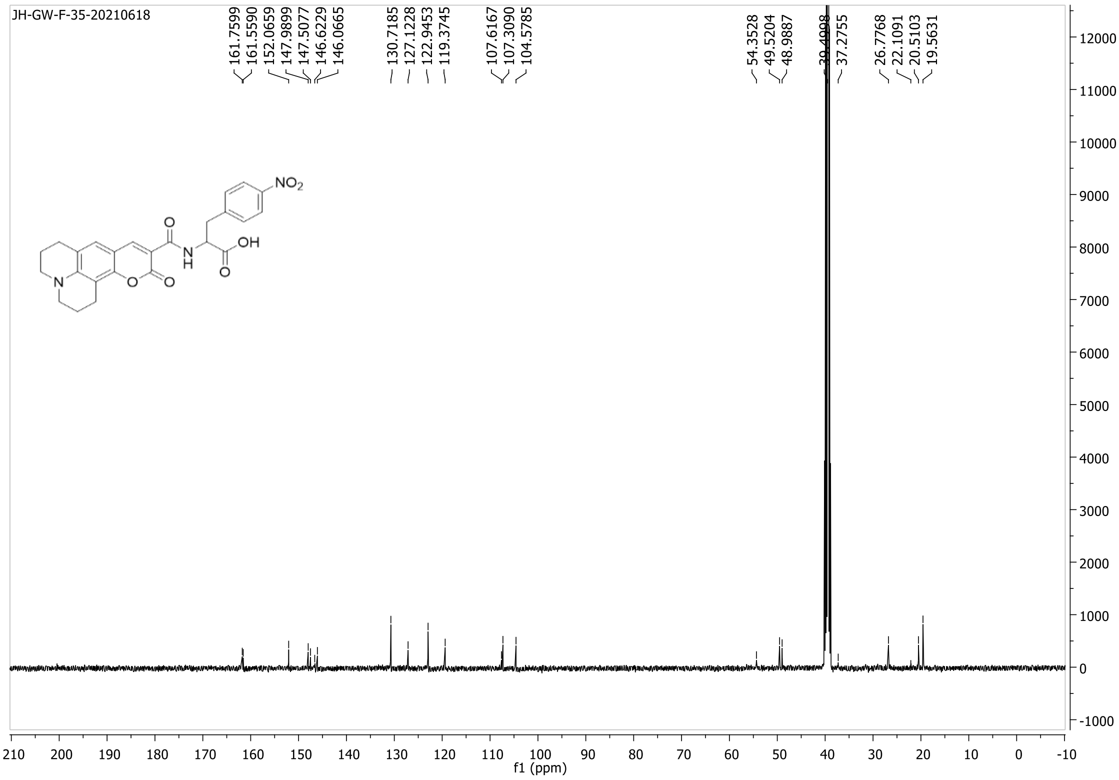
JH-GW-F-35-20210618
161.7599
161.5590
152.0659
147.9899
147.5077
146.6229
146.0665
130.7185
127.1228
122.9453
119.3745
107.6167
107.3090
104.5785
54.3528
49.5204
48.9887
37.2755
26.7768
22.1091
20.5103
19.5631
NO2
O
N
H
OH
O
N
O
O
f1 (ppm)

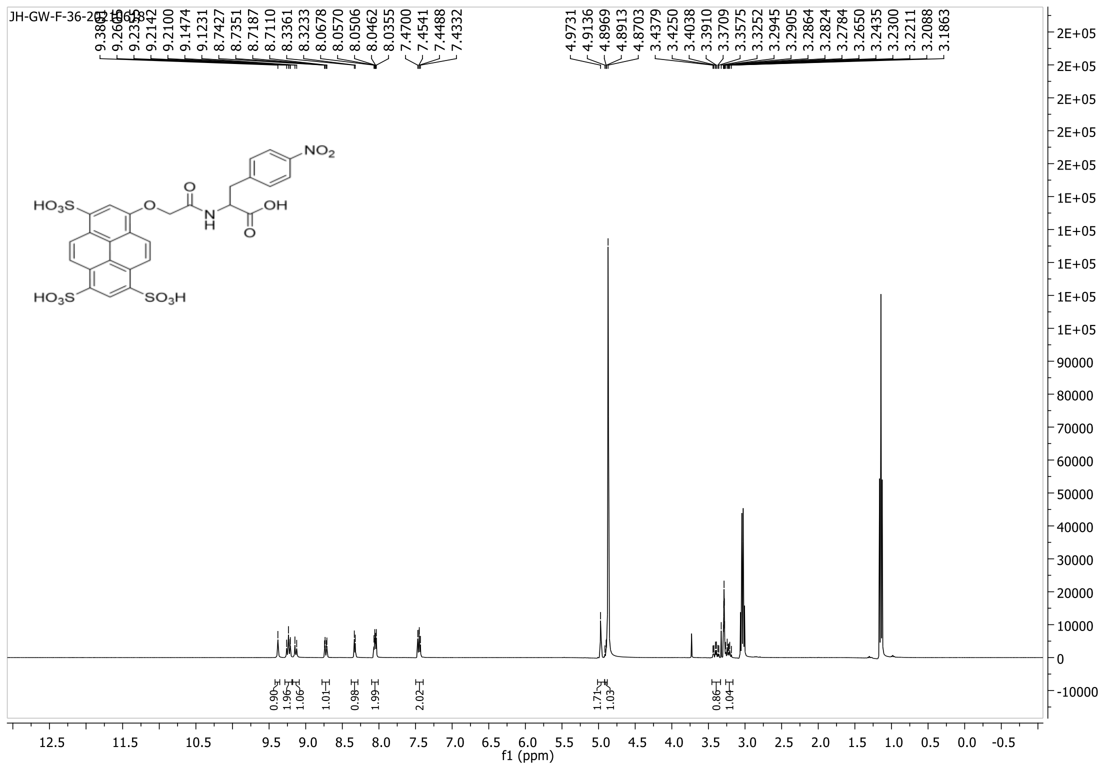
JH-GW-F-36-2021061
9.2142
9.2100
9.1474
9.1231
8.7427
8.7351
8.7187
8.7110
8.3361
8.3233
8.0678
8.0570
8.0506
8.0462
8.0355
7.4700
7.4541
7.4488
7.4332
4.9731
4.9136
4.8969
4.8913
4.8703
3.4379
3.4250
3.4038
3.3910
3.3709
3.3575
3.3252
3.2945
3.2905
3.2864
3.2824
3.2784
3.2650
3.2435
3.2300
3.2211
3.2088
3.1863
NO_2
HO_3S
O
N
H
OH
SO_3H
0.90
1.96
1.06
1.01
0.98
1.99
2.02
1.71
1.03
0.86
1.04
f1 (ppm)

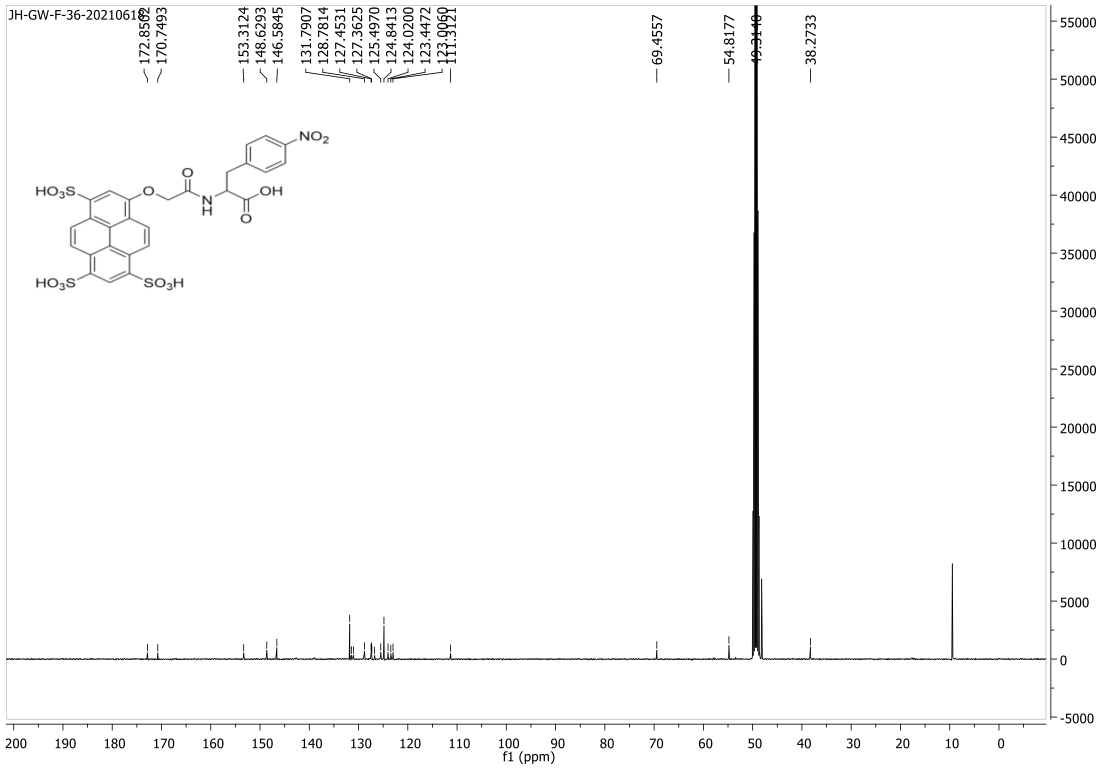
JH-GW-F-36-2021061
170.7493
153.3124
148.6293
146.5845
131.7907
128.7814
127.4531
127.3625
125.4970
124.8413
124.0200
123.4472
111.3121
69.4557
54.8177
38.2733
NO_2
HO_3S
O
N
H
OH
SO_3H
f1 (ppm)

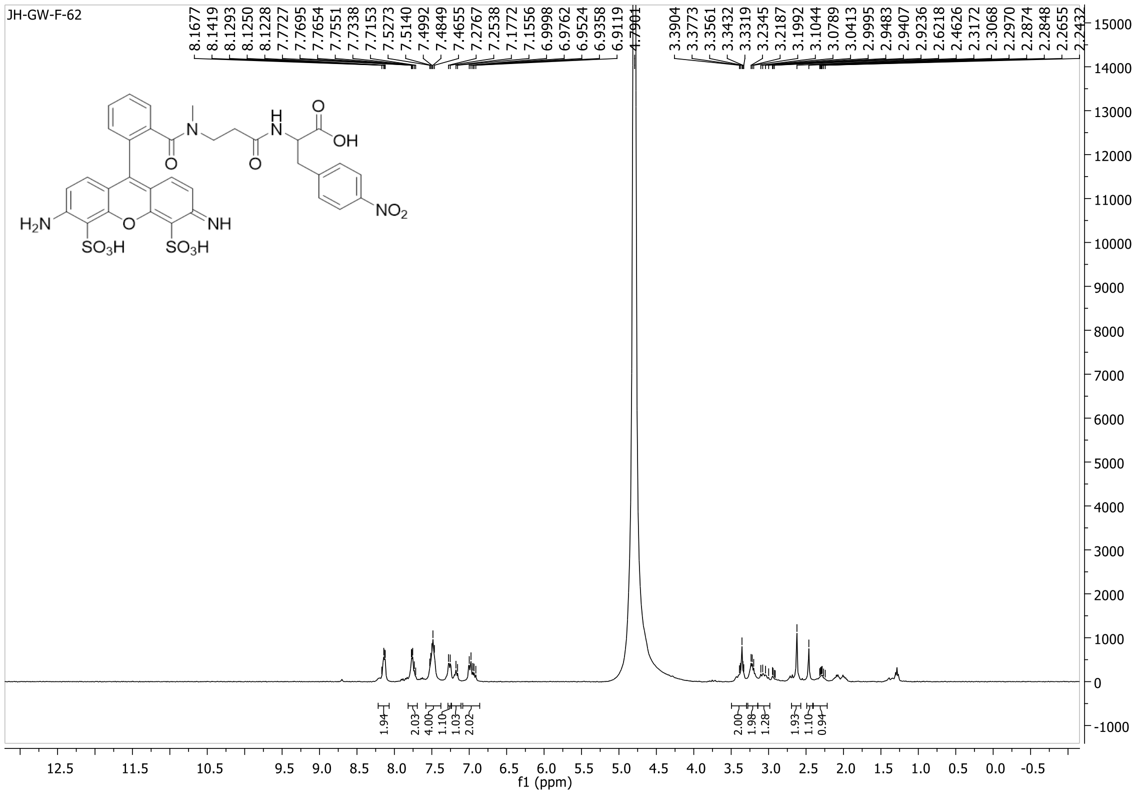
JH-GW-F-62
8.1677
8.1419
8.1293
8.1250
8.1228
7.7727
7.7695
7.7654
7.7551
7.7338
7.7153
7.5273
7.5140
7.4992
7.4849
7.4655
7.2767
7.2538
7.1772
7.1556
6.9998
6.9762
6.9524
6.9358
6.9119
4.7901
3.3904
3.3773
3.3561
3.3432
3.3319
3.2345
3.2187
3.1992
3.1044
3.0789
3.0413
2.9995
2.9483
2.9407
2.9236
2.6218
2.4626
2.3172
2.3068
2.2970
2.2874
2.2848
2.2655
2.2432
H2N
NH
O
SO3H
NO2
OH
f1 (ppm)

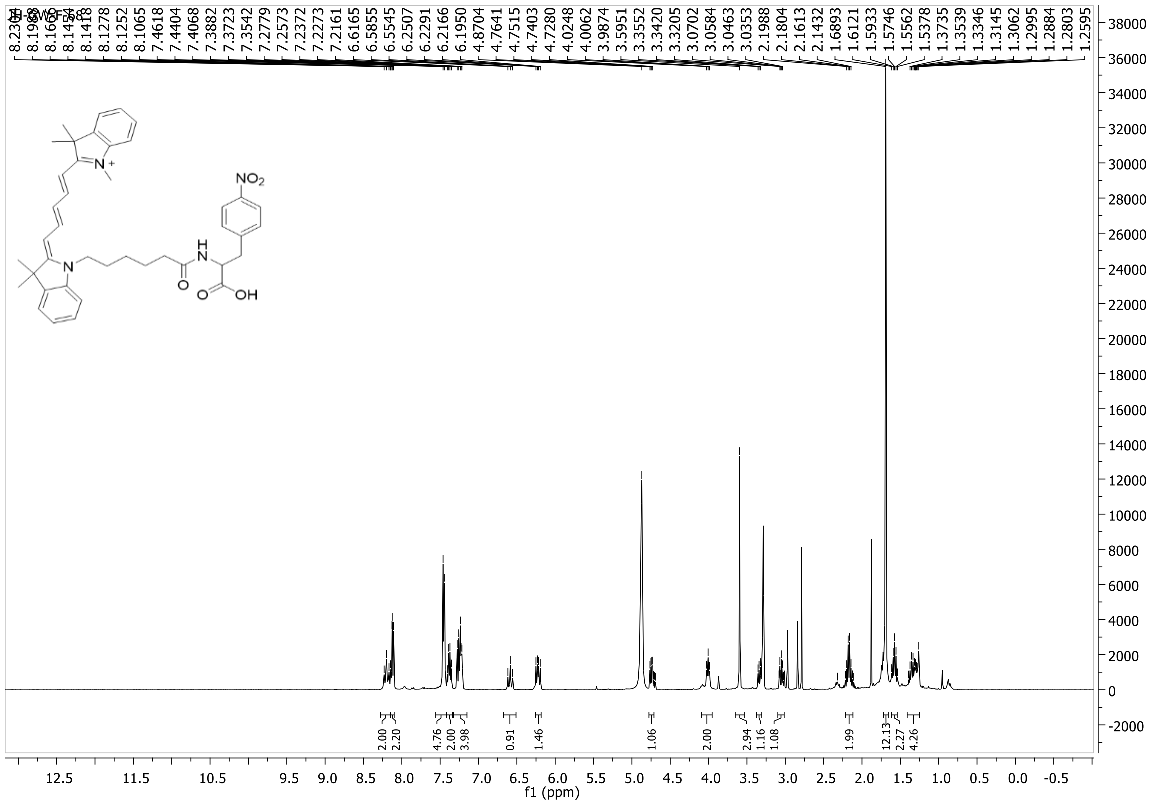
8.1418
8.1278
8.1252
8.1065
7.4618
7.4404
7.4068
7.3882
7.3723
7.3542
7.2779
7.2573
7.2372
7.2273
7.2161
6.6165
6.5855
6.5545
6.2507
6.2291
6.2166
6.1950
4.8704
4.7641
4.7515
4.7403
4.7280
4.0248
4.0062
3.9874
3.5951
3.3552
3.3420
3.3205
3.0702
3.0584
3.0463
3.0353
2.1988
2.1804
2.1613
2.1432
1.6893
1.6121
1.5933
1.5746
1.5562
1.5378
1.3735
1.3539
1.3346
1.3145
1.3062
1.2995
1.2884
1.2803
1.2595
NO2
N+
N
H
O
OH
f1 (ppm)

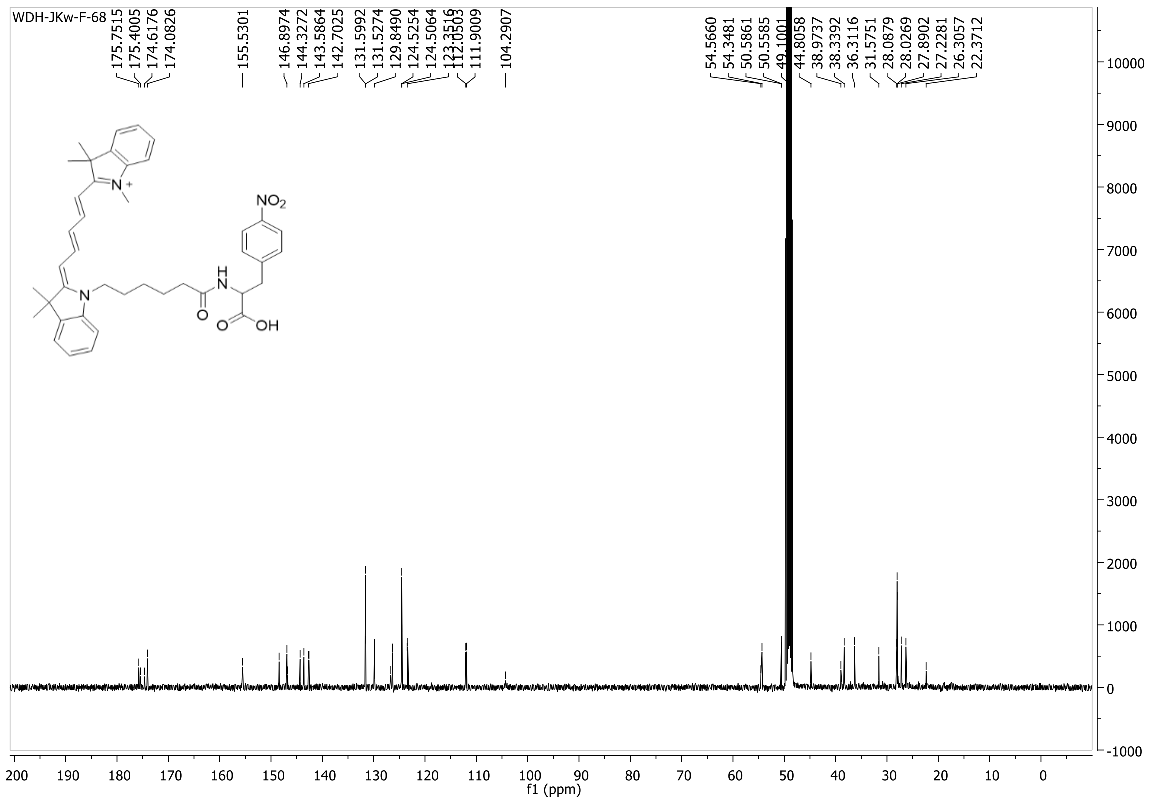

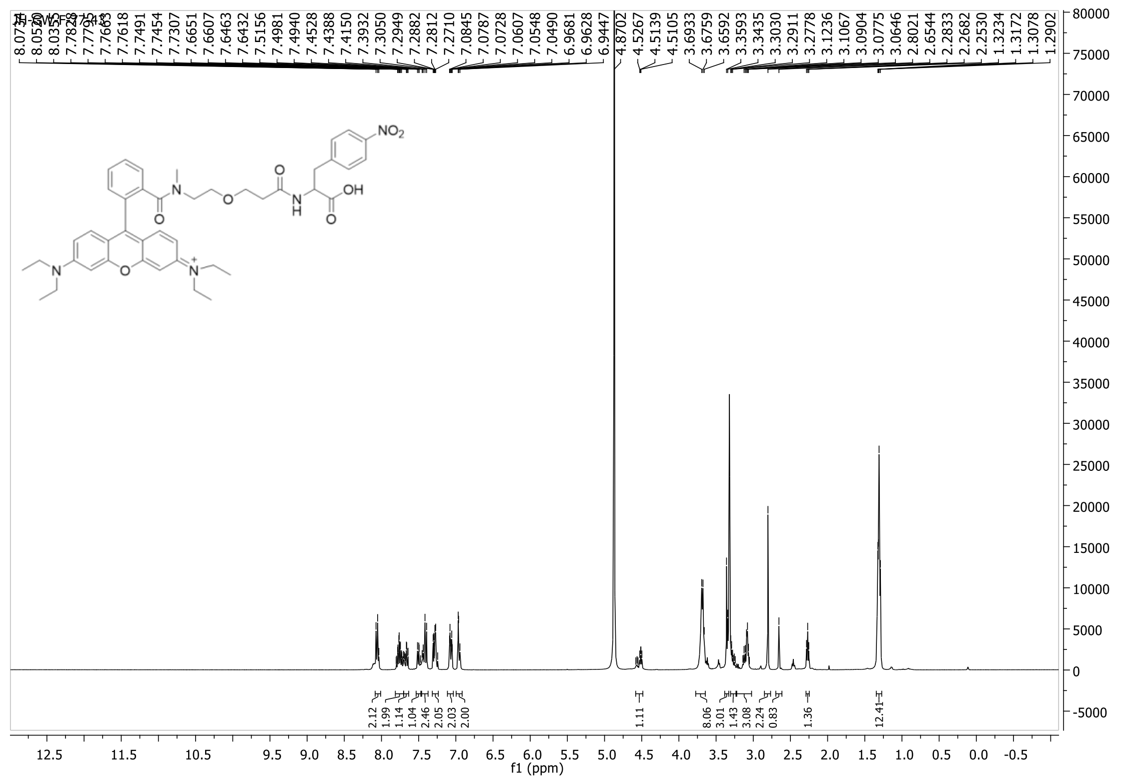

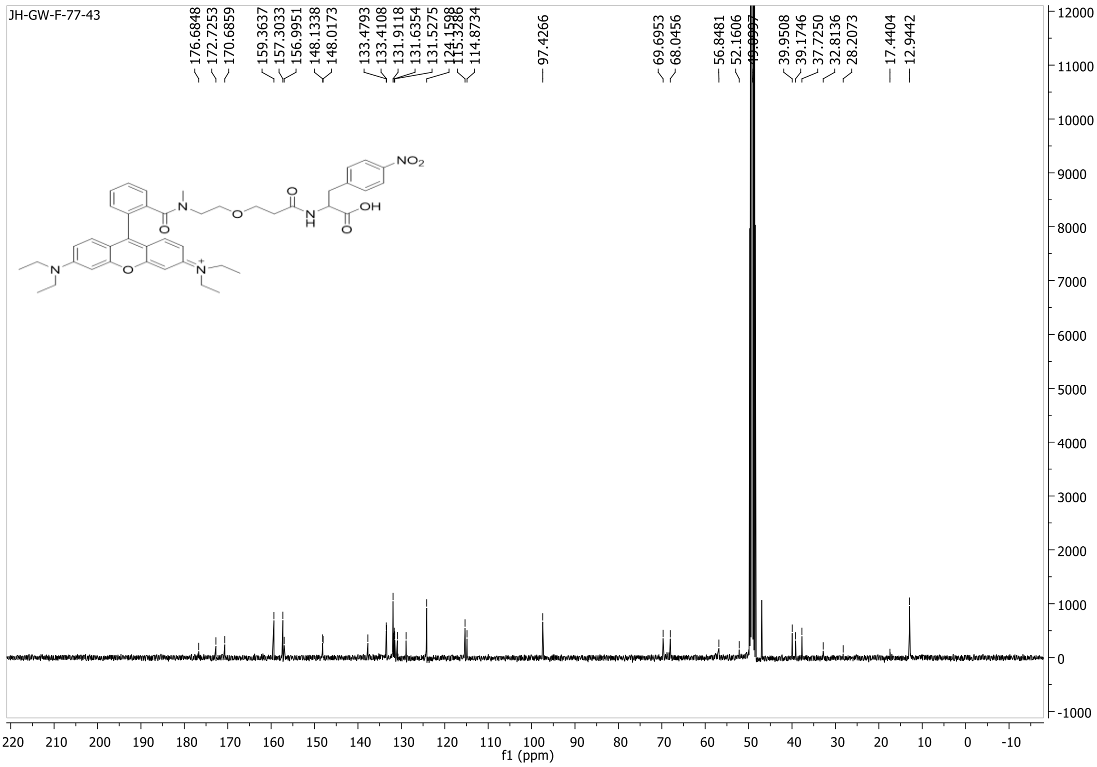
JH-GW-F-77-43
176.6848
172.7253
170.6859
159.3637
157.3033
156.9951
148.1338
148.0173
133.4793
133.4108
131.9118
131.6354
131.5275
114.8734
97.4266
69.6953
68.0456
56.8481
52.1606
39.9508
39.1746
37.7250
32.8136
28.2073
17.4404
12.9442
NO_2
OH
f1 (ppm)

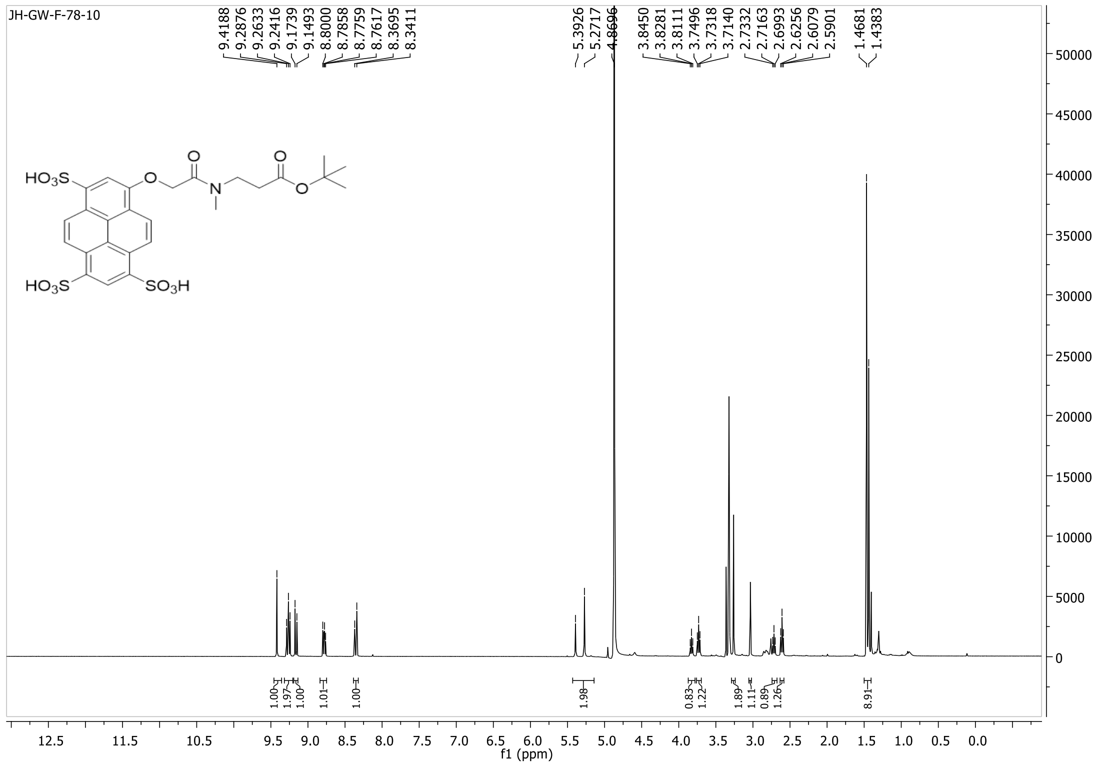
JH-GW-F-78-10
9.4188
9.2876
9.2633
9.2416
9.1739
9.1493
8.8000
8.7858
8.7759
8.7617
8.3695
8.3411
5.3926
5.2717
3.8450
3.8281
3.8111
3.7496
3.7318
3.7140
2.7332
2.7163
2.6993
2.6256
2.6079
2.5901
1.4681
1.4383
HO_3S
HO_3S
SO_3H
f1 (ppm)

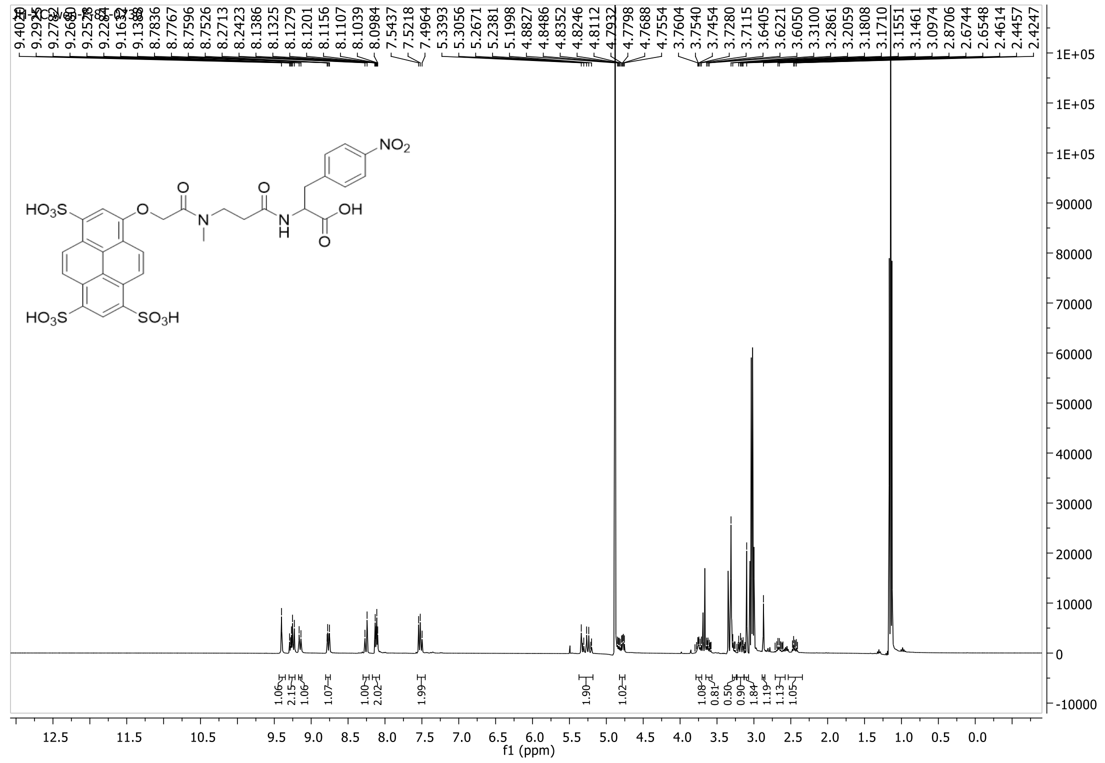
HO3S
HO3S
SO3H
NO2
OH
N
H
f1 (ppm)

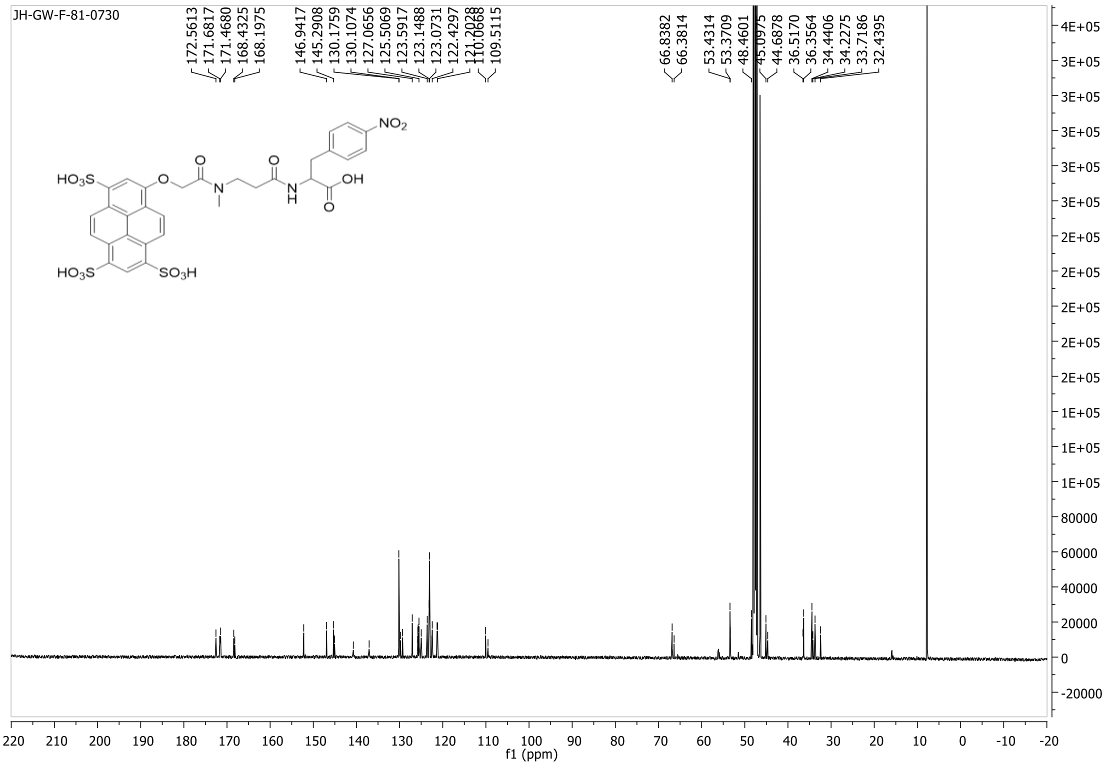
JH-GW-F-81-0730
172.5613
171.6817
171.4680
168.4325
168.1975
146.9417
145.2908
130.1759
130.1074
127.0656
125.5069
123.5917
123.1488
123.0731
122.4297
121.2028
110.0668
109.5115
66.8382
66.3814
53.4314
53.3709
48.4601
45.0975
44.6878
36.5170
36.3564
34.4406
34.2275
33.7186
32.4395
HO3S
HO3S
SO3H
NO2
OH
N
H
f1 (ppm)

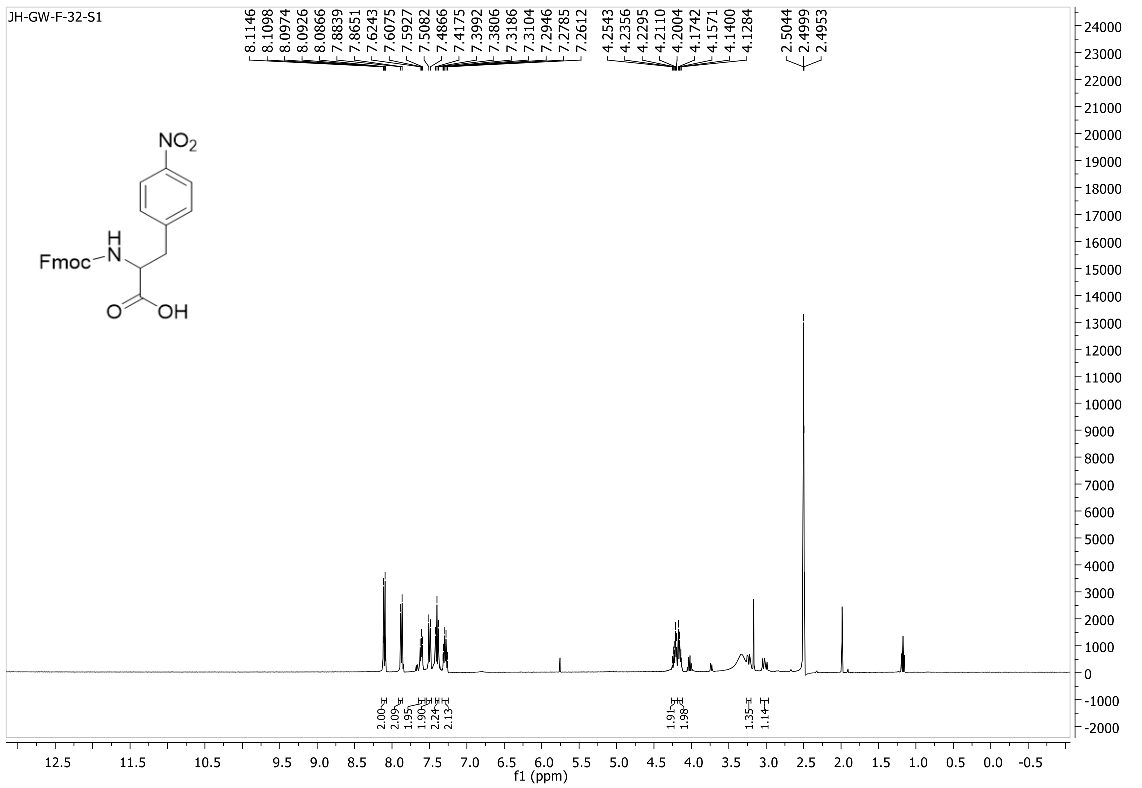

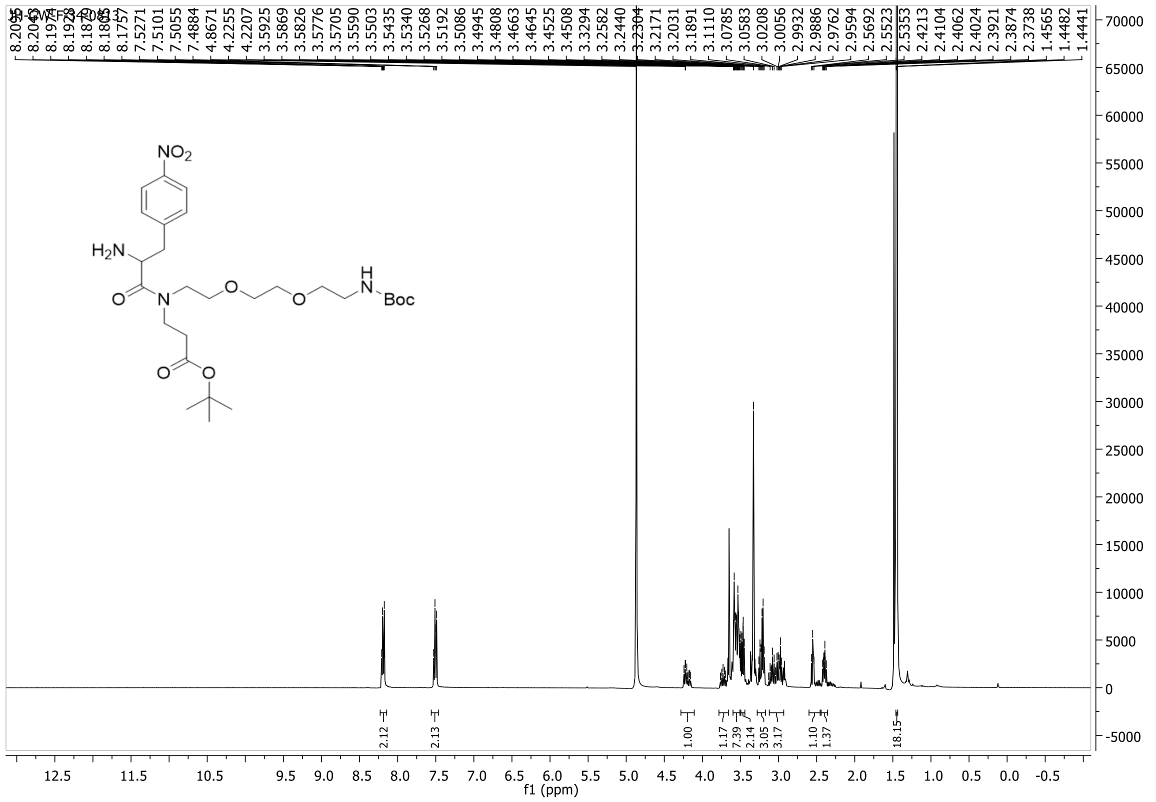

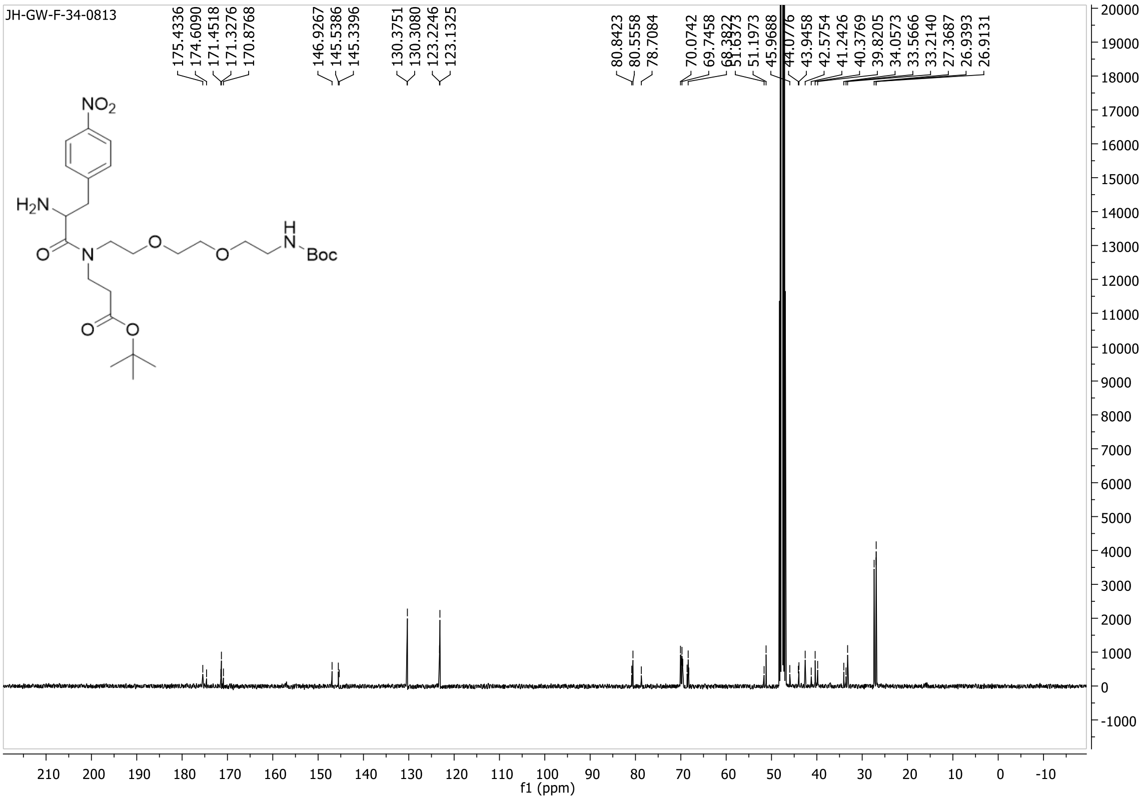

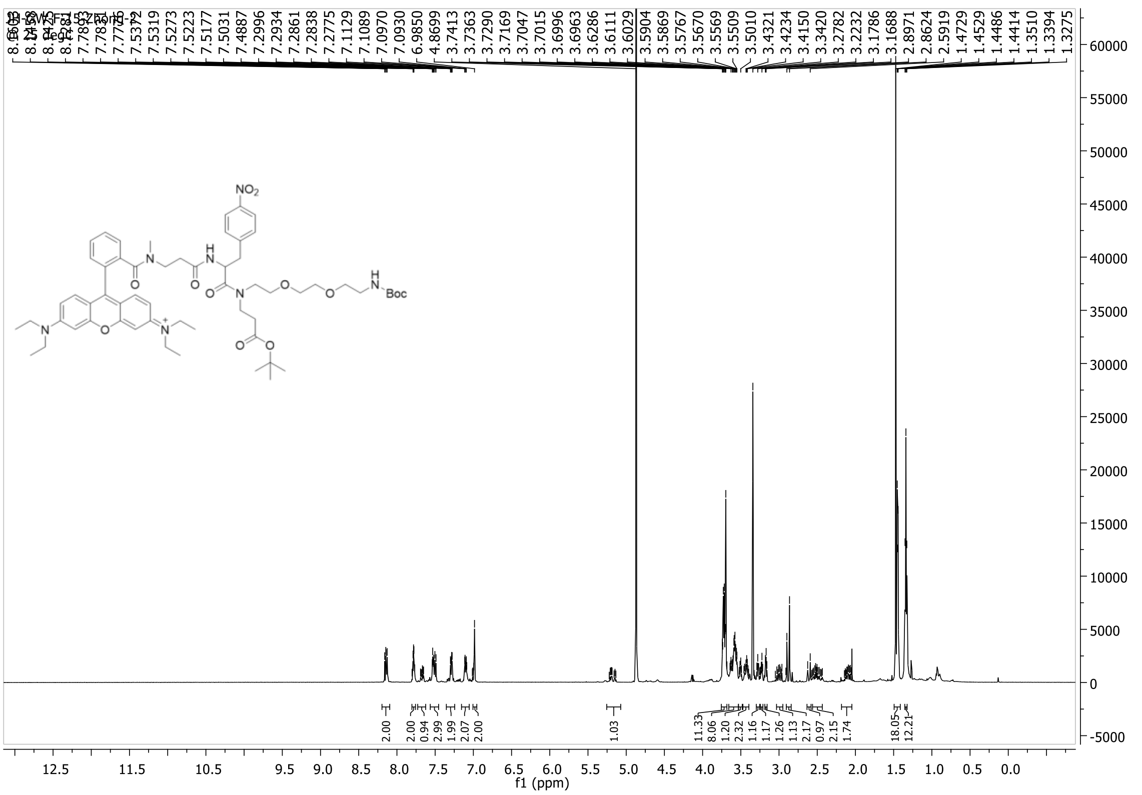

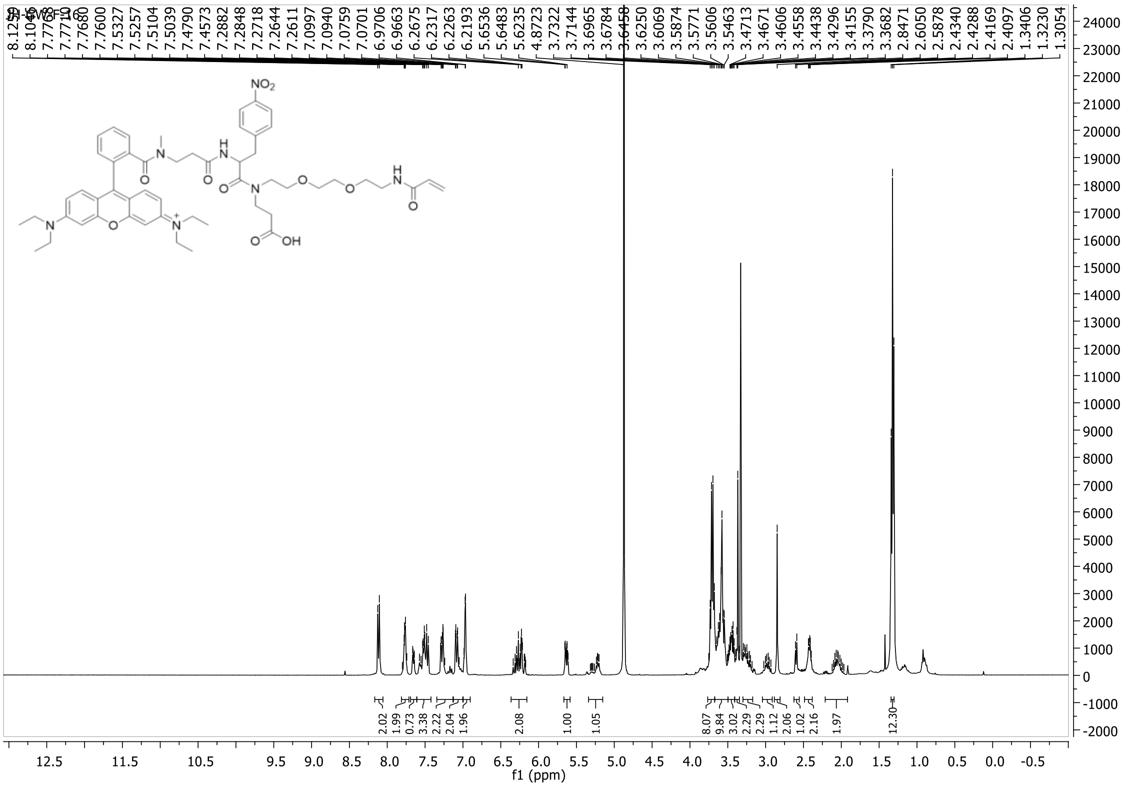
JH-GW-F-16
7.7710
7.7680
7.7600
7.5327
7.5257
7.5104
7.5039
7.4790
7.4573
7.2882
7.2848
7.2718
7.2644
7.2611
7.0997
7.0940
7.0759
7.0701
6.9706
6.9663
6.2675
6.2317
6.2263
6.2193
5.6536
5.6483
5.6235
4.8723
3.7322
3.7144
3.6965
3.6784
3.6250
3.6069
3.5874
3.5771
3.5606
3.5463
3.4713
3.4671
3.4606
3.4558
3.4438
3.4296
3.4155
3.3790
3.3682
2.8471
2.6050
2.5878
2.4340
2.4288
2.4169
2.4097
1.3406
1.3230
1.3054
NO2
N
H
O
OH
2.02
1.99
0.73
3.38
2.22
2.04
1.96
2.08
1.00
1.05
8.07
9.84
3.02
2.29
2.29
1.12
2.06
1.02
2.16
1.97
12.30
f1 (ppm)

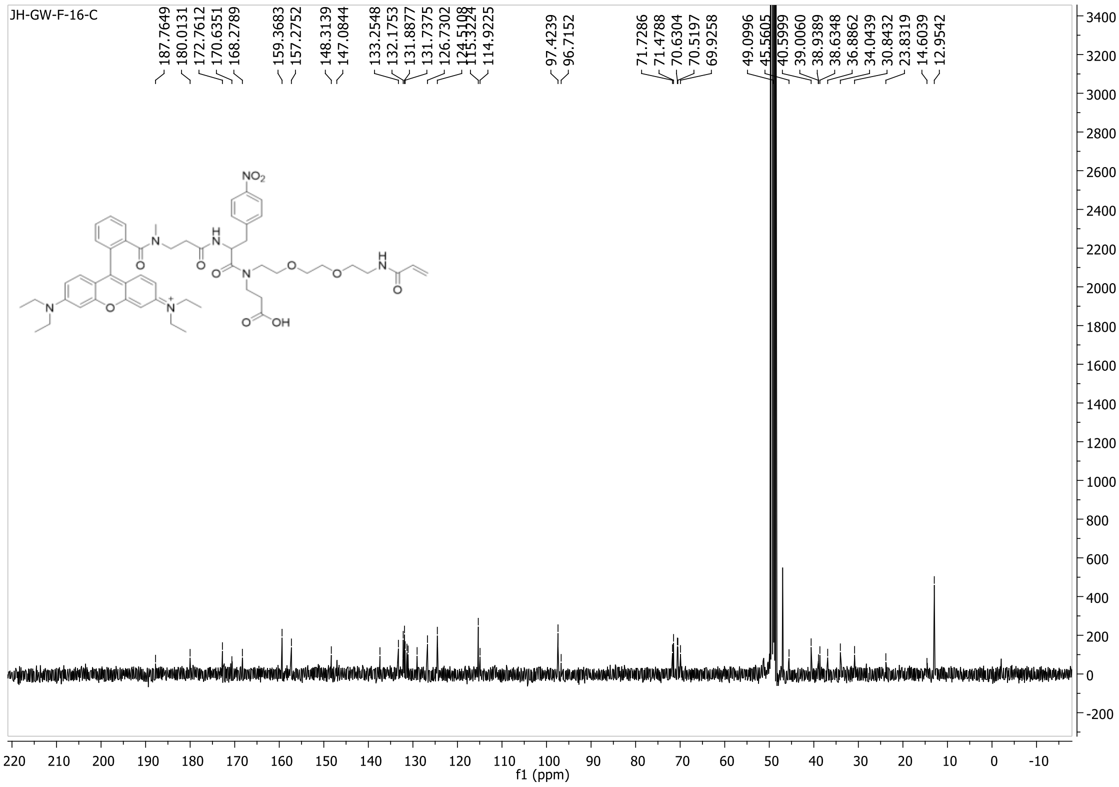
JH-GW-F-16-C
187.7649
180.0131
172.7612
170.6351
168.2789
159.3683
157.2752
148.3139
147.0844
133.2548
132.1753
131.8877
131.7375
126.7302
124.5108
115.3224
114.9225
97.4239
96.7152
71.7286
71.4788
70.6304
70.5197
69.9258
49.0996
45.5605
40.5999
39.0060
38.9389
38.6348
36.8862
34.0439
30.8432
23.8319
14.6039
12.9542
NO2
N
H
O
OH
f1 (ppm)

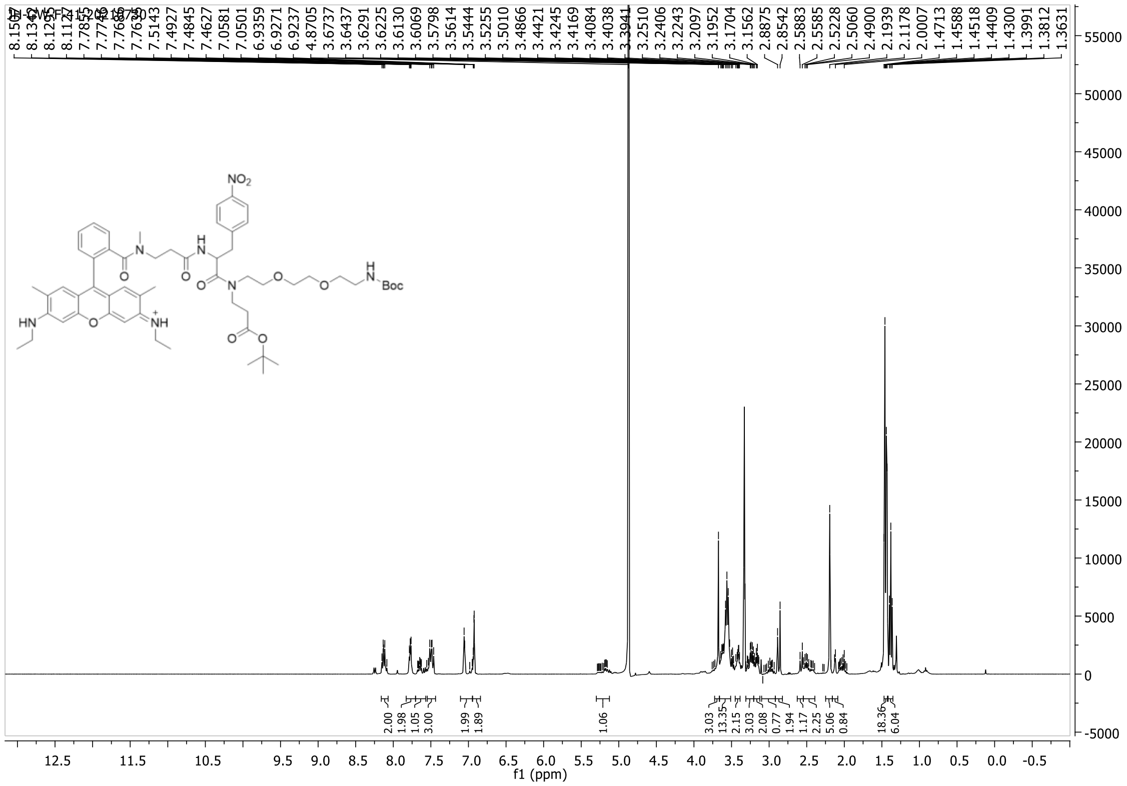
NO2
HN
NH
Boc
f1 (ppm)

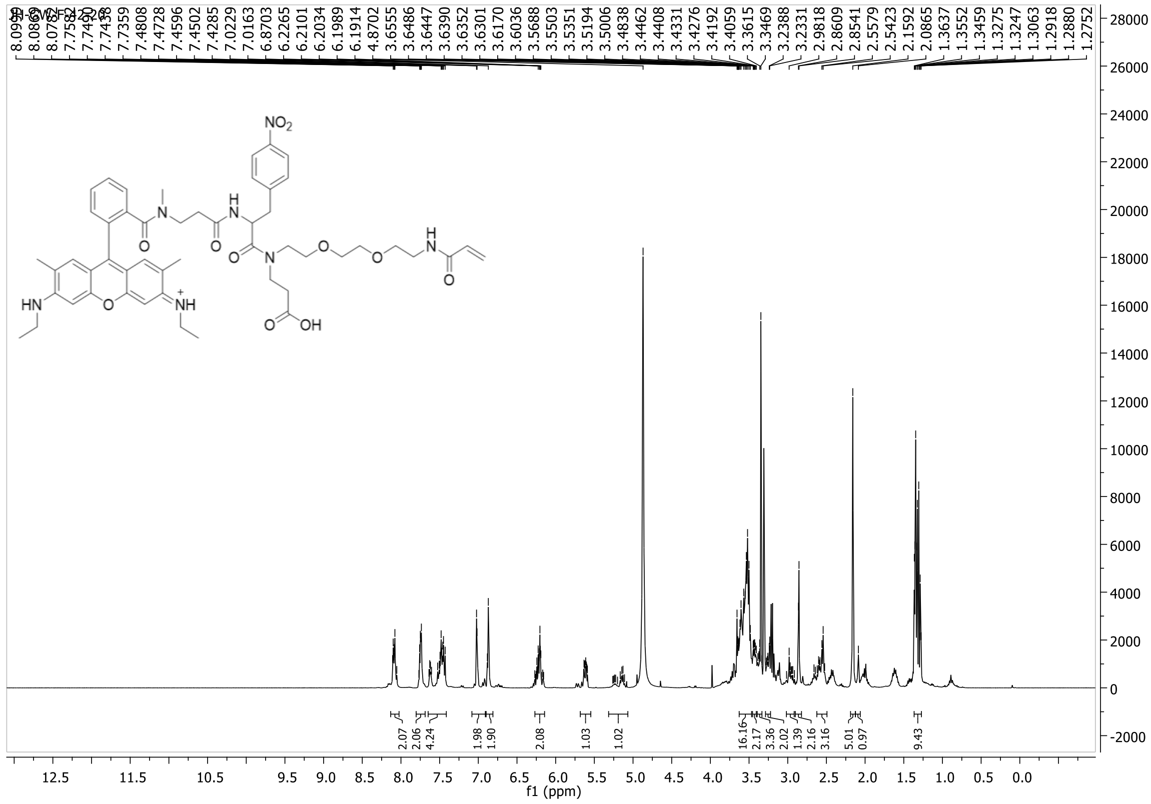
NO2
HN
NH
OH
f1 (ppm)

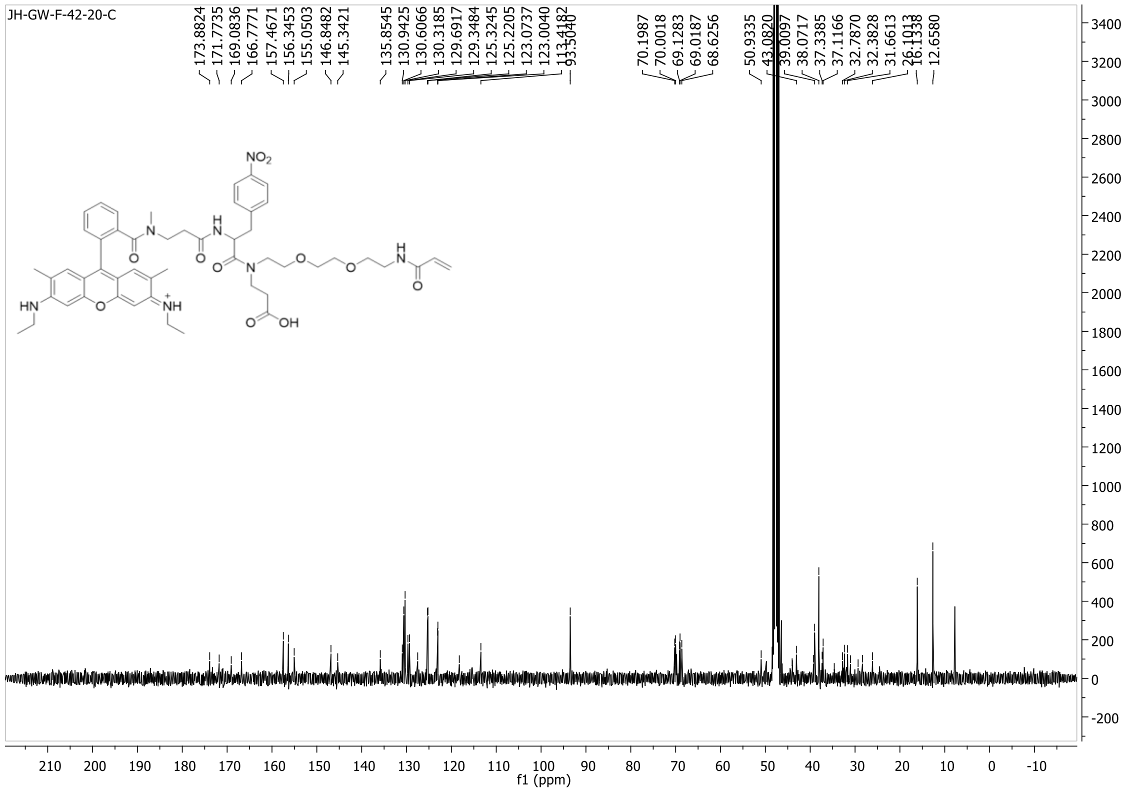
JH-GW-F-42-20-C
f1 (ppm)

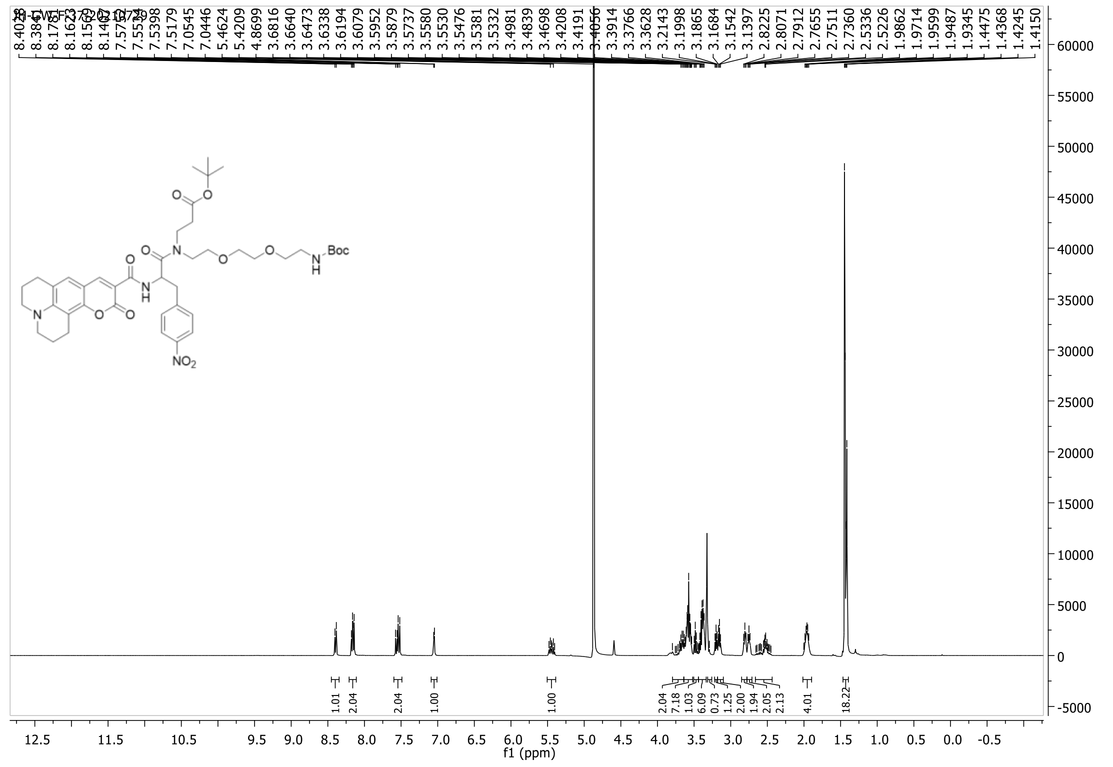
f1 (ppm)

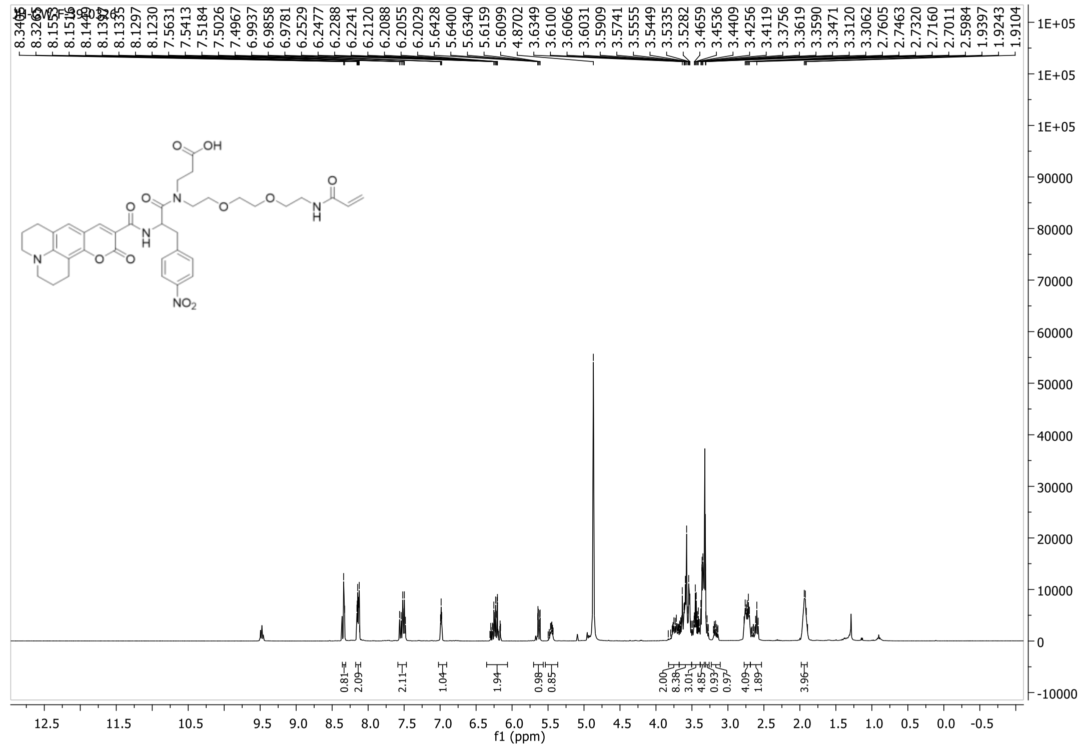

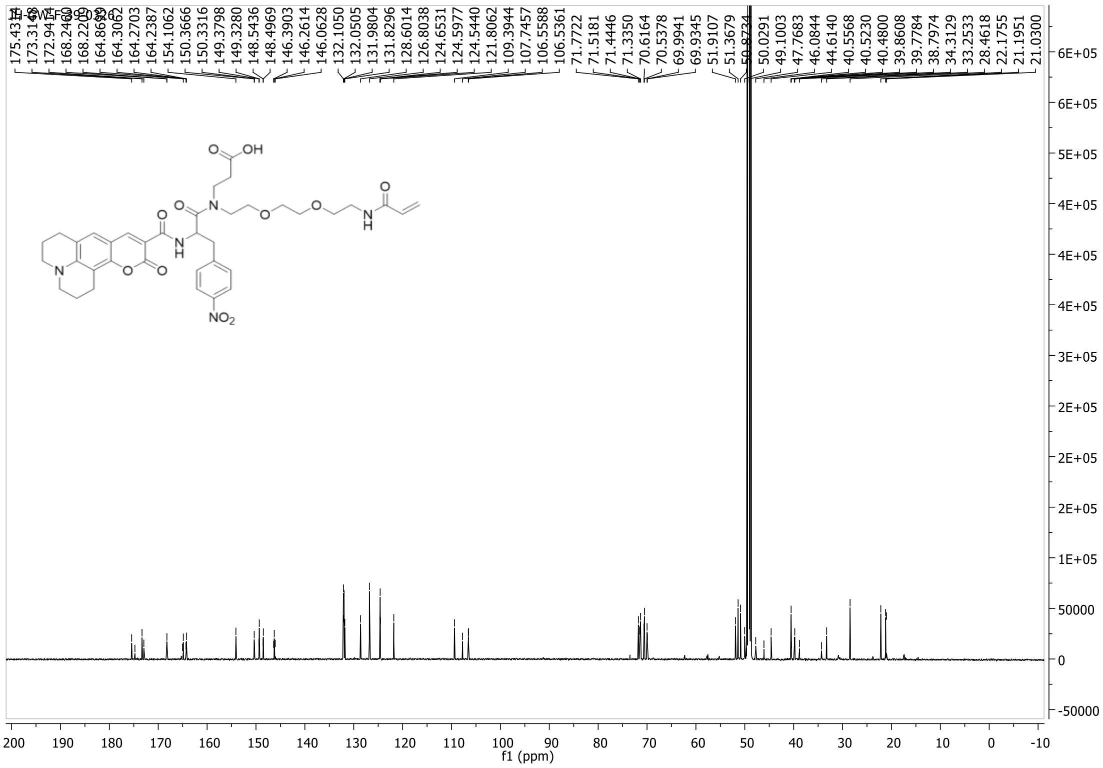

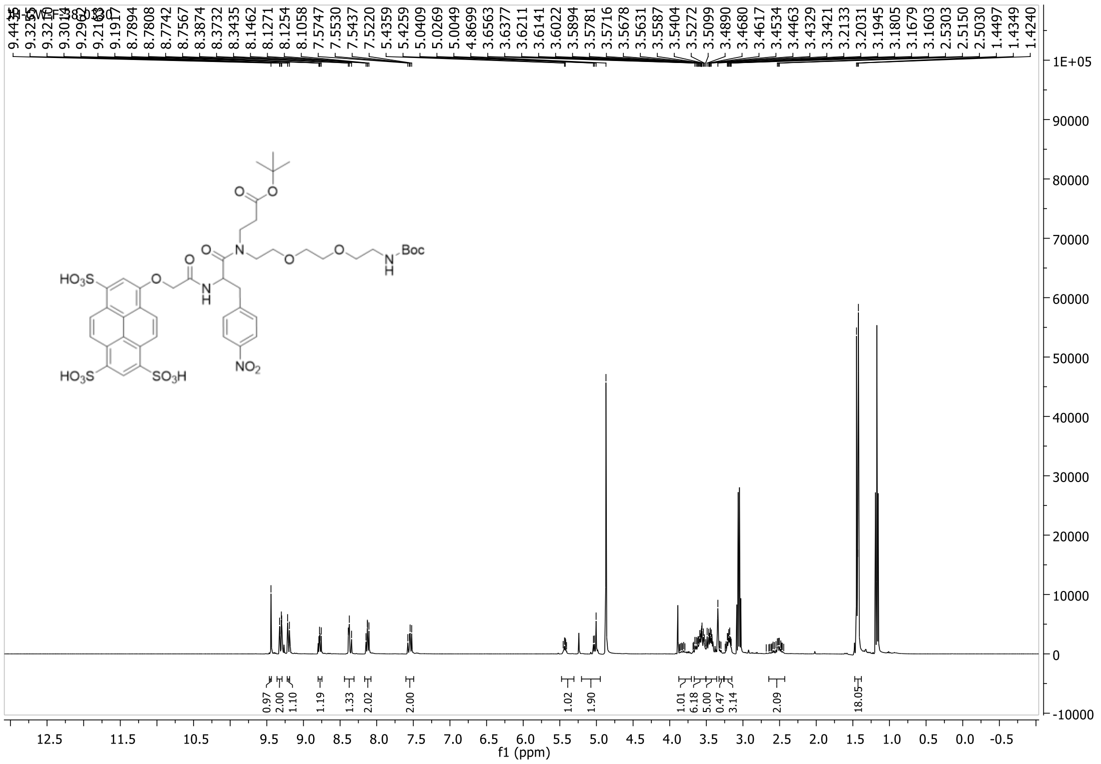

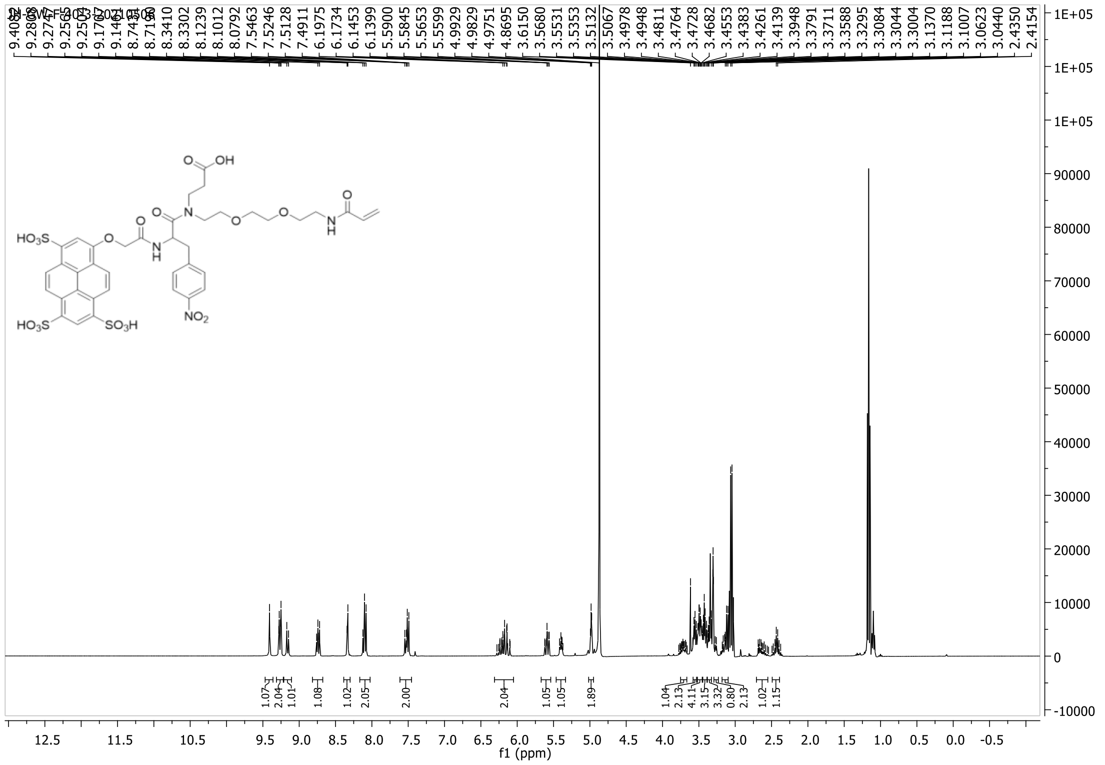

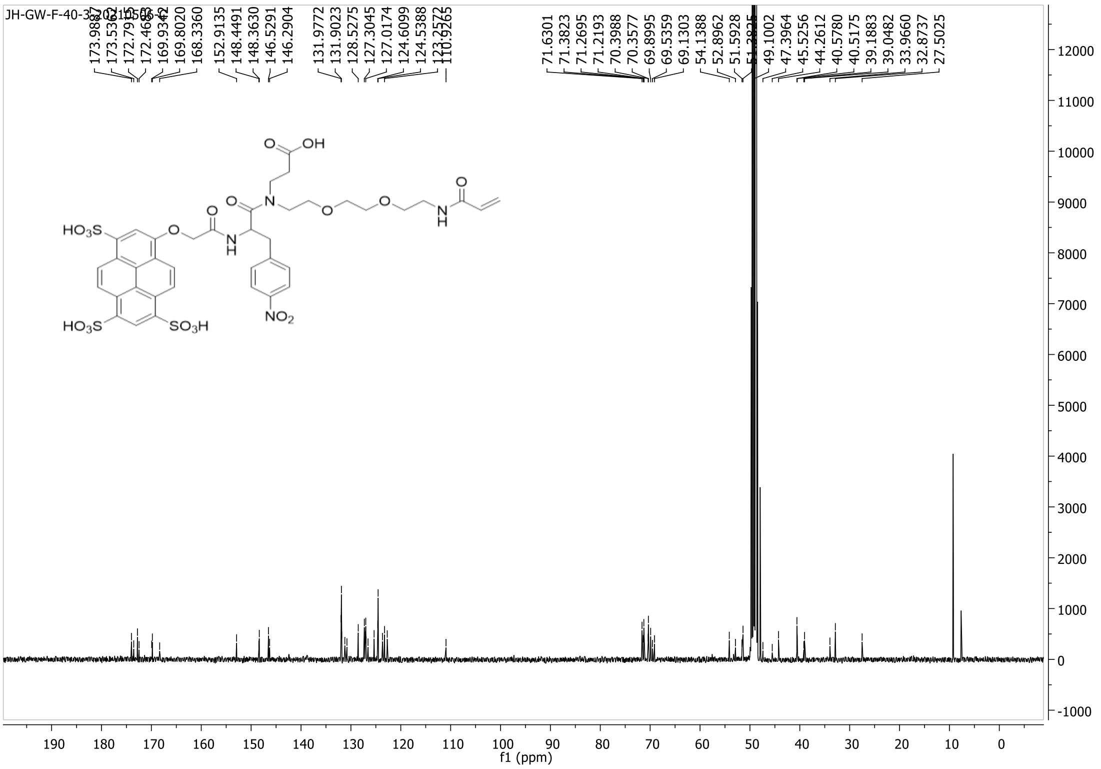

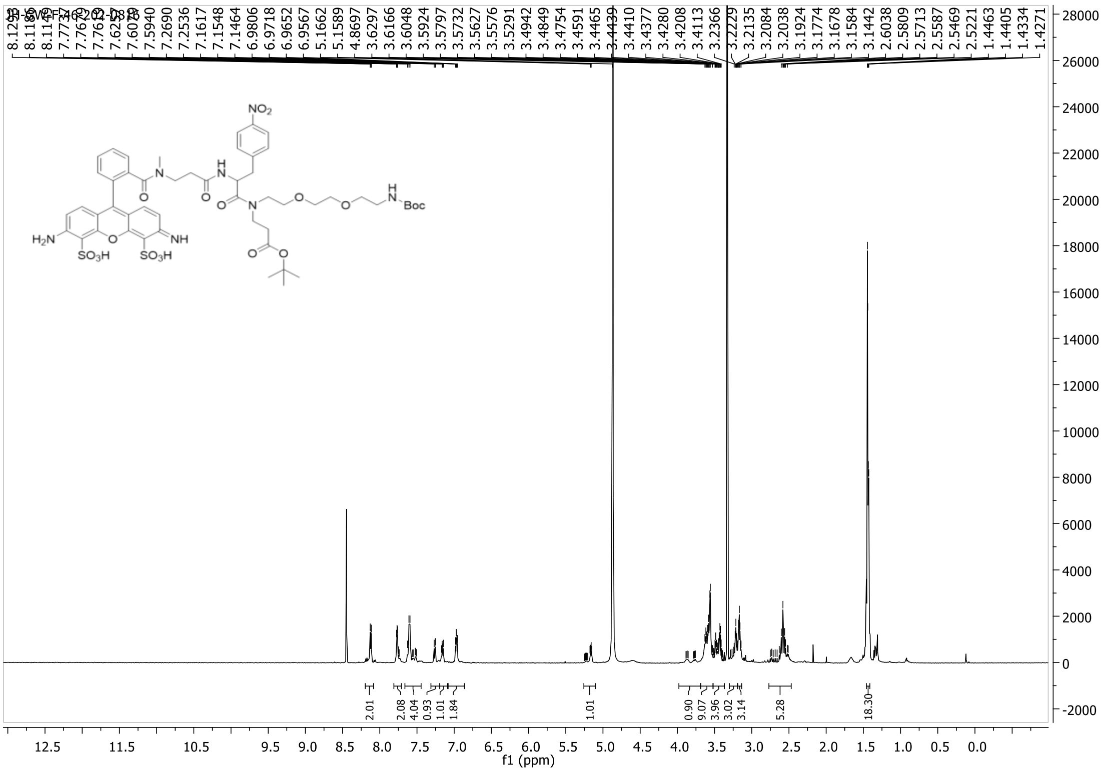

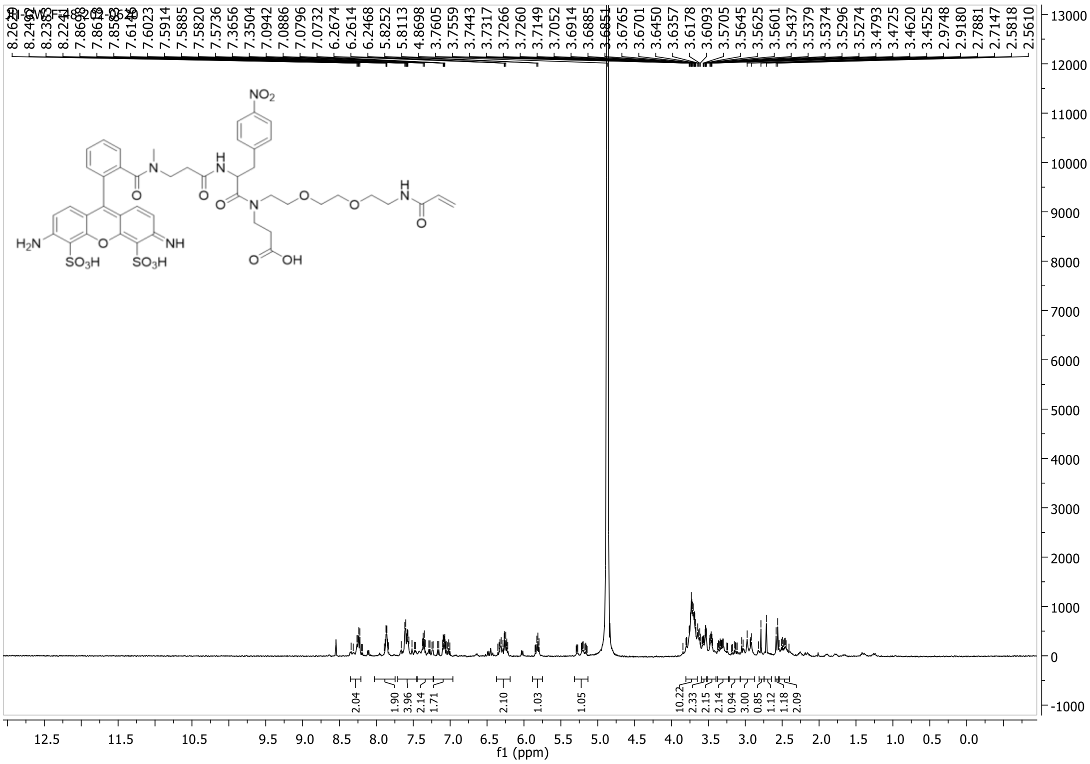
7.5914
7.5885
7.5820
7.5736
7.3656
7.3504
7.0942
7.0886
7.0796
7.0732
6.2674
6.2614
6.2468
5.8252
5.8113
4.8698
3.7605
3.7559
3.7443
3.7317
3.7266
3.7260
3.7149
3.7052
3.6914
3.6885
3.6765
3.6701
3.6450
3.6357
3.6178
3.6093
3.5705
3.5645
3.5625
3.5601
3.5437
3.5379
3.5374
3.5296
3.5274
3.4793
3.4725
3.4620
3.4525
2.9748
2.9180
2.7881
2.7147
2.5818
2.5610
NO_2
H_2N
NH
SO_3H
OH
2.04
1.90
3.96
2.14
1.71
2.10
1.03
1.05
10.22
2.33
2.15
2.14
0.94
3.00
0.85
1.12
1.18
2.09
f1 (ppm)

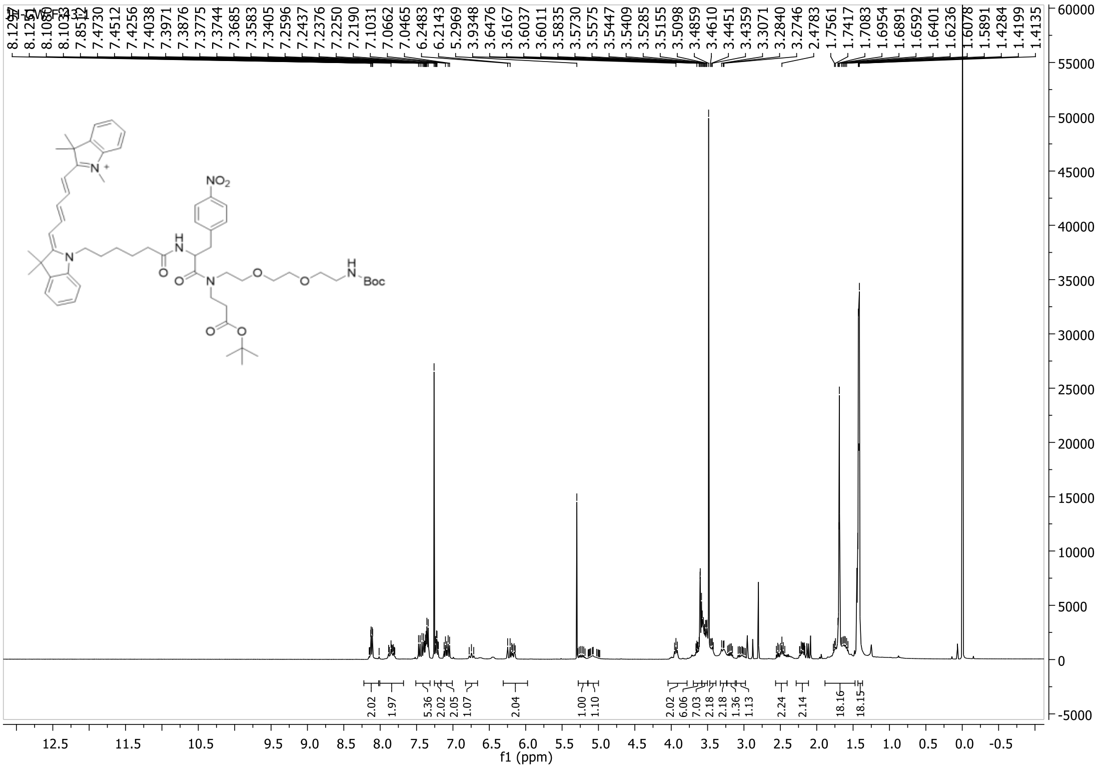
8.1033
7.8512
7.4730
7.4512
7.4256
7.4038
7.3971
7.3876
7.3775
7.3744
7.3685
7.3583
7.3405
7.2596
7.2437
7.2376
7.2250
7.2190
7.1031
7.0662
7.0465
6.2483
6.2143
5.2969
3.9348
3.6476
3.6167
3.6037
3.6011
3.5835
3.5730
3.5575
3.5447
3.5409
3.5285
3.5155
3.5098
3.4859
3.4610
3.4451
3.4359
3.3071
3.2840
3.2746
2.4783
1.7561
1.7417
1.7083
1.6954
1.6891
1.6592
1.6401
1.6236
1.6078
1.5891
1.4284
1.4199
1.4135
NO_2
Boc
2.02
1.97
5.36
2.02
2.05
1.07
2.04
1.00
1.10
2.02
6.06
7.03
2.18
2.18
1.36
1.13
2.24
2.14
18.16
18.15
f1 (ppm)

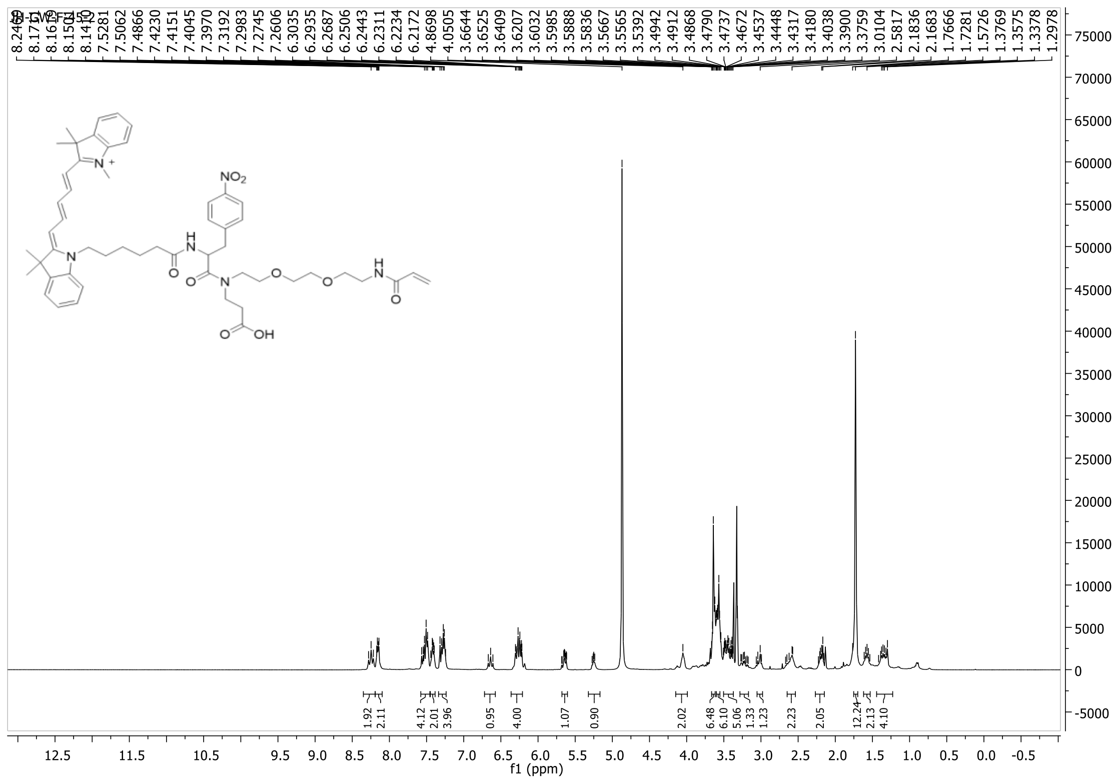

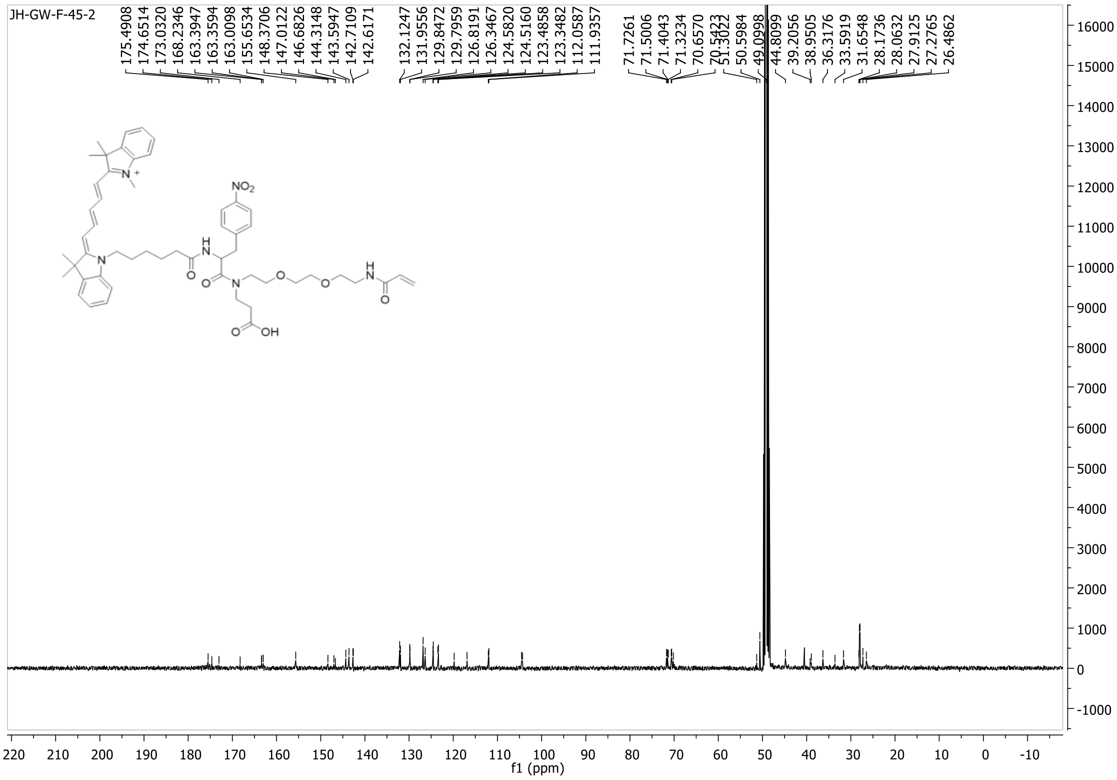
JH-GW-F-45-2

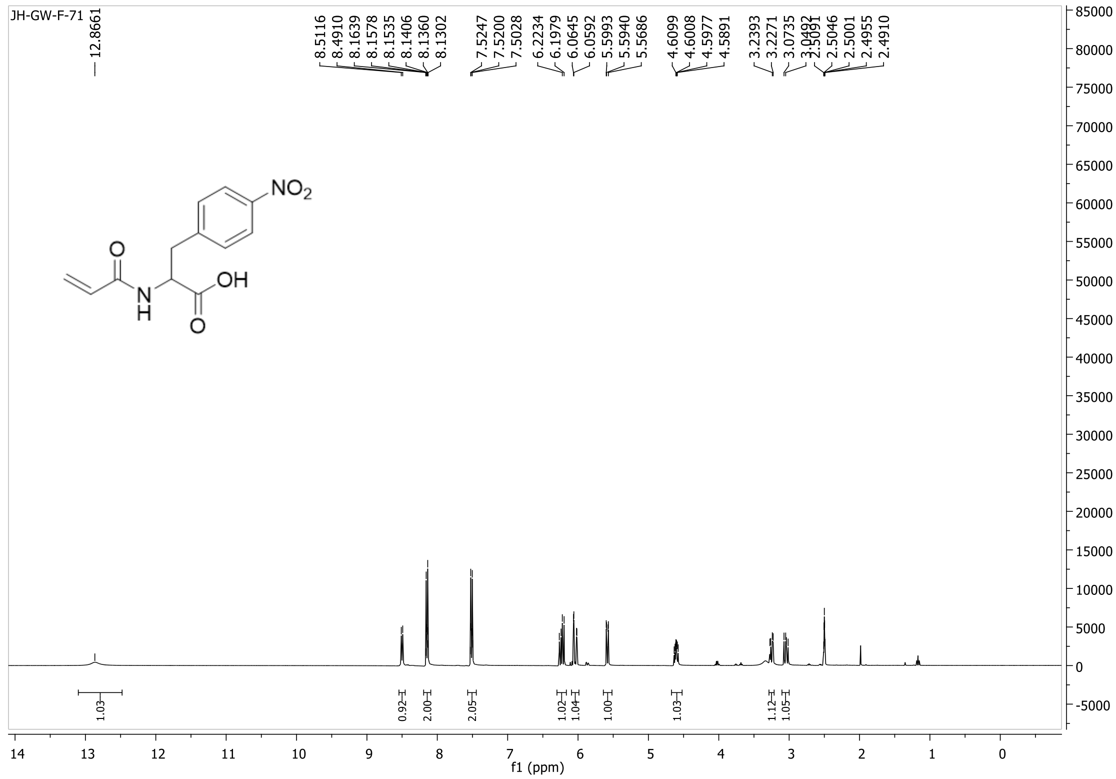
JH-GW-F-71
NO2
O
N
H
OH
O
f1 (ppm)

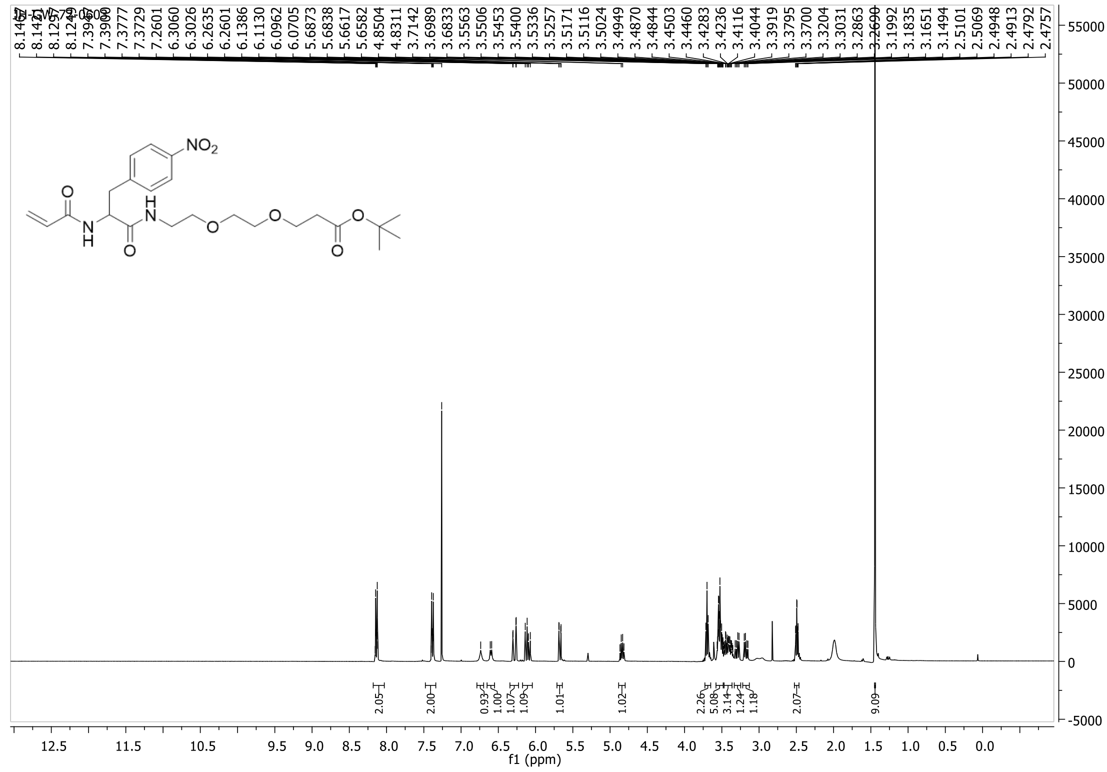
NO2
O
H
N
O
O
O
O
N
H
O
f1 (ppm)

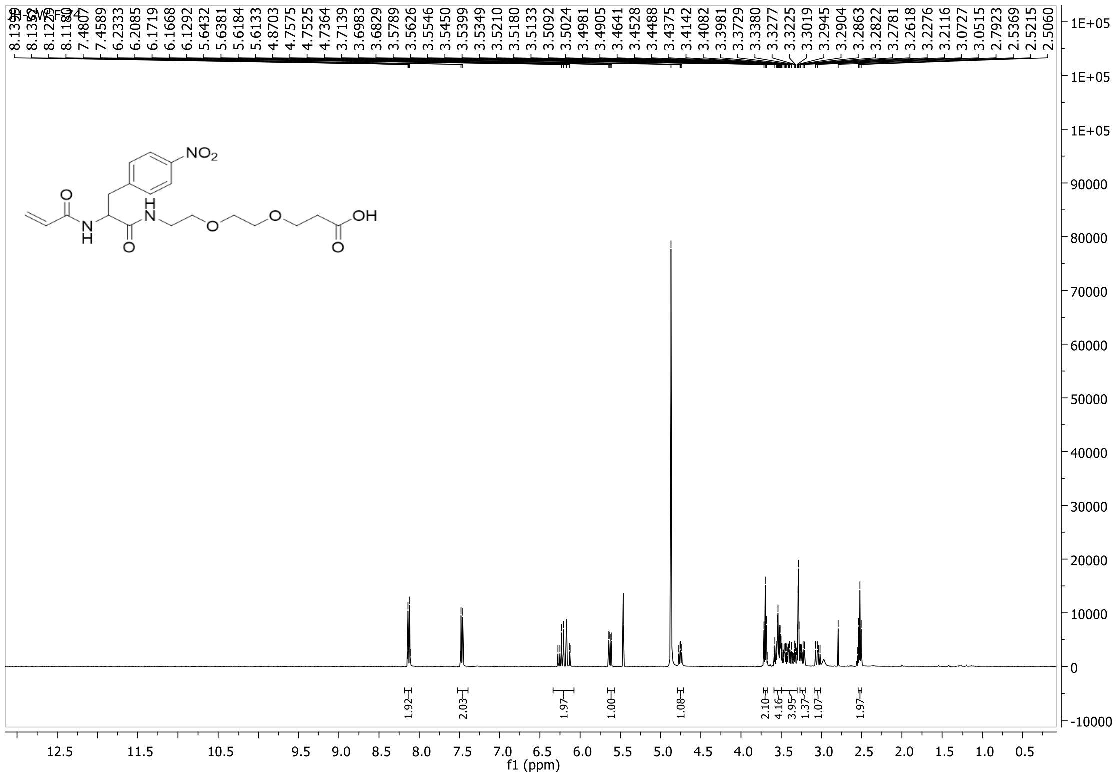

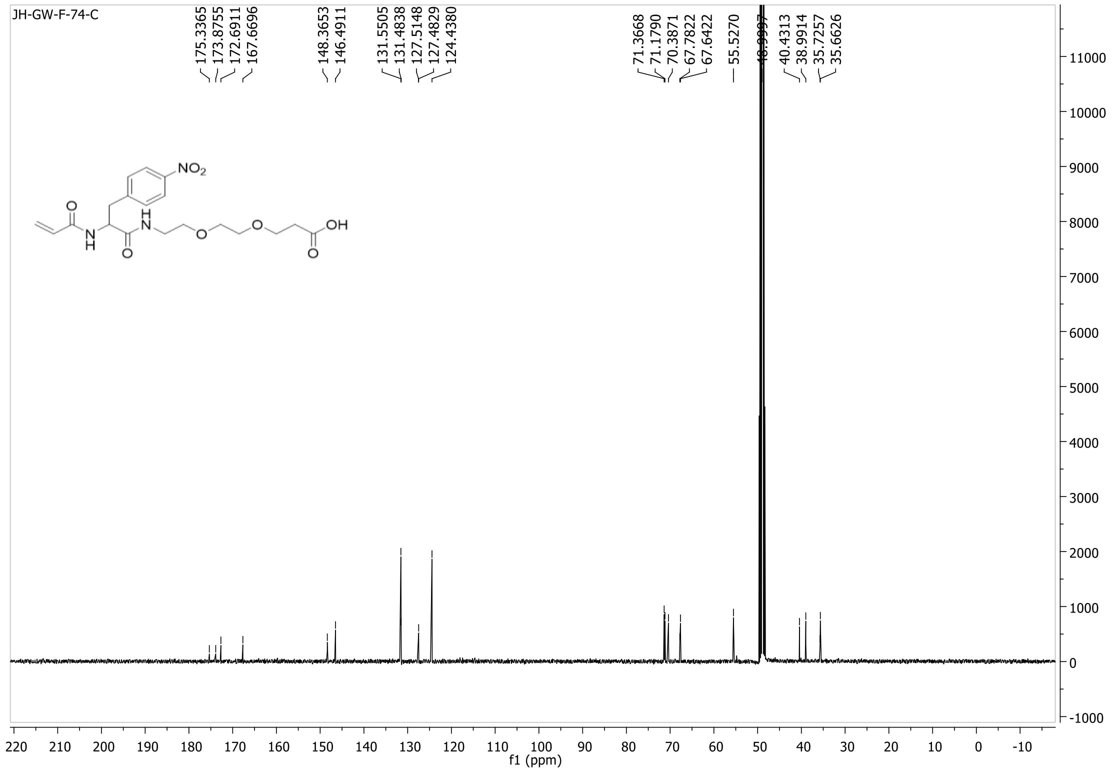

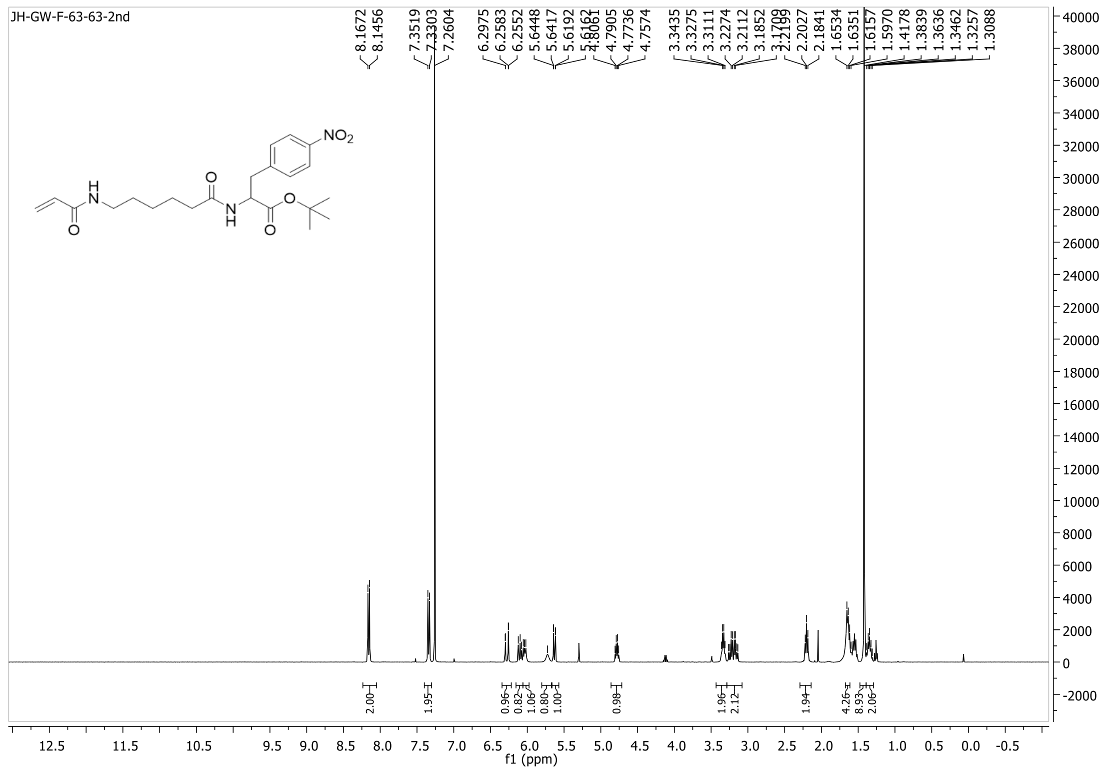

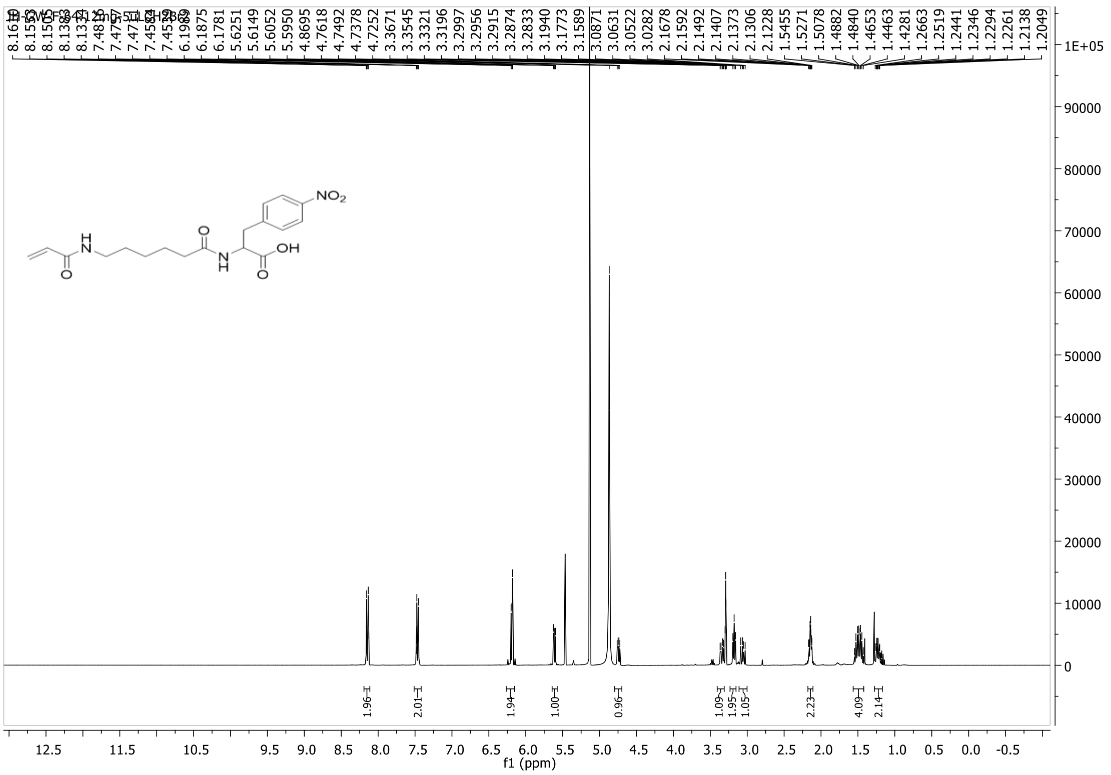

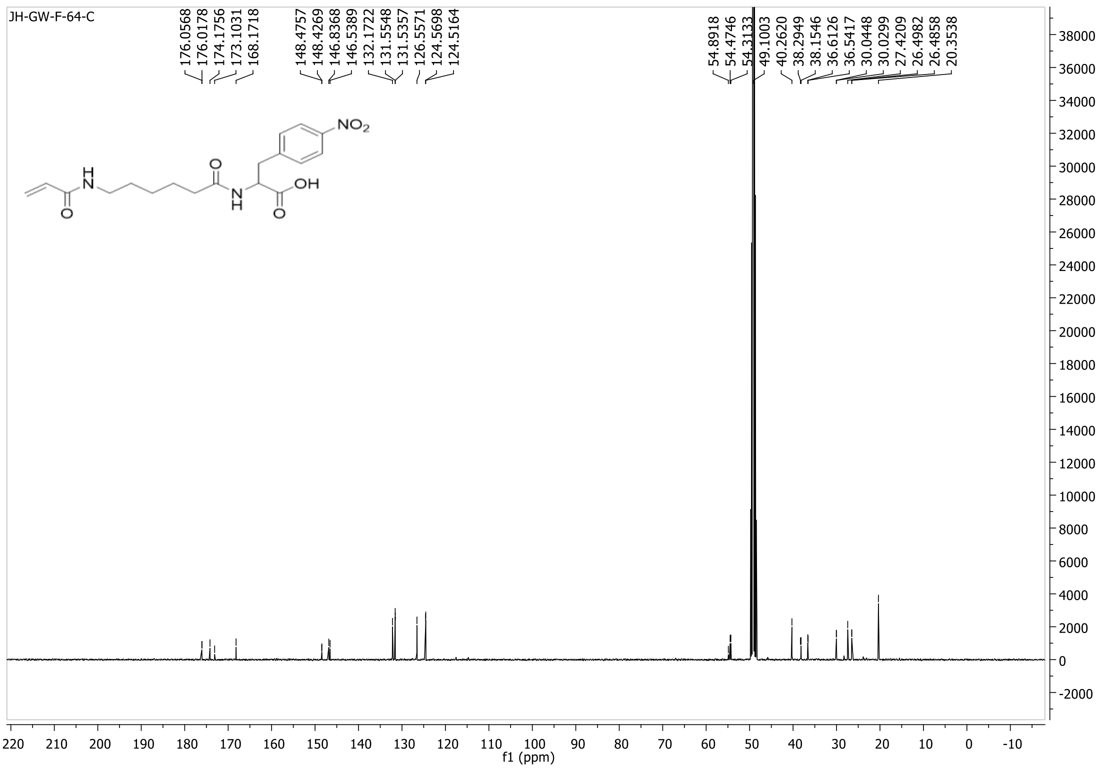

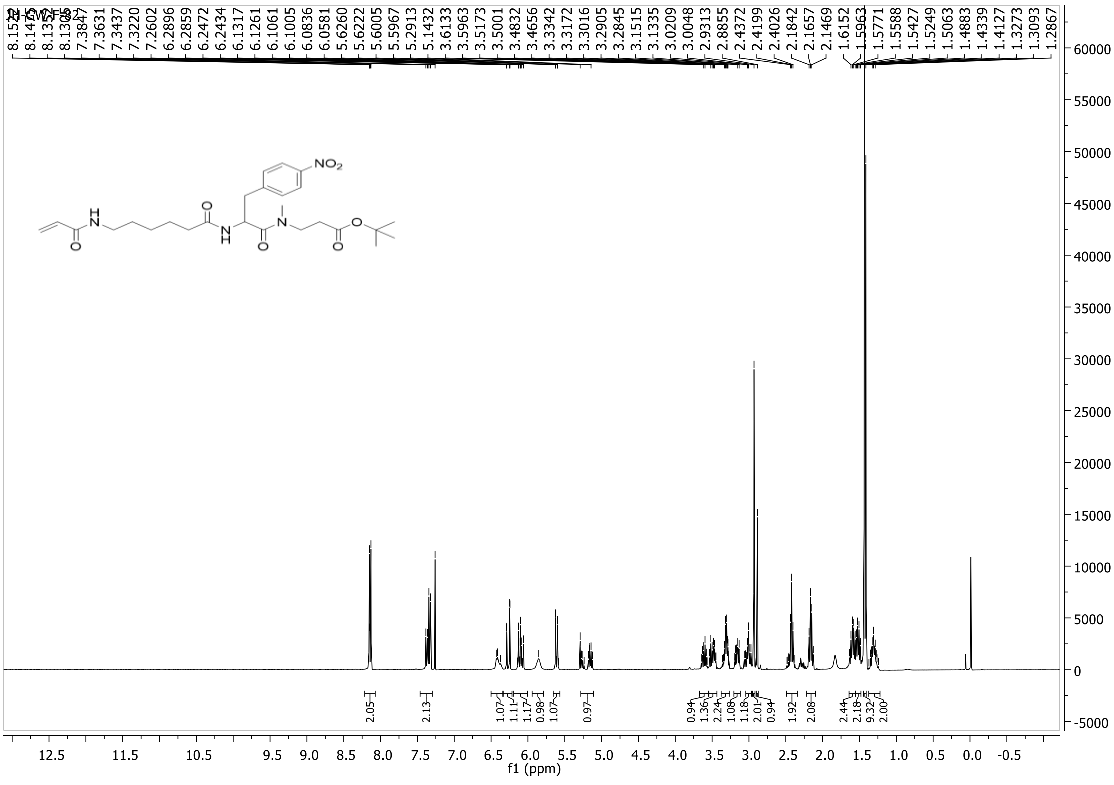

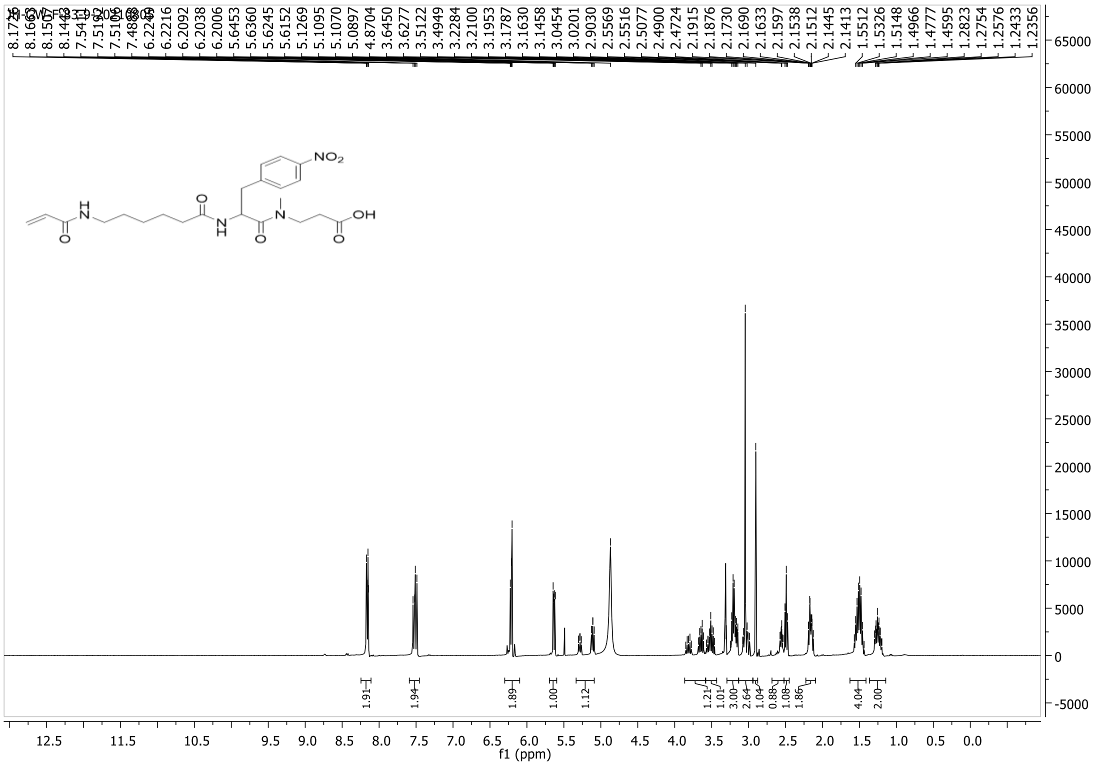
6.2216
6.2092
6.2038
6.2006
5.6453
5.6360
5.6245
5.6152
5.1269
5.1095
5.1070
5.0897
4.8704
3.6450
3.6277
3.5122
3.4949
3.2284
3.2100
3.1953
3.1787
3.1630
3.1458
3.0454
3.0201
2.9030
2.5569
2.5516
2.5077
2.4900
2.4724
2.1915
2.1876
2.1730
2.1690
2.1633
2.1597
2.1538
2.1512
2.1445
2.1413
1.5512
1.5326
1.5148
1.4966
1.4777
1.4595
1.2823
1.2754
1.2576
1.2433
1.2356
NO2
H
N
O
N
H
O
N
O
OH
1.91
1.94
1.89
1.00
1.12
1.21
1.01
3.00
2.64
1.04
0.88
1.08
1.86
4.04
2.00
f1 (ppm)

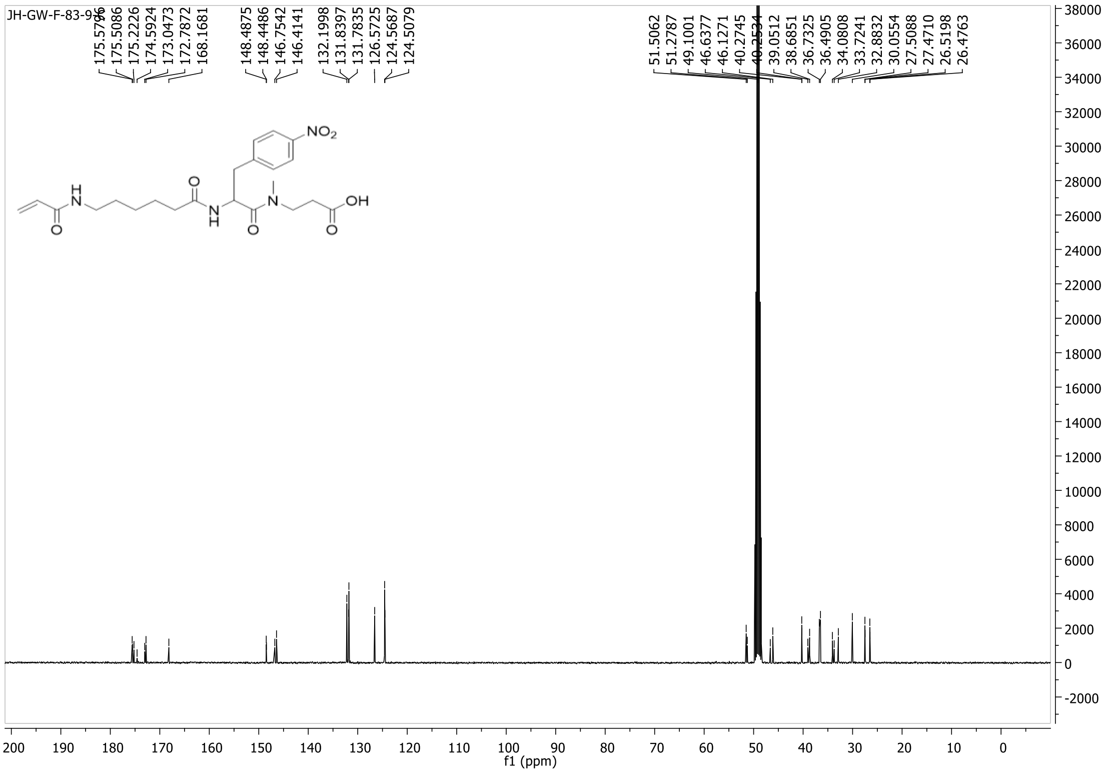
175.5086
175.2226
174.5924
173.0473
172.7872
168.1681
148.4875
148.4486
146.7542
146.4141
132.1998
131.8397
131.7835
126.5725
124.5687
124.5079
51.5062
51.2787
49.1001
46.6377
46.1271
40.2745
39.0512
38.6851
36.7325
36.4905
34.0808
33.7241
32.8832
30.0554
27.5088
27.4710
26.5198
26.4763
NO2
H
N
O
N
H
O
N
O
OH
f1 (ppm)

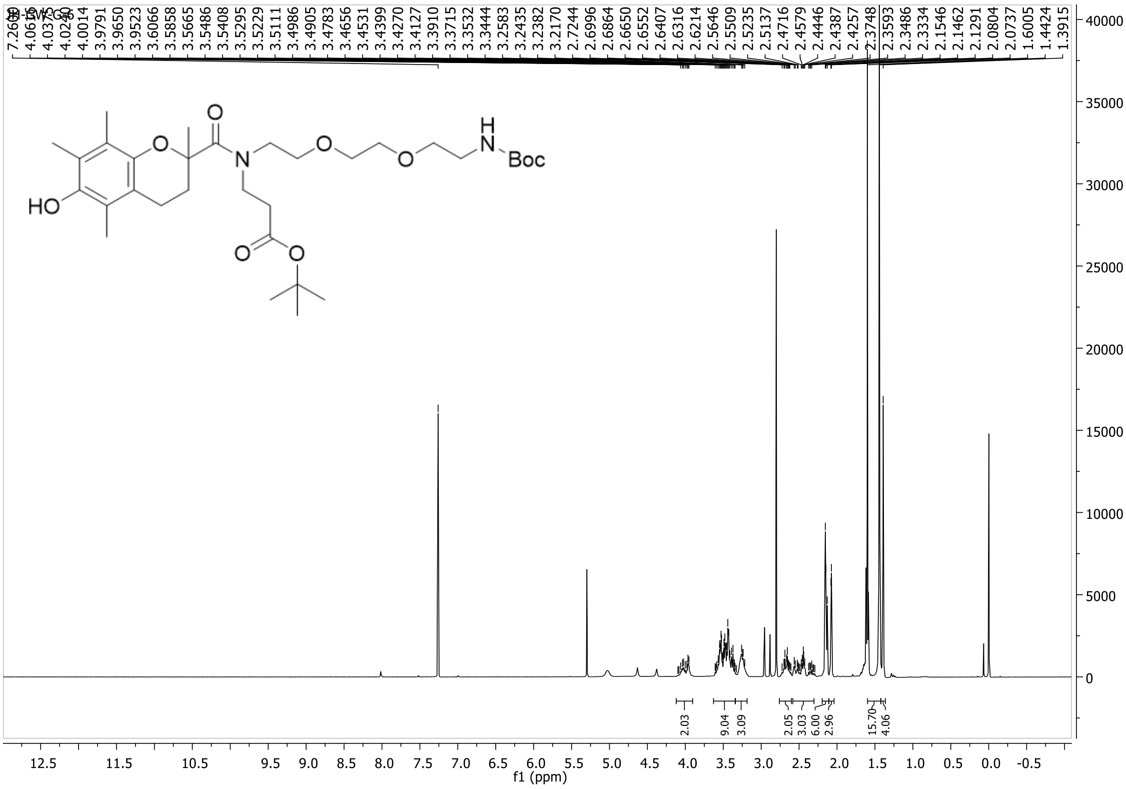
4.0014
3.9791
3.9650
3.9523
3.6066
3.5858
3.5665
3.5486
3.5408
3.5295
3.5229
3.5111
3.4986
3.4905
3.4783
3.4656
3.4531
3.4399
3.4270
3.4127
3.3910
3.3715
3.3532
3.3444
3.2583
3.2435
3.2382
3.2170
2.7244
2.6996
2.6864
2.6650
2.6552
2.6407
2.6316
2.6214
2.5646
2.5509
2.5235
2.5137
2.4716
2.4579
2.4446
2.4387
2.4257
2.3748
2.3593
2.3486
2.3334
2.1546
2.1462
2.1291
2.0804
2.0737
1.6005
1.4424
1.3915
HO
O
N
H
Boc
2.03
9.04
3.09
2.05
3.03
6.00
2.96
15.70
4.06
f1 (ppm)

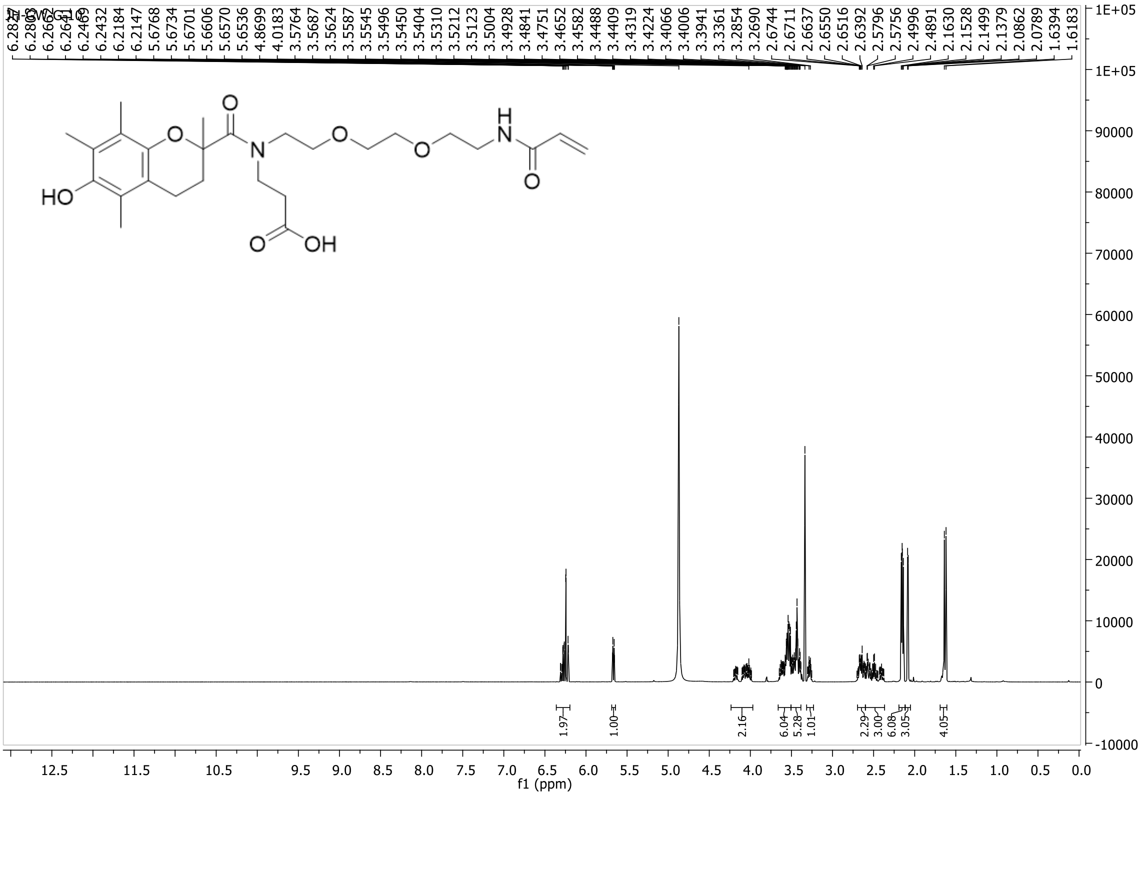
6.2469
6.2432
6.2184
6.2147
5.6768
5.6734
5.6606
5.6570
5.6536
4.8699
4.0183
3.5764
3.5687
3.5624
3.5587
3.5545
3.5496
3.5450
3.5404
3.5310
3.5212
3.5123
3.5004
3.4928
3.4841
3.4751
3.4652
3.4582
3.4488
3.4409
3.4319
3.4224
3.4066
3.4006
3.3941
3.3361
3.2854
3.2690
2.6744
2.6711
2.6637
2.6550
2.6516
2.6392
2.5796
2.5756
2.4996
2.4891
2.1630
2.1528
2.1499
2.1379
2.0862
2.0789
1.6394
1.6183
HO
O
N
H
OH
1.97
1.00
2.16
6.04
5.28
1.01
2.29
3.00
6.08
3.05
4.05
f1 (ppm)

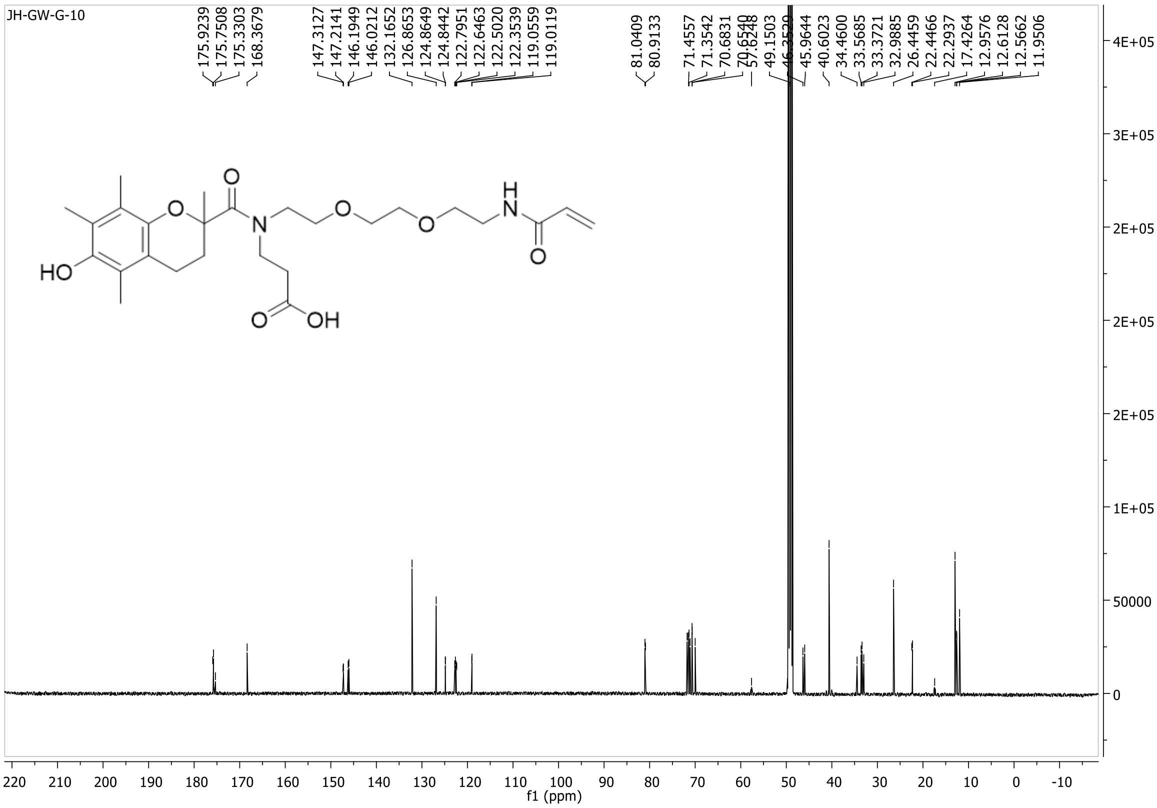

References

[1] G. Wen, M. Vanheusden, V. Leen, T. Rohand, K. Vandereyken, T. Voet, J. Hofkens, *J. Am. Chem. Soc.* **2021**, 143, 13782-13789.

[2] G. Wen, M. Vanheusden, A. Acke, D. Valli, R. K. Neely, V. Leen, J. Hofkens, *ACS Nano* **2020**, *14*, 7860-7867.

[3] L. Sun, J. Ding, W. Xing, Y. Gai, J. Sheng, D. Zeng, *Bioconjugate Chem.* **2016**, *27*, 1200-1204.
